# Management of antithrombotic therapies in kidney transplantation candidates and recipients: an ERBP clinical practice document by the DESCaRTES Working Group of ERA and the EKITA Committee of ESOT

**DOI:** 10.1093/ndt/gfag024

**Published:** 2026-02-09

**Authors:** Arzu Velioglu, Artemios G Karagiannidis, Erol Demir, Rachel Hellemans, Ilaria Gandolfini, Rachel Thomas, Annelis de Weerd, Ivana Dedinska, Andreas Kousios, Matej Samoš, Reyhan Diz Küçükkaya, Helen Erlandsson, Adnan Sharif, Mario Schiffer, Christophe Mariat, Luuk Hilbrands

**Affiliations:** Department of Nephrology, Marmara University School of Medicine, Istanbul, Türkiye; First Department of Nephrology, Hippokration Hospital, Aristotle University of Thessaloniki, Thessaloniki, Greece; Transplant Immunology Research Centre of Excellence, Koç University Hospital, Istanbul, Türkiye; Department of Nephrology-Hypertension, Antwerp University Hospital, Edegem, Belgium; Faculty of Medicine & Health Sciences, Laboratory of Experimental Medicine and Paediatrics (LEMP), University of Antwerp, Wilrijk, Belgium; Nephrology Unit, University Hospital of Parma, Parma, Italy; Edinburgh Transplant Centre, Royal Infirmary of Edinburgh, Edinburgh, UK; Department of Internal Medicine, Erasmus MC Transplant Institute, University Medical Center Rotterdam, Rotterdam, The Netherlands; Transplant-Nephrology Department Centre, Jessenius Faculty of Medicine, Comenius University and University Hospital Martin, Martin, Slovakia; School of Medicine, European University of Cyprus, Engomi, Cyprus; 1st Department of Internal Medicine, Jessenius Faculty of Medicine, University Hospital Martin, Comenius University in Bratislava, Martin, Slovakia; Department of Molecular Biology and Genetics, İstanbul University Faculty of Science, Istanbul, Türkiye; Department of Clinical Science, Intervention and Technology, Division of Renal Medicine, Karolinska Institutet, Stockholm, Sweden; Department of Nephrology and Transplantation, University Hospitals Birmingham, Queen Elizabeth Hospital, Birmingham, UK; Department of Nephrology and Hypertension, University Hospital Erlangen, Friedrich-Alexander University Erlangen-Nürnberg, Erlangen, Germany; Service de Néphrologie, Dialyse et Transplantation Rénale, Hôpital Nord, CHU de Saint-Etienne, Université Jean Monnet, Saint-Etienne, France; Department of Nephrology, Radboud University Medical Center, Nijmegen, The Netherlands

**Keywords:** anticoagulation, antiplatelet agents, antithrombotic therapy, kidney transplant candidates, kidney transplant recipients

## Abstract

Thromboembolic and atherosclerotic disorders, necessitating antithrombotic therapies, are prevalent among patients with advanced chronic kidney disease (CKD), as well as in kidney transplant (KT) recipients. However, the optimal management of antithrombotic therapies in these patients remains uncertain due to conflicting evidence and the exclusion of advanced CKD and KT recipients from major randomized controlled trials. Additionally, the perioperative management of antithrombotic therapy in end-stage kidney disease patients undergoing transplantation requires specific attention. This clinical practice document prepared by experts from the DESCaRTES Working Group of the European Renal Association (ERA) and European Kidney Transplant Association (EKITA) from European Society of Organ Transplantation (ESOT) following European Renal Best Practice (ERBP) guidelines is designed to support clinicians in decision making on antithrombotic therapies in KT candidates and recipients. Following a detailed systematic literature review, it presents current knowledge in epidemiology and pathophysiology and summarizes all relevant studies in this topic.

## INTRODUCTION

Patients with end-stage kidney disease (ESKD), especially those requiring dialysis, are at high risk for cardiovascular events, including myocardial infarction, stroke, and other thromboembolic events such as deep vein thrombosis and pulmonary embolism [1]. Despite uncertainties regarding the efficacy and safety of antiplatelet or anticoagulant drugs in patients with chronic kidney disease (CKD) stage 4–5, many of these patients receive antithrombotic therapies [2]. Even though the extensive transplant evaluation process sometimes excludes ESKD patients with severe comorbid conditions requiring antithrombotic therapies from waitlisting, currently the use of antiplatelet or anticoagulant drugs has not been accepted as a contraindication for kidney transplantation. Indeed, recent KDIGO guidelines for kidney transplant (KT) candidates recommend that ESKD patients should not be excluded from consideration for transplantation due to their use of antiplatelets or anticoagulants *per se* [3]. Nevertheless, due to broad prescription of antithrombotic drugs, the perioperative and early post-transplant management of hemostasis has become increasingly difficult.

Additionally, in the post-transplant period antiplatelet or anticoagulant drugs are often prescribed. In patients with atrial fibrillation on dialysis, anticoagulant drugs may be (re)started after transplantation. Moreover, rates of newly diagnosed cerebrovascular events and myocardial infarction, requiring antithrombotic therapies, are reported up to 6.8% and 11.1%, respectively, within 3 years post-KT [4–6]. The incidence of major cardiovascular adverse events (defined as myocardial infarction, stroke, critical peripheral ischemia and instable angina) occurs in 34% of living donor KT recipients within five years according to a Swedish study [7]. In parallel, almost 9% of KT recipients developed venous thromboembolism (VTE) within 5 years posttransplant, which is a seven-fold higher risk as compared with the general population [8]. These elevated risks underscore a need for guidance on antithrombotic therapy dosing, drug–drug interactions, and tailored strategies for high-bleeding-risk procedures like allograft biopsies. Yet, the evidence base remains limited, as most large clinical trials assessing antithrombotic efficacy and safety have excluded dialysis patients and KT recipients, leaving a critical gap in robust, transplant-specific recommendations.

This clinical practice document aims to help joint decision-making around management of antithrombotic therapies in KT candidates and recipients. The document summarizes current knowledge of the pathophysiology of increased bleeding and thrombosis risk in ESKD patients who are KT candidates, in ESKD patients undergoing transplant surgery and KT recipients. Additionally, it provides a comprehensive summary of the current evidence on peri-operative antithrombotic therapy management in adult KT candidates, explores the available data on safety and efficacy of antithrombotic therapies in KT recipients and examines the need for interruption of antithrombotic therapy prior to allograft biopsy.

## PATHOPHYSIOLOGY OF BLEEDING AND THROMBOSIS IN ESKD

ESKD has a profound influence on the hemostatic system. Alone or combined with kidney replacement therapy, it disrupts the fine balance between pro-coagulation and anticoagulation mechanisms, resulting simultaneously in both increased bleeding and thrombotic events [9]. Presently, the delineation of the patients exhibiting a predisposition toward either thrombosis or bleeding remains indeterminate.

### Bleeding risk

ESKD patients have an elevated risk of clinically significant bleeding, which is primarily attributed to the uremia-associated bleeding diathesis, but may also be influenced, at least in part, by the administration of antithrombotic therapies [9]. The interplay between altered coagulation cascades, dysregulated platelet function and abnormalities in the endothelium contributes to an increased risk of bleeding events [9–11].

It is well known that diminished platelet activation and adhesion caused by uremia increases the risk of bleeding. These platelets present with decreased expression and functional impairment of glycoprotein Ib and IIb/IIIa receptors, resulting in reduced release of alpha-granules [12]. In addition, the uremic milieu alters the production of arachidonic acid and prostaglandin compounds by the platelets, therefore leading to decreased synthesis of thromboxane A2 [13, 14]. In anemic ESKD patients, additional factors amplify this risk: reduced adenosine diphosphate release, aberrant prostaglandin I2 activation and diminished platelet-vessel wall interaction, compounded by elevated nitric oxide levels that inhibit aggregation [15].

Given that ESKD patients are at high risk of cardiovascular disease, they are commonly prescribed antiplatelet drugs, thereby increasing their risk of bleeding [10]. In addition, some anticoagulants are eliminated through the kidneys and therefore require timely dose adjustments to protect against bleeding events.

### Thrombosis risk

In addition to the aforementioned increased bleeding risk, ESKD patients are also paradoxically at risk of thromboembolic events [16]. This is mainly due to the activation of the renin–angiotensin–aldosterone system and endothelial cell damage accompanied by higher levels of fibrinogen and low levels of circulating fibrinolytic factors, secondary to chronic inflammation [17]. Clinically significant thrombosis in ESKD patients commonly manifests as deep venous thrombosis, pulmonary embolism or vascular access–associated thrombosis [18]. Furthermore, arterial thrombosis, frequently associated with atherosclerosis, presents as acute coronary syndrome, cerebrovascular events or peripheral artery disease [19]. It should also be noted that antiphospholipid syndrome, severe nephrotic syndrome and hereditary thrombophilic disorders can yield recurrent and severe thrombotic complications.

### Effect of kidney replacement therapies on hemostasis

Although hemodialysis (HD) improves platelet function by removing uremic toxins, it may paradoxically worsen bleeding tendencies by causing platelet dysfunction secondary to the dialysis membrane components [20]. HD patients also have an increased thrombotic risk due to chronic inflammation and a multitude of procoagulant factors: contact between FXII and artificial surfaces, e.g. dialysis membranes (mainly those with negative charge), activates the intrinsic coagulation pathway, resulting in thrombin production and fibrin formation [21]. Thus, cross-striated fibrin is deposited on the dialysis membrane, leading to mechanical trapping and damage of platelets passing through, ultimately contributing to transient thrombocytopenia during the session. Additionally, platelets are directly exposed to several activating forces during HD. Contact with dialysis membranes and mechanical stress from the roller pump induces platelet degranulation and surface glycoprotein loss [22]. Furthermore, variations in blood flow within the circuit might also induce the formation of microbubbles that further promote intradialytic platelet activation [23]. Additional aspects of HD, such as biocompatibility issues or complement activation, may impair platelet function or reduce their responsiveness. Consequently, because of excessive intradialytic activation and functional impairment, platelets are frequently and uncontrollably activated during HD, a fact that leads to thrombocytopenia during HD sessions or post-dialysis thrombocytopathy, often termed “exhausted platelets” [23].

To reduce the risk of clotting of the dialysis filter and prevent vascular access thrombosis, heparin or unfractionated low-molecular weight heparin is commonly administered during dialysis sessions. Prolonged heparin administration in dialysis patients may cause heparin-induced thrombocytopenia (HIT) type II, which promotes platelet activation and thrombosis.

Peritoneal dialysis patients may also present with hemostatic disruptions. Studies have shown higher levels of prothrombotic proteins [apolipoprotein (a), plasminogen activator inhibitor type 1, fibrinogen, coagulation factors II, VII, VIII, IX, X, XI and XII] in peritoneal dialysis patients compared with HD patients, a fact that may contribute to a higher risk for thrombotic events [24].

## PATHOPHYSIOLOGY OF BLEEDING AND THROMBOSIS DURING KIDNEY TRANSPLANTION SURGERY

Kidney transplantation surgery is associated with a high risk of bleeding, which can impair graft performance and overall patient outcomes. The incidence of severe post-transplantation bleeding has been estimated at 4%, a rate lower than that for other solid organ transplantations [25, 26]. Apart from pre-existing risk factors for bleeding, dissection of poor-quality vessels and hemodilution can also exacerbate a bleeding tendency. Achieving good surgical hemostasis is critical to reduce the risk of post-operative bleeding, prevent reoperation, blood transfusions and prolonged hospitalization. Apart from the direct effects of bleeding on graft and patient outcomes, the necessity for blood transfusion due to peri-operative bleeding may cause inadvertent human leukocyte antigen sensitization [27]. To minimize blood loss, local administration of hemostatic agents may ameliorate challenging complex bleeding during KT surgery. They are available in various forms, including gauze, mesh, solutions, sponges, powders, foams and gels, and can feature thrombin and fibrin compounds of increasing sophistication. Future developments would include nanotechnology to ensure accurate delivery [28].

Renal graft thrombosis is a feared complication of KT surgery that affects <1% of transplants [25]. Ischemia–reperfusion injury, prolonged cold ischemic time, vascular clamping and kinking of the arteries promote thrombosis through endothelial injury. Risk of thrombosis can be further increased by the presence of specific underlying diseases, such as hereditary thrombophilia and antiphospholipid syndrome [29].

A thorough preoperative evaluation of the patient’s coagulation profile (platelet count, prothrombin time, activated partial thromboplastin time) and need for antithrombotic therapies are critical components of a multifaceted approach to balance bleeding and thrombosis risk. It is advisable that anticoagulation specialists be consulted before transplantation in patients with increased risk for thrombosis or bleeding, including patients with a medical history of recurrent thrombosis, antiphospholipid syndrome or hemophilia to ensure safe transplantation surgery and to support recipient recovery and well-being.

## PATHOPHYSIOLOGY OF BLEEDING AND THROMBOSIS IN KT RECIPIENTS

Kidney transplantation appears to restore the balance between bleeding and thrombosis risks in most recipients. However, pre-existing diseases, use of antiplatelet or anticoagulant drugs and poor graft function can destabilize this fragile balance. Complement activation triggered by donor-specific antibodies, is often linked to antibody-mediated rejection and can contribute to the procoagulant response by activating thrombin [30]. Previous studies also showed that cytomegalovirus infection, post-transplant erythrocytosis, treatment with high-dose steroids and previous thrombotic events can increase the risk of thrombosis in KT recipients [31]. Using antithrombotic therapies, kidney allograft biopsy may be sometimes delayed due to a need for preprocedural discontinuation of the antithrombotic drugs.

**Figure 1:**
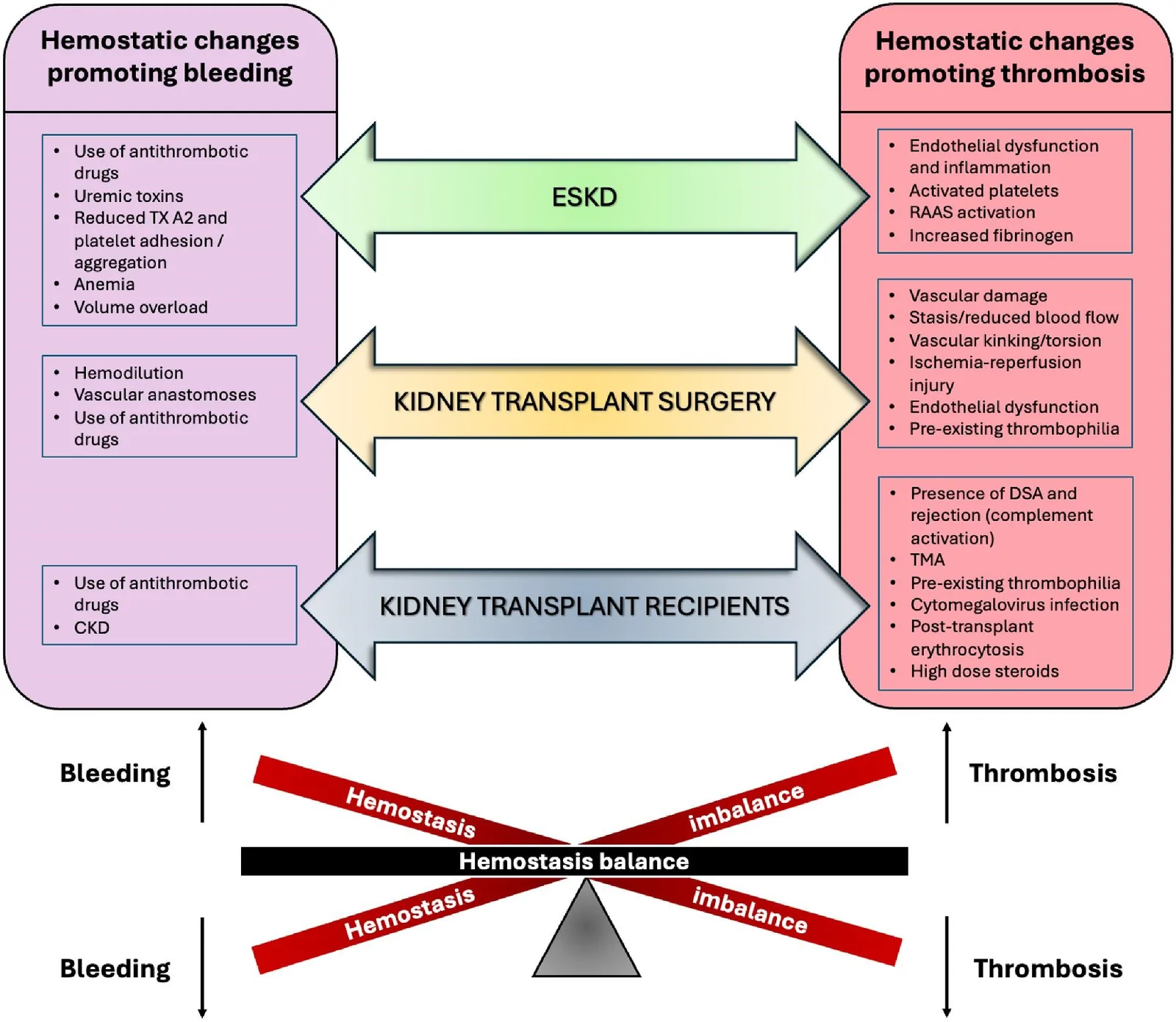
Hemostatic balance in ESKD, transplant surgery and KT recipients.

Figure 1 summarizes hemostatic changes in KT candidates, patients undergoing KT surgery and KT recipients.

## ANTITHROMBOTIC THERAPIES

### Antiplatelet agents

Antiplatelet agents, which include acetylsalicylic acid (ASA) and P2Y_12_ inhibitors like clopidogrel, ticagrelor and prasugrel, are used to prevent thromboembolic events, particularly in individuals with atherosclerotic vascular diseases, and are commonly prescribed in ESKD patients and KT recipients [32], although data on their efficacy and safety in these specific populations are lacking. ASA irreversibly inhibits cyclooxygenase-1 (COX-1), thereby impeding the synthesis and release of thromboxane A2 (TXA2), a potent platelet activator. The drug oral bioavailability typically ranges from 40% to 50%, with peak plasma concentrations usually within 30–40 min following ingestion (3–4 h for enteric-coated formulations). ASA antiplatelet effects persist for the lifespan of platelets (7–10 days). Given that around 10% of the platelet population regenerates daily, once-daily dosing is generally sufficient to maintain near-complete inhibition [32].

Clopidogrel, a second-generation thienopyridine prodrug, requires intestinal absorption and hepatic metabolism to generate an active metabolite that irreversibly binds to the P2Y_12_ receptor on platelets for their entire lifespan. Kidney dysfunction markedly hampers the drug’s biotransformation process, while genetic polymorphisms affecting absorption and cytochrome P450 (CYP) enzymes contribute to unpredictable variations in platelet reactivity [33]. In contrast, ticagrelor, a third-generation P2Y_12_ blocker, binds extensively (approximately 99.8%) to plasma proteins without requiring metabolic activation. It achieves maximal platelet inhibition in about 2 h, with effects waning after 12 h , necessitating twice-daily dosing. A study has shown that compared with clopidogrel, ticagrelor consistently achieves more effective P2Y_12_ inhibition, resulting in a lower incidence of high on-treatment platelet reactivity and drug non-responsiveness [34]. Prasugrel, another third-generation P2Y_12_ blocker, carries a higher bleeding risk, especially in older adults (>75 years), low body weight (<60 kg) and those with previous stroke/transient ischemic attack [35].

Ticlopidine is a thienopyridine class antiplatelet agent that irreversibly inhibits the P2Y_12_ receptor on platelets. The potential for bone marrow suppression renders it less advantageous for KT recipients who already have a high risk of depleted bone marrow due to immunosuppressive drugs.

Dipyridamole is a phosphodiesterase inhibitor that increases intracellular cAMP levels, leading to inhibition of platelet activation and vasodilation. It also inhibits adenosine uptake, enhancing vasodilatory and antithrombotic effects. There are more effective drugs for prevention of thrombotic events, so it is less favorable, but it is safe in CKD, as it does not require renal dose adjustments.

Table 1 summarizes the mechanisms, pharmacodynamics and KT-specific considerations for oral antiplatelet drugs.

**Table 1:** Oral antiplatelets: mechanism of action, pharmacodynamics and special considerations for KT recipients.

	**Aspirin**	**Clopidogrel**	**Ticagrelor**	**Prasugrel**	**Ticlopidine**	**Dipyridamole**
Mechanism of action	COX-1 inhibitor (direct, irreversible)	ADP P2Y_12_ inhibitor (prodrug, irreversible)	ADP P2Y_12_ inhibitor (direct acting, reversible)	ADP P2Y_12_ inhibitor (prodrug, irreversible)	ADP P2Y_12_ inhibitor (prodrug, irreversible)	Phosphodiesterase inhibitor (↑ cAMP, vasodilatory effect)
Half-life	15–20 min (but platelet inhibition lasts 7–10 days)	6 h (active metabolite effect ~24 h)	7–9 h	7–8 h	12–30 h	10–12 h
Time from last dose to offset	5–7 days (irreversible COX-1 inhibition)	5–7 days (new platelet formation needed)	3–5 days (reversible inhibition)	7–10 days (irreversible inhibition)	7–10 days (irreversible inhibition)	~24 h (shorter effect)
Bioavailability	40%–50% (first-pass metabolism reduces absorption)	>50%	36%	>80%	80%	70%
Recommended dose and frequency	75–100 mg (OD)	300 mg loading dose, then 75 mg OD	90 mg (BID)	10 mg (OD)	250 mg BID	Immediate release: 50–100 mg TID/QIDExtended release: 200 mg BID (with aspirin)
Renal excretion	6%–36%	50%	Minimal renal excretion	70%	40%	1%–10%
Interactions	↑ Bleeding risk with NSAIDs, anticoagulants ↓ Effect with ibuprofen (competitive COX-1 inhibition)	↓ Effect with CYP2C19 inhibitors (e.g. PPIs)^a^	↓ Effect with CYP3A4 inducers^b^/↑ effect with CYP3A4 inhibitors		↓ Effect with CYP2C19 inhibitors^d^	↑ Hypotensive effect of antihypertensivesInterferes with adenosine clearance
Dialysable/reversal agent	Partially yes/no	No/no	No/no	Unknown/no	No/no	No/aminophylline (to reverse vasodilation in stress testing)
Precautions for transplantation recipients	CNIs: no clinically relevant interaction Mycophenolate: ↑ exposure (due to renal tubular competition)^e^	CNIs: no clinically relevant interaction Mycophenolate: no clinically relevant interaction^e^	CNIs: cyclosporine ↑ ticagrelor exposure; tacrolimus—no relevant effect Mycophenolate: no clinically relevant interaction^b^	CNIs: no clinically relevant interaction Mycophenolate: no clinically relevant interaction^c^	CNIs: no clinically relevant interaction Mycophenolate: ↑ exposure (due to renal tubular competition)^e^ Not recommended due to hematological toxicity (neutropenia, thrombocytopenia, TMA)	No

BID, bis in die (twice daily); cAMP, cyclic adenosine monophosphate; COX-1, Cyclooxygenase-1; CYP2C19, Cytochrome P450 2C19; CYP3A4, Cytochrome P450 3A4; NSAIDs, nonsteroidal anti-inflammatory drugs; OD, once daily (omni die); PPIs, proton pump inhibitors; QID, quater in die (four times daily); TID, ter in die (three times daily); TMA, thrombotic microangiopathy. References:

a Gilard M, Arnaud B, Cornily JC*. et al*. Influence of omeprazole on the antiplatelet action of clopidogrel associated with aspirin: the randomized, double-blind OCLA (Omeprazole CLopidogrel Aspirin) study. *J Am Coll Cardiol* 2008;51:256–60 (in English); doi: 10.1016/j.jacc.2007.06.064.

b Teng R. Ticagrelor: pharmacokinetic, pharmacodynamic and pharmacogenetic profile: an update. *Clin Pharmacokinet* 2015;54:1125–38 (in English); doi: 10.1007/s40262-015-0290-2.

c Ferri N, Corsini A, Bellosta S. Pharmacology of the new P2Y12 receptor inhibitors: insights on pharmacokinetic and pharmacodynamic properties. *Drugs* 2013;73:1681–709 (in English); doi: 10.1007/s40265-013-0126-z.

d Tateishi T, Kumai T, Watanabe M. *et al*. Ticlopidine decreases the in vivo activity of CYP2C19 as measured by omeprazole metabolism. *Br J Clin Pharmacol* 1999;47:454–7 (in English); doi: 10.1046/j.1365-2125.1999.00914.x.

e Ivanyuk A, Livio F, Biollaz J. *et al*. Renal drug transporters and drug interactions. *Clin Pharmacokinet* 2017;56:825–92 (in English); doi: 10.1007/s40262-017-0506-8.

The main indications for antiplatelets in KT recipients include: (i) secondary prevention of atherosclerotic cardiovascular disease, e.g. prior myocardial infarction, acute coronary syndrome, ischemic stroke or transient ischemic attack, and peripheral arterial disease, (ii) post-percutaneous coronary intervention (PCI) with stent or post-percutaneous intervention of peripheral arterial disease, and (iii) prevention of early or acute renal allograft thrombosis in selected high-risk patients in specific centers. However, and unlike other populations, primary prevention is not considered safe choice in KT recipients, due to a poor risk–benefit ratio.

Regarding the choice of antiplatelet, low-dose ASA is the preferred first-line agent in most ESKD patients and KT candidates and recipients, given its well-known pharmacology. Clopidogrel is generally the P2Y₁₂ inhibitor of choice when dual antiplatelet therapy (DAPT) is needed (e.g. after acute coronary syndrome) or when ASA is contraindicated. Based on pharmacological profiles of P2Y_12_ inhibitors, ticagrelor requires caution with calcineurin inhibitors (CNIs), particularly with cyclosporin, due to interaction risks. In contrast, such interactions are not noted for clopidogrel or prasugrel (Table 1).

Before initiation of drugs, a full blood count, serum creatinine and estimated glomerular filtration rate (eGFR) (particularly in KT recipients), liver function tests, and a detailed bleeding and thrombotic history, including prior gastrointestinal bleeding and intracranial hemorrhage, should be taken. After initiation of therapy, patients should be closely monitored for signs of bleeding (petechiae, easy bruising, epistaxis, gastrointestinal bleeding, hematuria, menorrhagia), as well as monitoring of hemoglobin/hematocrit and platelet count. In parallel, monitoring of CNI trough levels is needed when ticagrelor is used.

### Anticoagulant agents

Anticoagulants encompass four main classes of agents: heparins [unfractionated heparin (UFH), low molecular weight heparin (LMWH) and synthetic pentasaccharide (fondoparinux)], vitamin K antagonists (e.g. warfarin, acenocoumarol and phenprocoumon), hirudin derivatives (argatroban, bivaluridin and lepirudin) and direct-acting oral anticoagulants. UFH potentiates the activity of antithrombin, which in turn inhibits several coagulation factors, notably factor Xa and factor IIa (thrombin). UFH is generally administered as a continuous intravenous infusion. It has a short half-life of approximately 30 min and can be administered safely to patients with CKD, although those with ESKD necessitate a 33% dose reduction [36]. Its effects can be rapidly and completely reversed by protamine sulfate. Clearance predominantly occurs via endothelial cells and the reticuloendothelial system, with a minor contribution from renal excretion [37]. Adverse effects of UFH include bleeding and thrombocytopenia, with the latter occasionally presenting as HIT, a condition associated with paradoxical thrombosis. UFH’s unpredictable pharmacokinetics necessitates monitoring via activated partial thromboplastin time. LMWHs, such as enoxaparin, bemiparin, dalteparin, tinzaparin and nadroparin, also inhibit factor Xa, although they exhibit less efficacy against thrombin compared with UFH. LMWHs have a longer half-life of approximately 4–6 h, better pharmacokinetic properties, and require less frequent monitoring than UFH. Nonetheless, renal elimination necessitates dose adjustment, particularly in advanced CKD patients, owing to an increased risk of bleeding. Routine anti-Xa monitoring is not required in most patients receiving standard weight-based dosing. However, monitoring is recommended in special populations or when dosing deviates from standard protocols, including patients with impaired kidney function, obesity, low body weight, pregnancy and the elderly [36]. Protamine is the widely used and accepted antidote for LMWH, though it does not completely reverse anti-Xa activity.

Vitamin K antagonists (VKAs) such as warfarin, acenocoumarol and phenprocoumon, inhibit the vitamin K epoxide reductase complex 1 and impair the synthesis of coagulation factors II, VII, IX and X, and anticoagulant proteins C and S. They also affect bone metabolism via osteocalcin and matrix Gla protein. The anticoagulant effect of VKAs begins approximately 72–96 h after administration, as the vitamin K–dependent coagulation factors become depleted, and the therapeutic window is relatively narrow. Warfarin’s half-life ranges from 24 to 58 h (shorter for acenocoumarol, longer for phenprocoumon). VKAs interact with numerous foods and medications. The quality of anticoagulation is typically assessed through the time in therapeutic range (TTR). However, maintaining an adequate TTR is particularly challenging in dialysis and KT recipients due to fluctuating volume status, changes in nutritional intake, frequent antibiotic exposure and interactions with immunosuppressive agents. Studies have consistently shown that ESKD patients have markedly lower TTR values compared with the general population, leading to higher rates of both subtherapeutic (increasing thrombosis risk) and supratherapeutic (increasing bleeding risk) anticoagulation [38, 39]. Additionally, VKAs have been implicated in promoting vascular calcification progression by inhibiting the vitamin K–dependent matrix Gla protein, which normally suppresses vascular calcification [40], and have also been shown to decrease bone mineral density, leading to an increased risk of fractures [41]. Despite these concerns, many KT recipients with cardiovascular comorbidities may remain on a VKA, although it should be discontinued or bridged with heparin before transplantation surgery [18]. The reversal of VKAs is achieved using vitamin K and, in cases of major bleeding or urgent procedures, prothrombin complex concentrate or fresh frozen plasma may be administered for rapid correction.

Lastly, direct oral anticoagulants (DOACs) represent a more recent advancement in anticoagulation therapy, targeting either direct thrombin or factor Xa in the coagulation cascade. These agents include dabigatran (a direct thrombin inhibitor) and factor Xa inhibitors such as rivaroxaban, apixaban and edoxaban. While DOACs are generally considered safe for patients with CKD stages 1–3, their efficacy and safety in CKD stages 4 and 5 remain uncertain due to limited evidence [42]. Recently, only the use of apixaban was approved by the Food and Drug Administration in patients with GFR <15 mL/min and those on dialysis. Dabigatran is predominantly renally cleared (approximately 85%), rendering it contraindicated in CKD patients with GFR <60 mL/min. Factor Xa inhibitors, characterized by a half-life ranging from 5–12 h, undergo partial renal excretion, albeit to a lesser extent than dabigatran. However, severe CKD may prolong their half-life, necessitating cautious dosing adjustments [36]. There are agents that selectively reverse their actions, such as andexanet-alfa and idarucizumab which antagonize factor Xa inhibitors and dabigatran, respectively [43]. However, their bioavailability seems to be influenced by kidney impairment [44] and caution may be required in ESKD despite preliminary clinical data in CKD patients supporting their efficacy and safety [45].

During the last few years, novel FXI and FXIa inhibitors have emerged, including among others the monoclonal antibodies osocimab and abelacimab, which are not cleared by the kidneys and therefore offer an additional benefit in patients with ESKD [46]. In a recent meta-analysis these drugs did not display any significant increase in bleeding risk compared with placebo among patients with ESKD on HD [47]. However, their use in these patients remains quite limited, while there are currently no data in KT recipients.

Table 2 summarizes the oral anticoagulant agents, mechanisms of action, pharmacodynamics, pharmacokinetics and considerations for KT recipients.

**Table 2:** Oral anticoagulants: mechanism of action, pharmacodynamics and special considerations for KT recipients.

	Vitamin K antagonists	FXa inhibitors			Direct thrombin inhibitors
	Warfarin	Rivaroxaban	Apixaban	Edoxaban	Dabigatran
Mechanism of action	Inhibits hepatic synthesis of vitamin K–dependent coagulation factors	Direct inhibition of FXa	Direct inhibition of FXa	Direct inhibition of FXa	Direct inhibition of clot-bound and free thrombin (FIIa)
Half-life (h)	20–60	5–9 (9–13 in elderly)	8–13	9–11	14–17
Time from last dose to offset	3–5 day (INR dependent)	24–48 h (1–2 days)	48 h (2 days)	24–36 h (1–1.5 days)	48–72 h (prolonged in renal impairment)
Bioavailability (%)	100	80	66	50	6.5
Recommended dose and frequency	Adjusted based on INR; OD	20 mg OD	5 mg BID	30 or 60 mg OD	150 mg BID
Renal excretion (%)	1 (unchanged in the urine)	66	27	45	80
Interactions	↑ Effect with CYP2C9/1A2/3A4 inhibitors↓ Effect with high dietary vitamin K^a^	↑ Effect with potent CYP3A4 inhibitors and P-glycoprotein inhibitors^b^	↑ Effect with potent CYP3A4 inhibitors^b^	↑ Effect with P-glycoprotein inhibitors^b^	↑ Effect with P-glycoprotein inhibitors^b^
Dialysable/drug reversal	No/vitamin K, FFP, PCC	No (only edoxaban has some dialyzability but not highly effective)/andexanet alfa, PCC, 4F-PCC			Partially dialyzable/idarucizumab, PCC, 4F-PCC
Precautions for transplantation recipients	CNIs: tacrolimus has no clinically relevant interaction Cyclosporine may modestly increase warfarin effect^c^ Mycophenolate: no clinically relevant interaction	CNIs: cyclosporine markedly increases DOAC exposure; avoid with dabigatran Tacrolimus causes only modest, clinically limited increases Mycophenolate: no clinically relevant interaction Check GFR for DOAC dose modification Prolonged time after cessation is required for patients with low GFR before invasive procedures^d^			

4F-PCC, four-factor prothrombin complex concentrate; BID, bis in die (twice daily); CYP1A2, Cytochrome P450 1A2; CYP2C9, Cytochrome P450 2C9; CYP3A4, Cytochrome P450 3A4; FFP, fresh frozen plasma; FXa, Factor Xa; OD, once daily (omni die); PCC, prothrombin complex concentrate. References:

a Montomoli M, Candía BG, Barrios AA. *et al*. Anticoagulation in chronic kidney disease. *Drugs* 2024;84:1199–218 (in English); doi: 10.1007/s40265-024-02077-6.

b Ferri N, Colombo E, Tenconi M. *et al*. Drug-drug interactions of direct oral anticoagulants (DOACs): from pharmacological to clinical practice. *Pharmaceutics* 2022;14 (in English); doi: 10.3390/pharmaceutics14061120.

c Lam E, Bashir B, Chaballa M. *et al*. Drug interactions between direct-acting oral anticoagulants and calcineurin inhibitors during solid organ transplantation: considerations for therapy. *Expert Rev Clin Pharmacol* 2019;12:781–90 (in English); doi: 10.1080/17512433.2019.1637733.

d Lange NW, Muir J, Salerno DM. Direct oral anticoagulants in patients with ESRD and kidney transplantation. *Kidney Int Rep* 2025;10:40–53 (in English); doi: 10.1016/j.ekir.2024.10.016.

## SCORES FOR ASSESSING BLEEDING AND THROMBOTIC RISK

No bleeding or thrombosis risk score has been formally validated in patients with ESKD or in KT recipients/candidates. However, several widely used clinical tools, validated primarily in the general population and partly in advanced CKD, can support decision-making when considering the initiation or continuation of antiplatelet or anticoagulant drugs in this population.

Among bleeding risk scores, the HAS-BLED, originally developed for atrial fibrillation patients receiving anticoagulation, remains one of the most referenced tools [48]. Many of its components [e.g. hypertension, abnormal renal function, labile International Normalized Ratio (INR), age and concomitant antiplatelet use] are highly prevalent in ESKD patients; this significantly limits its discriminatory value. Nevertheless, it offers a structured framework for identifying modifiable bleeding risk factors (e.g. uncontrolled blood pressure, unnecessary DAPT), which can be clinically useful during pre-transplant evaluation or in the peri-operative period [49]. Other bleeding risk scores, ATRIA, HEMORR2HAGES and ORBIT, may not be useful in dialysis patients [50].

Similarly, thromboembolic risk assessment often relies on the CHA₂DS₂-VASc score, widely used for stratifying stroke risk in patients with atrial fibrillation [51]. Its predictive accuracy is reduced in ESKD, because uremia-related alterations in coagulation and vascular biology are not captured by the traditional scoring components. Nonetheless, CHA₂DS₂-VASc remains the most used tool to guide anticoagulation decisions in KT recipients with atrial fibrillation, as it helps quantify baseline thrombotic risk, even if absolute risk estimates may differ from the general population [52].

Clinicians interpreting these scores in ESKD and transplant populations must recognize their limited calibration and the need for individualizing clinical decisions.

## METHODOLOGY—SYSTEMATIC SEARCH

This clinical practice document was developed by the Developing Education Science and Care for Renal Transplantation in European States (DEScARTES) scientific working group of the European Renal Association (ERA) in collaboration with experts from European Kidney Transplant Association (EKITA) from the European Society for Organ Transplantation (ESOT) and other content experts (a cardiologist and a hematologist). The group was supplemented with a methodologist to supervise the project and provide methodological expertise. The group identified and constructed four clinical questions. (1) What is the peri-operative bleeding risk in KT candidates who are on antiplatelet or anticoagulant drugs? (2) Should KT recipients receive prophylaxis for venous thrombosis after surgery? (3) Can we use DOACs in KT recipients in terms of safety (dosing, drug–drug interactions, adverse events)? (4) Is there a need for interruption of antiplatelet therapy preceding graft biopsy? To provide evidence-based answers to these questions, a systematic review was performed according to Preferred Reporting Items for Systematic Reviews and Meta-Analyses (PRISMA) and ERBP principles. A detailed description of methodology is provided in the Supplementary Material.

## QUESTION 1: WHAT IS THE PERI-OPERATIVE BLEEDING RISK IN KT CANDIDATES WHO ARE ON ANTIPLATELET OR ANTICOAGULANT DRUGS?

Authors: Rachel Hellemans, Ilaria Gandolfini, Rachel Thomas, Artemios Karagiannidis, Arzu Velioglu

### Background and rationale

In order to optimize the safest care for KT candidates on antithrombotic therapies, clinicians should have a comprehensive knowledge of the possible consequences, such as risk of perioperative bleeding. There is little antithrombotic management data for KT surgery, mostly extrapolated from other specialties. In general, antiplatelet or anticoagulant drugs should be stopped to reduce the risk of bleeding complications, however, in deceased donor transplantation, an inherently unplanned surgery, there is often insufficient time for drug effects to subside, and delaying the procedure is undesirable due to concerns about cold ischemia time and graft viability. At the same time, interrupting antithrombotic therapy may increase the risk of thromboembolic events, especially in patients with coronary artery disease, a history of stroke and previous VTE. Therefore, we aimed at reviewing the impact of continuation or discontinuation of pretransplant antiplatelet or anticoagulant drugs on peri-transplant bleeding and thromboembolic complications.

### Summary of evidence

(Supplement | Study selection flow diagram for Question 1, see Supplementary data, Fig. S1)

(Supplement | Summary evidence table for Question 1, see Supplementary data, Table S10)

Given the highly diverse outcomes, definitions, and variable rates of complications in this systematic review, we had to review evidence categorized by the type of antithrombotic therapy. In any case, as the studies were heterogeneous in design, population and reported outcomes, overall risk of bias was high and certainty of evidence was low.

#### Antiplatelet therapies

Our search identified eight original studies (five full papers, three abstracts) and one systematic review and meta-analysis that investigated the impact of antiplatelet drugs on post-operative outcomes. These included a small randomized controlled trial and seven single-center retrospective cohort studies.

#### Antiplatelet monotherapy versus no antiplatelet therapy

##### Aspirin

Robertson *et al*. [53] found a significant risk reduction in rates of early renal vein graft thrombosis with perioperative ASA (pretransplant to 1-month posttransplant) as compared with a historical cohort without ASA, but noted early major bleeding events, although these were not compared with controls and confounding factors (e.g. patient selection, improved care), limiting the conclusions. Rodriguez-Pastore *et al.* [54] conducted a trial of 30 recipients on ASA at time of transplantation. The patients were randomized to receive a perioperative platelet transfusion or not and results were compared with a control group of 70 patients not on ASA. They found similar hemoglobin drop across all three groups, but higher rates of ultrasound-detected perirenal bleeding in ASA patients. The small sample size and limited data preclude firm conclusions.

##### Clopidogrel

Kapeles *et al*. [55] found significantly higher perioperative transfusion rates in pretransplant clopidogrel users, though details were limited making it difficult to assess the study’s reliability or relevance.

#### DAPT versus no antiplatelet therapy

Bansal *et al*. [56] reported a higher drain output in patients on DAPT at transplantation compared with patients who stopped DAPT 5 days pre-transplant, with other perioperative outcomes (blood loss, transfusion, hemoglobin drop and cardiovascular events) being similar between groups.

#### DAPT versus single antiplatelet therapy

A systematic review and meta-analysis by Lee *et al.* [57] including five retrospective studies [26, 58–61] showed DAPT increased post-operative hemorrhage risk almost by 60% in patients on DAPT compared with those on monotherapy; cardiovascular event rates were similar, though not quantitatively analyzed. The studies were small, heterogeneous and of low quality, with inconsistent definitions of hemorrhagic events, limiting the ability to assess the clinical relevance.

#### Mixed or unspecified antiplatelet therapies versus no therapy

Hernández *et al*. [62] reported higher hemorrhagic complication rates among patients on antiplatelet therapy at transplantation compared with controls, with multivariable analysis confirming this 1.8-fold higher odds of the outcome, but the lack of specification regarding the type of antiplatelet therapy weakens the study’s conclusions. Benahmed *et al.* [63] found similar rates of transfusion, reoperation or cardiovascular events between antiplatelet users (clopidogrel/ticlopidine ± ASA) and controls on no therapy; however, the sample was small and treatment variability limit the study’s conclusions.

#### Anticoagulant therapies

Our search identified five original studies (three full papers, two abstracts). These included a small randomized controlled trial, two retrospective cohort studies and two case series.

#### Heparin

Kikić *et al.* [64] randomized adult preoperative patients to different preoperative dialysis anticoagulation approaches (i.e. either heparin HD or no dialysis in case of pre-operative potassium ≤5 mEq/L, and to either citrate HD or heparin HD in case of preoperative potassium >5 mEq/L). They reported bleeding events in all groups, with no significant differences between heparin and citrate or no-heparin strategies. Despite moderate evidence quality, intradialytic heparin data does not generalize to anticoagulant use.

#### VKA—warfarin

Connaughton *et al*. [65] examined warfarin-treated recipients compared with sex-matched controls, reversing warfarin preoperatively per guidelines. No differences emerged in hemoglobin, graft or patient survival, but warfarin patients required more perioperative blood products (transfusion rates, fresh frozen plasma use). Interpretation is limited by the small size and poor matching. Similarly, Wong *et al.* [66] reported that warfarin users compared with matched controls tended to be older, with higher prevalence of ischemic heart disease, longer hospitalization and higher transfusion rates. Post-transplant anticoagulation increased reoperation risk. However, the study’s limited reporting substantially weakens the reliability of these findings.

#### DOACs—apixaban

Two case series reported the early post-transplant outcomes of deceased-donor recipients on pre-transplant apixaban anticoagulation therapy. Littlejohn *et al.* [67] described a small cohort with variable apixaban dosing (5 mg, *n* = 7; 2.5 mg, *n* = 5; high-dose with aspirin, *n* = 3) and noted several early bleeding events. Lange *et al*. [68] similarly evaluated a small number of KT recipients on apixaban with variable doses and no reversal agents. Blood loss ranged from 100 to 1600 mL and notable bleeding complications were observed (major vascular injury, posttransplant transfusions) with high bleeding rates. Taken together, studies suggest possible increased rates of bleeding with apixaban at the time of transplantation, yet variations in dosage, small sample sizes and the absence of control groups make it difficult to draw definitive conclusions.

#### Miscellaneous studies (mixed therapies of VKAs, unfractionated heparin, antiplatelet agents)

Additionally, we identified 11 studies (9 full papers, 2 abstracts). These included eight retrospective cohort studies, one case–control study and two abstract papers that compared a variety of antithrombotic therapies in patients undergoing kidney transplantation.

van den Berg *et al.* [25] investigated bleeding events within the first week post-transplant across multiple treatment groups (control, intraoperative heparin, continued VKA, continued antiplatelet therapy, combined antiplatelet and heparin) and found that continued VKA use was independently associated with an increased risk of post-operative bleeding, reporting more than 6-fold higher odds compared with controls. Similarly, Marzouk *et al.* [69] observed higher transfusion rates among patients receiving pre-operatively warfarin or antiplatelet agents compared with those not on such therapies, a finding that was also confirmed by Musetti *et al*. [59] reporting that patients receiving pre-transplant VKA had higher rates of post-operative bleeding compared with those receiving antiplatelet therapies and controls. In a more recent study, Hau *et al.* [49] also found that preoperative use of VKA significantly increased the risk of post-operative bleeding by almost six times, but antiplatelet agent use did not.

Weng *et al*. [61] analyzed KT recipients with coronary artery disease and found that patients using DAPT had higher transfusion rates at transplant, although rates of hematoma drainage were similar to recipients on other pre-transplant antithrombotic regimens.

In contrast, numerous studies did not demonstrate a clear increase in bleeding complications with antiplatelet or anticoagulant use. Eng *et al*. [70] reported no significant increase in reoperation, transfusions or hemoglobin drop in patients receiving preoperative antiplatelets or anticoagulants. Another retrospective study comparing recipients on antiplatelets or anticoagulants with controls found that ASA did not significantly alter bleeding risk, whereas anticoagulants were associated with a higher risk of bleeding events [71]. In parallel, Diamond *et al*. [72], examining a large cohort of KT recipients divided into three groups (anticoagulant users, antiplatelet users, controls), found no significant between-group differences in major bleeding or thromboembolic events. Alonso-Escalante *et al.* [73] found comparable rates graft re-exploration rates and perioperative transfusions between pre-transplant warfarin users, antiplatelet users and untreated controls.

A case–control study including recipients with and without post-operative hemorrhage found no clear association between pretransplant antiplatelet or anticoagulant use and post-operative hemorrhage risk [26]. Finally, Buggs *et al*. [74] found that recipients with post-operative bleeding requiring reoperation did not differ in their preoperative anticoagulation status from those who did not require re-operation.

Taken together, these studies consistently suggest that peri-operative use of VKAs is associated with a higher risk of bleeding complications, while DAPT may also increase transfusion needs at the time of KT.

### Translation of the evidence into clinical practice

The available evidence, despite its very low quality, indicates that clopidogrel or DAPT probably increases the risk of perioperative bleeding. However, the extent and clinical significance of this risk remain poorly defined. Data regarding the perioperative risks associated with aspirin monotherapy are also limited, but there is a trend indicating increased risk of bleeding or need for transfusion.

Studies on VKAs suggest an increased bleeding risk, particularly when anticoagulation is continued post-transplant. Perioperative INR values, suggestive of TTR, were not consistently reported in the studies, and therefore we cannot make any recommendation about safe threshold values or reversal agents. Additionally, data on DOACs is too limited to draw definitive conclusions and therefore cannot be supported in deceased kidney transplantation.

### What do existing guidelines say?

The KDIGO 2020 guidelines [3] state that KT candidates should not be excluded from transplantation due to anticoagulation or antiplatelet medication. Single antiplatelet agents (e.g. aspirin) can be continued while on the deceased donor waiting list. Transplant should be delayed for patients on DAPT (e.g. aspirin + clopidogrel) if the risks outweigh the benefits. KDIGO also recommends stopping antiplatelet agents (except for aspirin) 5 days before elective live donor transplants. Warfarin should also be stopped 5 days prior for elective transplants, with bridging as needed. DOACs (e.g. apixaban) are discouraged unless there is perioperative expertise and reversal options and therefore, most patients should be switched to alternatives (e.g. warfarin) if needed.

Current European Society of Cardiology (ESC) guidance on perioperative antithrombotic management in non-cardiac surgery provides several principles that may be applicable to KT surgery, classified as a moderate cardiac and high bleeding risk surgery [75]. In patients with a prior PCI, continuation of low-dose aspirin throughout the perioperative period is recommended when the surgical bleeding risk is acceptable (Class I). For candidates without a history of PCI, aspirin discontinuation at least 3 days before transplantation may be considered when bleeding risk clearly exceeds ischemic risk (Class IIb), given the substantial intraoperative and post-operative bleeding burden associated with transplant surgery. Elective procedures should ideally be postponed for at least 6 months after PCI (Class I). Once the mandatory DAPT period has passed, P2Y_12_ inhibitors should be withheld according to their pharmacodynamic profiles (3–5 days for ticagrelor, 5 days for clopidogrel and 7 days for prasugrel) prior to surgery (Class I). For patients on anticoagulants who are at high thrombotic risk, perioperative bridging may be considered, otherwise bridging is not recommended (Class IIb).

According to the CHEST perioperative guidelines published in 2022 [76], KT is acknowledged as a high-bleed-risk surgery (quoted as >2% 30-day risk of major bleeding). This guideline does not also recommend heparin bridging for patients on oral anticoagulant treatment before surgery depending on the indication. However, there are no further data offered to clarify additional risk; this guideline only covers elective surgery, and it is unknown whether these recommendations can be safely extrapolated to KT surgery (both elective and emergency).

These recommendations are summarized in the Table 3.

**Table 3:**
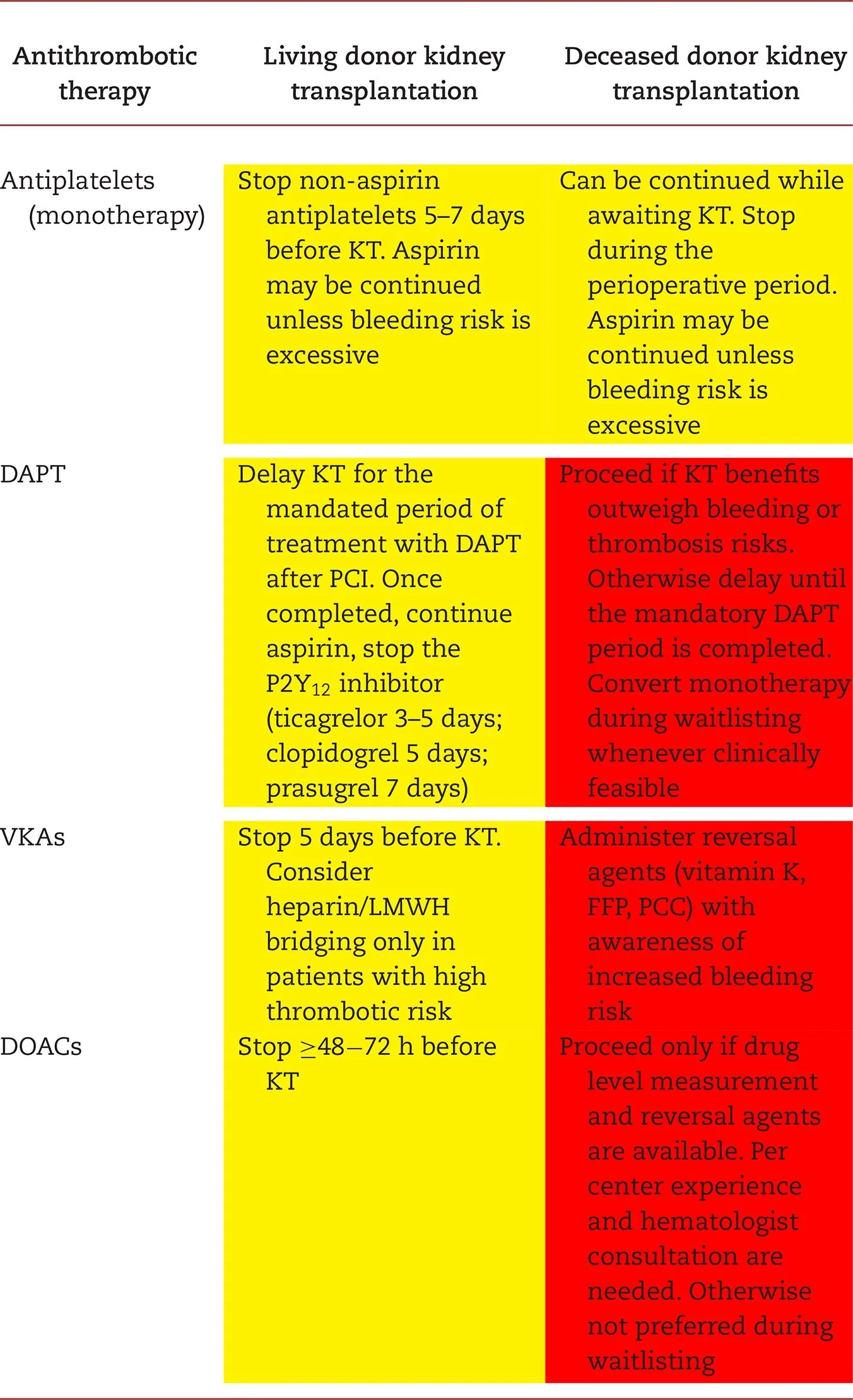
Summary of recommendations for pre-transplant antithrombotic use and management before surgery.^a,b^

FFP, fresh frozen plasma; PCC, prothrombin complex concentrate. Note: **bold** for conditional cases, *italics* for high-risk situations.

References:

a Chadban SJ, Ahn C, Axelrod DA*. et al*. KDIGO Clinical Practice Guideline on the Evaluation and Management of Candidates for Kidney Transplantation. *Transplantation* 2020;104(4S1 Suppl 1):S11–103; doi: 10.1097/TP.0000000000003136.

b Halvorsen S, Mehilli J, Cassese S*. et al.*; ESC Scientific Document Group. 2022 ESC Guidelines on cardiovascular assessment and management of patients undergoing non-cardiac surgery. *Eur Heart J* 2022;43:3826–924; doi: 10.1093/eurheartj/ehac270.

### Clinical practice points

- The decision to continue, interrupt or modify antithrombotic therapy prior to transplantation should be individualized based on the expected waiting time for transplantation, availability of reversal agents and the center’s experience.
- Clinicians should inform patients about the potential risk of bleeding with any antithrombotic therapy used in deceased donor KT, which is significant for clopidogrel, DAPT and anticoagulants.
- DOAC use in waitlisted patients is not recommended, given the limited evidence regarding their safety in transplant surgery.

## QUESTION 2: SHOULD KT RECIPIENTS RECEIVE PROPHYLAXIS FOR VENOUS THROMBOSIS AFTER SURGERY?

Authors: Erol Demir, Luuk Hilbrands, Artemios Karagiannidis

### Background and rationale

Venous thrombosis is a significant risk factor for early allograft loss and contributes to perioperative morbidity in KT recipients. To prevent such adverse outcomes in the post-operative period, clinicians use thromboprophylaxis regimens, but the exact protocols, timing and eligibility criteria differ between centers, because no universal guidelines are available, and this decision is mostly based on individual center experience. Therefore, we aimed to evaluate the efficacy of thromboprophylaxis post KT to prevent venous thrombosis and pulmonary embolism and summarize the exact details of such regimens. Specifically, we reviewed evidence regarding the optimal timing, appropriate dosing and overall effectiveness of thromboprophylaxis, as well as its potential risks.

### Summary of evidence

(Supplement | Study selection flow diagram for Question 2, see Supplementary data, Fig. S2)

(Supplement | Summary evidence table for Question 2, see Supplementary data, Table S11)

Given the highly diverse outcomes, definitions and variable rates of complications, we opted again to review evidence categorized by the type of anticoagulant medication. Our search identified 10 original studies (2 randomized prospective clinical trials, 1 prospective cohort study and 7 retrospective cohort studies). The studies were heterogeneous in design, population and outcomes, with overall high risk of bias and low certainty of evidence.

#### Antithrombotic prophylaxis with unfractionated heparin or low-molecular weight heparin

In a single-center prospective study [77], patients were randomized to receive either no heparin or subcutaneous (SC) unfractionated heparin for 7 days or until the patient achieved fully mobility. Within 18 days post-operatively, thromboembolic events (graft vein thromboses, arteriovenous fistula thromboses, deep vein thrombosis and pulmonary embolism) were reported only in the no-heparin group. Although the study was small and not powered for statistical significance, the findings suggest a protective effect of post-operative heparin prophylaxis without significant increases in bleeding or wound complications.

In a prospective cohort study [78], patients without hypercoagulable states (normal coagulation screening tests, absence of a clinical history of hypercoagulability) received dalteparin immediately after surgery and then daily throughout their hospitalization. Only one case of venous thrombosis was reported, and no bleeding complications were reported. The lack of a control group limits the assessment of the true efficacy of this prophylactic approach.

A third prospective study randomized living donor KT recipients to receive either unfractionated heparin for 1 week, tinzaparin for 1  week or no prophylaxis [79]. No thromboembolic events were reported in any group, limiting the assessment of the potential benefit of prophylaxis. However, decreases in post-operative hemoglobin levels were higher in the anticoagulated groups (with higher decrease in the unfractionated heparin group compared with the LMWH group), highlighting potential associations between anticoagulation and mild hemoglobin reduction.

In a retrospective cohort study [80], patients who received peri- or post-operative enoxaparin were compared with those who did not; prophylaxis was administered in the first group for a mean duration of 10.4 days. One venous graft thrombosis was reported in the no-prophylaxis group, but there were no detailed data on the incidence of deep venous thrombosis and pulmonary embolism. In a second retrospective study [81] comparing various prophylactic heparin regimens (heparin 5000 IU SC twice daily, heparin 5000–7500 IU SC thrice daily, dalteparin 5000 IU SC daily) with no prophylaxis, thrombotic events during the first post-operative week were uncommon in both groups, with slightly fewer events among those receiving prophylaxis. Importantly, bleeding risks were comparable between groups in both studies, a finding that further supports the safety of both unfractionated and LMWH prophylaxis in KT recipients.

In a large retrospective cohort study [82], deep vein thrombosis (excluding graft thrombosis) occurred in almost 5% of KT recipients within the first 3 months after transplantation. Antithrombotic prophylaxis, defined as at least one dose of SC unfractionated heparin 5000 IU administered between 24 h before and 48 h after transplantation, did not reduce the risk of deep vein thrombosis. However, the retrospective design and the lack of precise information on prophylaxis duration preclude definitive conclusions.

Finally, van den Berg *et al.* [25] showed that post-operative continuous administration of unfractionated heparin (12 000 IU/day for 5 days) resulted in a higher bleeding risk than prophylactic SC nadroparin (2850 IU/daily), underscoring the importance of avoiding unnecessarily aggressive anticoagulation regimens.

#### Antithrombotic prophylaxis with aspirin

A retrospective study demonstrated a lower incidence of renal vein thrombosis after prescribing low-dose aspirin (75 mg/day), initiated immediately before and continued for 1 month after transplantation. However, confounding factors (e.g. patient selection, improved care over time between cohorts) limit the strength of this observation [53]. Importantly, patients in this study did not receive heparin prophylaxis. Another retrospective study found no cases of primary allograft thrombosis among patients receiving higher-dose aspirin (150 mg/day) for the first three post-operative months combined with a short course of peri-operative heparin (5000 IU pre-transplantation and 5000 IU twice daily SC for 5  days post-transplantation) [83]. Cases of graft thrombosis were observed only in the untreated control group, with an incidence higher than what is currently reported in patients receiving LMWH prophylaxis.

In a retrospective single-center study [84], the duration of aspirin treatment (100 mg/day for 5 days or >6 weeks post-operatively) did not appear to affect VTE incidence in the first 6 weeks following KT. In this study, patients could receive concurrent heparin prophylaxis, but the exact proportion of patients who received heparin was not reported.

### Translation of the evidence into clinical practice

While LMWH prophylaxis for venous thrombosis is routinely used in many KT centers, solid support for this practice is lacking. In patients without specific risk factors for venous thrombosis, the absolute incidence of thromboembolic events after kidney transplantation is low despite an increased relative risk.

Of the three small prospective studies, one did not include a control group and in another one there were no cases of deep vein thrombosis in the control group, which precludes conclusions about efficacy. The retrospective studies included more patients but were low-quality evidence. Nevertheless, there was not a substantially increased risk of bleeding to be associated with antithrombotic prophylaxis.

There is no strong evidence on a potential additional beneficial effect of aspirin in patients receiving LMWH prophylaxis in whom the rate of venous allograft thrombosis is already low.

Taken together, based on the known increased incidence of VTE after KT, the proven efficacy in other types of pelvic and vascular surgery, and the favorable safety profile of prophylactic doses of LMWH in KT recipients, current practice appears to be justified.

### What do existing guidelines say?

The European Association of Urology Guidelines on Renal Transplantation (2024) [85] provides the following recommendation, graded as weak: “Do not routinely give post-operative prophylactic unfractionated or low-molecular weight heparin to low-risk living donor transplant recipients.” The recommendation is based on a single study involving 75 live donor kidney recipients [79], as discussed earlier.

The following guidelines do not give recommendations on routine prophylaxis for venous thrombosis after surgery:

- Post-operative care in the KT recipient of the UK Kidney Association (2017) [86]
- KDIGO clinical practice guideline for the care of KT recipients (2009) [87]

### Clinical practice points

- Based on the efficacy of antithrombotic prophylaxis in other surgical settings, antithrombotic prophylaxis after kidney transplantation can be justified.
- Prophylaxis with unfractionated heparin or LMWH is not associated with an increased risk of bleeding.

## QUESTION 3: CAN WE USE DOACs IN KT RECIPIENTS IN TERMS OF SAFETY (DOSING, DRUG–DRUG INTERACTIONS, ADVERSE EVENTS)?

Authors: Ivana Dedinská, Annelies de Weerd, Artemios Karagiannidis, Matej Samoš

### Background and rationale

The long-term use of oral anticoagulation in KT recipients with atrial fibrillation/atrial flutter (AF/AFlu) and VTE is crucial to prevent thromboembolic events (stroke or systemic embolism in those with AF/AFlu and repeated pulmonary embolism and/or deep vein thrombosis in those with VTE). However, there are no specific recommendations and guidelines on therapeutic strategies in these patients. Traditional oral anticoagulants like VKAs, such as warfarin, present challenges due to narrow therapeutic indices, the need for frequent monitoring, risk of calciphylaxis and potential interactions with immunosuppressants or some other drugs, such as antibiotics. DOACs, including apixaban, rivaroxaban, dabigatran and edoxaban, have an alternative mechanism of action and have become first-line treatment for AF and the prevention of thromboembolism in the general population (except if mitral stenosis or mechanical heart valve) [88]. DOACs offer a potentially safer alternative to VKAs due to their predictable pharmacokinetics and reduced need for monitoring. However, although there is no clear contraindication for DOACs therapy in KT recipients, their use requires careful consideration due to several factors:

- Kidney function: KT recipients often have impaired graft function, which can affect DOACs clearance and increase the risk of bleeding
- Drug–drug interactions: DOACs can interact with immunosuppressants, particularly CNIs, tacrolimus and cyclosporine, potentially altering their pharmacokinetic and pharmacodynamic profiles
- Bleeding risk: it associated with DOACs remains a critical concern due to altered kidney function, potential drug–drug interactions with immunosuppressants, particularly during invasive procedures
- Limited data: compared with their use in other populations, evidence on DOAC use in KT recipients is limited, especially regarding long-term safety and optimal dosing strategies

Therefore, we aimed at evaluating the efficacy of DOACs on KT recipients and reviewing evidence regarding the appropriate dosing, exact drug–drug interactions, overall effectiveness and adverse effects.

### Summary of evidence

(Supplement | Study selection flow diagram for Question 3, see Supplementary data, Fig. S3)

(Supplement | Summary evidence table for Question 3, see Supplementary data, Table S12)

The evidence regarding DOAC use in KT recipients is heterogeneous and generally of low certainty, making definitive recommendations challenging. Our search identified 15 studies (10 retrospective studies, 1 open-label drug–drug interaction study and 4 prospective studies).

#### DOAC choice and dosage

Apixaban studies suggest lower major bleeding rates compared with warfarin, although these findings are not consistently replicated across all studies and are often based on small sample sizes and/or retrospective designs [89–94]. The studies show varying results with rivaroxaban, with some showing no increased bleeding risk compared with warfarin [95, 96], while other reports a higher incidence of minor bleeding [97]. Data on dabigatran and edoxaban are limited, preventing robust conclusions about their safety and efficacy in KT recipients.

#### Bleeding and thrombotic events

There are no randomized controlled trials comparing DOAC versus VKA in KT recipients. We identified five studies comparing DOACs and VKAs in terms of bleeding and thromboembolic events [89, 90, 95, 96, 98] (Table 4). Four were retrospective studies, only one of which was multicenter prospective follow-up study [90]. With regards to safety, Leon *et al*. reported significantly less bleeding events with DOACs [98], whereas three studies a non-significant trend to less bleeding [89, 95, 96] and one study had more bleeding events [90].

**Table 4:** Studies for safety of DOACs comparing with warfarin in KT recipients.

Study (author, year)	Country	DOAC	VKA	Characteristics	Follow-up	Bleeding event	Thromboembolic event	Remark
Bixby *et al.*, 2020^a^	USA	*N* = 99 (apixaban, *n* = 60; dabigatran, *n* = 6; rivaroxaban, *n* = 33)	Warfarin, *N* = 98	Median 6.5 years posttransplant	11 vs 13 months	Major bleeding: 9% vs 21%	VTE: 2% vs 3%; stroke: 1% vs 5%	eGFR <30 mL/min: 7% vs 15%
Leon *et al.*, 2020^b^	France	*N* = 52 (apixaban, *n* = 36; dabigatran, *n* = 1; rivaroxaban, *n* = 15)	*N* = 50 (warfarin, *n* = 20, fluindione, *n* = 30)	87 vs 65 months posttransplant	14 vs 22 months	Bleeding: 13% vs 42% (*P* = .002)	PE or DVT: 0% vs 8%	No DOAC if mechanical valve
Zaitoun *et al.*, 2021^c^	Saudi Arabia	*N* = 8 (apixaban, *n* = 1; dabigatran, *n* = 1; rivaroxaban, *n* = 6)	Warfarin, *N* = 8 (matched)	Not specified	Unclear	Bleeding: 0% vs 38%	0% vs 0%	Limited sample size
Firth *et al.*, 2023^d^	USA	*N* = 208 (apixaban, *n* = 189; rivaroxaban, *n* = 19)	Warfarin, *N* = 320	DOAC more recent (post-2016)	449 vs 1098 days	Major bleeding: 5% vs 8%	VTE: 36% vs 39%; ischemic stroke 5% vs 5%	eGFR not reported
Santoro *et al.*, 2024^e^	Italy	*N* = 66 (apixaban, *n* = 32; edoxaban, *n* = 17; dabigatran, *n* = 3; rivaroxaban, *n* = 14)	Warfarin, *N* = 50	Median 145 vs 90 months posttransplant	20 months	Major bleeding: 6% vs 4%	0% vs 0%	AF indication: 71% vs 56%

DVT, deep vein thrombosis; PE, pulmonary embolism.

References:

a Bixby AL, Shaikh SA, Naik AS*. et al*. Safety and efficacy of directE: Venous Thromboembolismicoagulants, lar assessment and management of patients undergoing non-cardiac surgery. erwise no*Transpl Int* 2020;33:740–51; doi:10.1111/tri.13599

b Leon J, Sabbah L, Aubert O*. et al*. Efficacy and Safety of direct oral anticoagulants in kidney transplantation: a single-center pilot experience. *Transplantation* 2020;104:2625–31; doi: 10.1097/TP.0000000000003168.

c Zaitoun MF, Sheikh ME, Faifi ASA. *et al*. The use of non–vitamin K antagonist oral anticoagulants in post-kidney transplantation, single-center experience. *Transplant Proc* 2021;53:2918–22; doi:10.1016/j.transproceed.2021.09.042.

d Firth C, Shamoun F, Apolinario M. *et al*. Safety and mortality outcomes for direct oral anticoagulants in renal transplant recipients. *PLoS ONE* 2023;18:e0285412; doi:10.1371/journal.pone.0285412.

e Santoro F, Casanova A, Simone S*. et al*. Immunosuppressive therapy and oral anticoagulation in kidney transplant recipients: direct oral anticoagulants versus vitamin-K antagonists. *Eur J Intern Med* 2024;119:71–7; doi:10.1016/j.ejim.2023.08.003.

Regarding efficacy, in two studies there were no thromboembolic events, two studies reported non-significantly less and one study non-significantly more thromboembolic events [89, 90, 95, 96, 98]. A limitation of all studies was the lack of information concerning the selection of anticoagulation, which was at the discretion of the treating physician. The duration of the study period additionally impacted outcomes, as most studies had shorter follow-up durations for DOACs. Four studies explicitly excluded patients with eGFR <30 mL/min [90, 96, 98, 99], one study did not report kidney function [95] and one study described that 7% of DOAC and 15% of warfarin treated patients had eGFR <30 mL/min [89].

Do *et al*. conducted a single-center retrospective study of 52 adult KT recipients receiving apixaban, reporting two major bleeding events (3.8%) along with two thromboembolic events (3.8%) over 12 months. [100] The lack of a comparison group makes it difficult to interpret the outcomes.

#### Drug–drug interactions

Several studies highlight potential interactions between DOACs and immunosuppressants (particularly CNIs), leading to altered drug levels [91, 92, 101, 102]. In contrast Camporese *et al.* [99] conducted a prospective study on eight KT recipients receiving tacrolimus (with or without everolimus) and rivaroxaban for VTE or non-valvular AF. Over a 2-week period, no major bleeding or thromboembolic events occurred, and tacrolimus/everolimus through levels remained stable, suggesting no significant drug–drug interactions. A single-center retrospective study on 31 KT recipients receiving DOACs (mostly apixaban) showed that 94% of measured DOAC levels were within the expected therapeutic range [94]. In another , Zaitoun *et al.* [96] found no significant changes in CNI trough-to-dose ratios in patients receiving DOACs.

Overall, drug–drug interactions are often not clinically significant in terms of adverse events, but further research is needed to fully understand the impact and to determine whether therapeutic drug monitoring is routinely beneficial.

#### Other outcomes

The impact of DOACs on graft function (eGFR) is unclear, with some studies reporting no significant effect, while others show possible improvement with rivaroxaban [91, 92]. Furthermore, mortality rates are not consistently compared between patients on DOACs versus those on warfarin.

### Translation of the evidence into clinical practice

Because of the limitations of available evidence, particularly the lack of randomized controlled trials or large-scale propensity score–matched studies, the decision to use DOACs in KT recipients should be individualized, carefully weighing the potential benefits against the risks. However, current findings from existing studies support the use of DOACs based on their effective and safety profile.

It should be noted that there is still a paucity of data in patients with an eGFR <30 mL/min/1.73 m², requiring extra caution in this group. The most clinical experience to date has been obtained with apixaban. The choice of DOAC and dosage should be guided by the patient’s clinical characteristics and graft function. Due to the complexity of this patient population who often have altered pharmacokinetics, multiple comorbidities, and concomitant immunosuppressive therapies, management of anticoagulation should involve interdisciplinary collaboration, especially between transplant specialists, cardiologists or vascular specialists and clinical pharmacists to optimize safety and therapeutic efficacy.

### What do existing guidelines say?

There is no universal or specific guideline that directly addresses the use of DOACs in KT recipients.

### Clinical practice points

- The choice of anticoagulant (DOAC vs VKA) should be individualized; although existing studies suggest that DOACs have a favorable efficacy and safety profile, the evidence in KT recipients remains insufficient to recommend one class over the other.
- Close monitoring of kidney function is recommended for KT recipients on DOACs, given the need for dose modification.
- The use of DOACs in KT recipients with GFR <30 mL/min/1.73 m² should be approached with caution.
- DOACs may interact with CNIs, though most interactions appear clinically insignificant.

## QUESTION 4: IS THERE A NEED FOR INTERRUPTION OF ANTIPLATELET THERAPY BEFORE GRAFT BIOPSY?

Authors: Erol Demir, Artemios Karagiannidis, Andreas Kousios

### Background and rationale

Bleeding is a significant complication of kidney graft biopsies, with the potential to impact graft and patient outcomes. We aimed at investigating the necessity and safety of interrupting antiplatelet therapy prior to kidney graft biopsy to mitigate bleeding risks. Specifically, we reviewed current evidence on the clinical rationale, patient-specific considerations and potential risks associated with therapy interruption, as well as strategies to balance bleeding and thrombotic complications.

### Summary of evidence

(Supplement | Study selection flow diagram for Question 4, see Supplementary data, Fig. S4)

(Supplement | Summary evidence table for Question 4, see Supplementary data, Table S13)

Our search identified seven original studies, all of which were of retrospective nature.

Robertson *et al*. [53] investigated bleeding complications in KT recipients receiving either 75 mg or 150 mg of aspirin daily for at least 1 month to prevent renal vein thrombosis. Post-biopsy bleeding occurred in a small proportion of patients, representing 1.6% of all biopsies, a finding consistent with rates reported by prior studies (0.3%–12.6%). However, the study’s interpretability is limited by the lack of clarity about whether aspirin was continued at the time of graft biopsy; this undermines the ability to accurately contextualize the bleeding risk.

Murphy *et al*. [83] evaluated deceased donor KT recipients treated with aspirin 150 mg daily for the first 3 months post-transplant and compared them to a historical control group without aspirin. All patients underwent protocol needle-core biopsies at 1 week and 12 months after transplantation. Rates of macroscopic hematuria following graft biopsy were similar between groups; however, the lack of information on how many biopsies were performed while patients were actively taking aspirin is a strong limitation.

A single-center, 5-year retrospective study of >2500 renal transplant biopsies examined major complications and associated risk factors. [103] Anticoagulants and antiplatelets (warfarin, clopidogrel, and aspirin low- and full-dose), were withheld for 7 days, unless an urgent biopsy was required. Major complications within 14 days post-biopsy were uncommon. Several clinical parameters, i.e. older age, lower platelet count, higher blood urea nitrogen (BUN), deceased donor transplant, prior renal transplant, and use of heparin, clopidogrel or warfarin were recognized as significant risk factors. Cutoff values for increased complication risk included age ≥51 years, creatinine ≥2.23 mg/dL, BUN ≥40 mg/dL, platelet count ≤173 000/μL and hematocrit ≤31%. In contrast, aspirin use was not associated with higher risk for biopsy complications.

In another single-center retrospective study of >6000 renal allograft biopsies, Baffour *et al.* [104] examined the association between aspirin use and post-biopsy bleeding event. All ultrasound-guided biopsies were performed with INR <1.5 and platelet counts >50 × 10⁹/L, using ≥18-gauge needles, with three cores obtained per patient. Most of the performed biopsies were protocol biopsies (81.3%). Aspirin doses were low (81 mg/day) or high (325 mg/day). Overall bleeding rates were low; bleeding occurred in 1.7% of patients who took aspirin within 3 days of biopsy, 0.3% in those who took it 4–7 days before, and 0.3% in those who took it 8–10 days prior, compared with 0.7% in non-users. Importantly, there was no association between aspirin use and bleeding risk, except in patients on high-dose aspirin taken within 3 days of biopsy, who presented higher risk of both minor and major bleeding.

Li *et al*. [105] developed a standardized protocol for percutaneous kidney graft biopsies to reduce bleeding complications by optimizing blood pressure, platelet function and anticoagulation management. Implementation of this protocol led to a notable reduction in overall and major hemorrhage rates by almost 50% and 70%, respectively. The approach included real-time ultrasound guidance and discontinuation of aspirin and clopidogrel for 5–7 and 7 days, respectively, before elective biopsies. While effective, the protocol’s applicability is limited by its single-center setting and variability of patients’ characteristics. Kuiper *et al.* [106] assessed the predictive value of hemostasis tests and clinical factors for bleeding complications in patients undergoing kidney graft biopsy, 26 (17%) of whom were on aspirin. Aspirin was discontinued in all but one patient 5 days before the biopsy. Although overall bleeding rates were low, with only one major complication requiring transfusion, prior use of aspirin (discontinued 5 days before biopsy) was linked to higher bleeding risk. The study concluded that routine hemostasis testing is unreliable for predicting post-biopsy bleeding, with aspirin use being the primary risk factor.

Lastly, Mattiazzi *et al*. [107] compared outcomes of ultrasound-guided kidney graft biopsies performed by either interventional radiologists or transplant nephrologists. Antiplatelet or anticoagulant drugs were used by 40% and 4% of the patients, respectively, and was routinely discontinued at least 7 days before the biopsy. Complication rates were similar between the two groups, with uncontrolled blood pressure and prior antiplatelet use linked to higher risk of post-biopsy complications. These findings underscore the need for individualized management, particularly in optimizing blood pressure control and timing of antithrombotic interruption to balance bleeding and thrombotic risks in KT recipients.

### Translation of the evidence into clinical practice

Overall, the current literature suggests that major bleeding following allograft biopsy remains rare, even in patients with recent aspirin exposure. However, due to limited evidence and the retrospective nature of existing studies, no clear consensus exists regarding peri-procedural management of antiplatelet drugs in KT recipients. Most transplant centers likely adopt a case-by-case approach, performing individualized risk-benefit analyses, particularly in patients with recent cardiovascular interventions such as coronary stents. Given the recognized high bleeding risk associated with percutaneous biopsy, these decisions must balance thrombotic risk against the potential for hemorrhagic complications.

### What do existing guidelines say?

There is no universal or specific guideline that directly addresses the interruption of antiplatelet therapy prior to graft biopsy in KT recipients.

### Clinical practice points

- Consider patient-specific factors, such as age, platelet count, graft function, blood pressure and indication for antithrombotic therapy when deciding on the necessity of interrupting antiplatelet therapy.
- Low-dose ASA (≤150 mg/day) generally does not increase bleeding risk if discontinued at least 3–7 days before biopsy; continued ASA use within 3 days prior to biopsy may be associated with a higher risk of bleeding; discontinuing high-dose ASA (>300 mg) at least 5–7 days prior to biopsy should be considered to avoid bleeding.
- Discontinuation of clopidogrel at least 7 days prior to biopsy could be considered.

## LIMITATIONS OF INCLUDED STUDIES

The studies included in our review have several important limitations. Firstly, most of the available evidence derives from retrospective single-center cohort studies or small case series, with only a handful of relevant prospective or randomized studies. This substantially increases the risk of selection bias and residual confounding. In many cases, the choice of antithrombotic therapy was at the discretion of the treating clinician, introducing potential indication bias. In addition, sample sizes were generally small or moderate, and outcomes of interest are relatively infrequent; as a result, many effect estimates are imprecise with wide confidence intervals, with several analyses underpowered to detect significant differences. Although multivariable models or propensity methods could have helped adjust for confounding, this was performed only by the minority of studies and even then, adjustment was often limited (e.g. incomplete data on cardiovascular risk profile, prior bleeding, concomitant antiplatelet use, immunosuppressive regimens or center-specific surgical practices).

Additionally, there was substantial heterogeneity across studies with populations varying widely with regards to type of transplant (living vs deceased donor), baseline thrombotic/bleeding risk, comorbidities, graft function and indication for antithrombotic therapy (e.g. AF, VTE, stent, primary prophylaxis, etc.). Interventions also differed significantly between studies in terms of drug classes, doses, timing of discontinuation/restart, bridging approach and concomitant therapies. This heterogeneity limits comparability and precludes definitive conclusions.

Outcome definitions and reporting practices were equally inconsistent. Bleeding and thrombotic events were not uniformly defined and follow-up windows ranged from immediate in-hospital post-operative period to several months. Some studies reported only transfusion requirements, others only reoperation rates, and many did not distinguish between minor and major bleeding. Perioperative INR levels, platelet counts, biopsy technique and use of reversal agents were often missing.

Taken together, these limitations result in the overall low certainty of evidence and high risk of bias of these studies. Therefore, our conclusions are framed as clinical practice points rather than strong, graded, guideline recommendations.

## SUGGESTIONS FOR FUTURE RESEARCH

Rigorous, large-scale studies with standardized treatment protocols are needed to clarify the effects of pre-transplant antithrombotic therapy on transplant outcomes. Specifically, research should focus on the use of point-of-care platelet function tests to assess actual antiplatelet activity prior to deceased donor KT surgery. Pre-operative or intraoperative coagulation measured by thromboelastography can offer real-time insight into global hemostasis and help guide transfusion and perioperative antithrombotic decisions [108]. Given the increasing use of DOACs, especially apixaban, in the ESKD population, bleeding risk stratification tools and the role of DOAC reversal agents in KT candidates warrant further investigation. Additionally, large, prospective or propensity score–matched cohort studies comparing DOACs and warfarin in KT recipients could help determine optimal dosing and safety, and their impact on graft and patient outcomes. Research should also address patient-specific factors influencing bleeding risk in KT recipients on DOACs to improve risk stratification. Data on the use of betrixaban, a new DOAC, in CKD, and particularly in KT recipients, is currently lacking. Given its low renal excretion, this agent may offer a preferable profile [109]. Finally, FXI inhibitors, due to their potential to provide safe anticoagulation irrespective to kidney function, represent a promising research avenue in both transplant candidates and recipients [110, 111].

## Supplementary Material

gfag024_Supplemental_File

## Data Availability

No new data were generated or analyzed in support of this research.

## References

[1] Matsushita K, Coresh J, Sang Y et al. Estimated glomerular filtration rate and albuminuria for prediction of cardiovascular outcomes: a collaborative meta-analysis of individual participant data. Lancet Diabetes Endocrinol 2015;3:514–25. 10.1016/S2213-8587(15)00040-6 26028594 PMC4594193

[2] Kumar S, Lim E, Covic A et al. Anticoagulation in concomitant chronic kidney disease and atrial fibrillation. J Am Coll Cardiol 2019;74:2204–15. 10.1016/j.jacc.2019.08.1031 31648714

[3] Chadban SJ, Ahn C, Axelrod DA et al. KDIGO Clinical Practice Guideline on the Evaluation and Management of Candidates for Kidney Transplantation. Transplantation 2020;104:S11–103. 10.1097/TP.0000000000003136 32301874

[4] Lentine KL, Brennan DC, Schnitzler MA. Incidence and predictors of myocardial infarction after kidney transplantation. J Am Soc Nephrol 2005;16:496–506. 10.1681/ASN.2004070580 15615820

[5] Lentine KL, Schnitzler MA, Abbott KC et al. Incidence, predictors, and associated outcomes of atrial fibrillation after kidney transplantation. Clin J Am Soc Nephrol 2006;1:288–96. 10.2215/CJN.00920805 17699219

[6] Lentine KL, Rey LAR, Kolli S et al. Variations in the risk for cerebrovascular events after kidney transplant compared with experience on the waiting list and after graft failure. Clin J Am Soc Nephrol 2008;3:1090–101. 10.2215/CJN.03080707 18385393 PMC2440268

[7] Erlandsson H, Qureshi AR, Ripsweden J et al. Scoring of medial arterial calcification predicts cardiovascular events and mortality after kidney transplantation. J Intern Med 2022;291:813–23. 10.1111/joim.13459 35112417 PMC9306575

[8] Lam NN, Garg AX, Knoll GA et al. Venous thromboembolism and the risk of death and graft loss in kidney transplant recipients. Am J Nephrol 2017;46:343–54. 10.1159/000480304 29024935

[9] Baaten CCFMJ, Schröer JR, Floege J et al. Platelet abnormalities in CKD and their implications for antiplatelet therapy. Clin J Am Soc Nephrol 2022;17:155–70. 10.2215/CJN.04100321 34750169 PMC8763166

[10] Lutz J, Menke J, Sollinger D et al. Haemostasis in chronic kidney disease. Nephrol Dial Transplant 2014;29:29–40. 10.1093/ndt/gft209 24132242

[11] D’Apolito M, Du X, Pisanelli D et al. Urea-induced ROS cause endothelial dysfunction in chronic renal failure. Atherosclerosis 2015;239:393–400. 10.1016/j.atherosclerosis.2015.01.034 25682038 PMC4361277

[12] Thekkedath UR, Chirananthavat T, Leypoldt JK et al. Elevated fibrinogen fragment levels in uremic plasma inhibit platelet function and expression of glycoprotein IIb-IIIa. Am J Hematol 2006;81:915–26. 10.1002/ajh.20720 16917914

[13] Alves De Sá Siqueira M, Brunini TMC, Rodrigues Pereira N et al. Increased nitric oxide production in platelets from severe chronic renal failure patients. Can J Physiol Pharmacol 2011;89:97–102. 10.1139/Y10-111 21326340

[14] Remuzzi G, Benigni A, Dodesini P et al. Reduced platelet thromboxane formation in uremia. Evidence for a functional cyclooxygenase defect. J Clin Invest 1983;71:762–8. 10.1172/JCI110824 6298281 PMC436927

[15] Weisel JW, Litvinov RI. Red blood cells: the forgotten player in hemostasis and thrombosis. J Thromb Haemost 2019;17:271–82. 10.1111/jth.14360 30618125 PMC6932746

[16] Ocak G, Lijfering WM, Verduijn M et al. Risk of venous thrombosis in patients with chronic kidney disease: identification of high-risk groups. J Thromb Haemost 2013;11:627–33. 10.1111/jth.12141 23433091

[17] Baaten CCFMJ, Sternkopf M, Henning T et al. Platelet function in CKD: a systematic review and meta-analysis. J Am Soc Nephrol 2021;32:1583–98. 10.1681/ASN.2020101440 33941607 PMC8425648

[18] Van Den Berg TAJ, Nieuwenhuijs-Moeke GJ, Lisman T et al. Pathophysiological changes in the hemostatic system and antithrombotic management in kidney transplant recipients. Transplantation 2023;107:1248–57. 10.1097/TP.0000000000004452 36529881 PMC10205120

[19] Sarnak MJ, Amann K, Bangalore S et al. Chronic kidney disease and coronary artery disease. J Am Coll Cardiol 2019;74:1823–38. 10.1016/j.jacc.2019.08.1017 31582143

[20] Kaw D, Malhotra D. Hematology: issues in the dialysis patient: platelet dysfunction and end-stage renal disease. Semin Dial 2006;19:317–22. 10.1111/j.1525-139X.2006.00179.x 16893410

[21] Skinner SC, Derebail VK, Poulton CJ et al. Hemodialysis-related complement and contact pathway activation and cardiovascular risk: a narrative review. Kidney Med 2021;3:607–18. 10.1016/j.xkme.2021.04.006 34401728 PMC8350825

[22] Sloand JA, Sloand EM. Studies on platelet membrane glycoproteins and platelet function during hemodialysis. J Am Soc Nephrol 1997;8:799–803. 10.1681/ASN.V85799 9176850

[23] Daugirdas JT, Bernardo AA. Hemodialysis effect on platelet count and function and hemodialysis-associated thrombocytopenia. Kidney Int 2012;82:147–57. 10.1038/ki.2012.130 22592187

[24] Nieuwenhuijs-Moeke GJ, Van Den Berg TAJ, Bakker SJL et al. Preemptively and non-preemptively transplanted patients show a comparable hypercoagulable state prior to kidney transplantation compared to living kidney donors. PLoS One 2018;13:e0200537. 10.1371/journal.pone.0200537 30011293 PMC6047796

[25] van den Berg TAJ, Minnee RC, Lisman T et al. Perioperative antithrombotic therapy does not increase the incidence of early postoperative thromboembolic complications and bleeding in kidney transplantation—a retrospective study. Transpl Int 2019;32:418–30. 10.1111/tri.13387 30536448 PMC6850661

[26] Hachem LD, Ghanekar A, Selzner M et al. Postoperative surgical-site hemorrhage after kidney transplantation: incidence, risk factors, and outcomes. Transpl Int 2017;30:474–83. 10.1111/tri.12926 28120465

[27] Willicombe M, Roberts DJ. Transfusion-induced HLA sensitization in wait-list patients and kidney transplant recipients. Kidney Int 2024;106:795–805. 10.1016/j.kint.2024.07.030 39181398

[28] Dongol RM, Pahwa M, Adhikari S et al. Use of fibrin sealant in vein anastomosis in renal transplantation and its effect in preventing blood loss and postoperative peri-graft collection. Int Urol Nephrol 2024;57:769–73. 10.1007/s11255-024-04247-7 39455519

[29] Morales JM, Serrano M, Martinez-Flores JA et al. Antiphospholipid syndrome and renal allograft thrombosis. Transplantation 2019;103:481–6. 10.1097/TP.0000000000002510 30376553

[30] Kuo HH, Fan R, Dvorina N et al. Platelets in early antibody-mediated rejection of renal transplants. J Am Soc Nephrol 2015;26:855–63. 10.1681/ASN.2013121289 25145937 PMC4378099

[31] Parajuli S, Lockridge JB, Langewisch ED et al. Hypercoagulability in kidney transplant recipients. Transplantation 2016;100:719–26. 10.1097/TP.0000000000000887 26413991

[32] Varian FL, Parker WAE, Fotheringham J et al. Treatment inequity in antiplatelet therapy for ischaemic heart disease in patients with advanced chronic kidney disease: releasing the evidence vacuum. Platelets 2023;34:2154330. 10.1080/09537104.2022.2154330 36524601

[33] Wu Y, Song Y, Pan Y et al. High on-clopidogrel platelet reactivity and chronic kidney disease: a meta-analysis of literature studies. Scand Cardiovasc J 2019;53:55–61. 10.1080/14017431.2019.1598571 30909763

[34] Orme RC, Parker WAE, Thomas MR et al. Study of two dose regimens of ticagrelor compared with clopidogrel in patients undergoing percutaneous coronary intervention for stable coronary artery disease. Circulation 2018;138:1290–300. 10.1161/CIRCULATIONAHA.118.034790 29930021 PMC6159686

[35] Bazemore TC, Nanna MG, Rao SV. Benefits and risks of P2Y12 inhibitor preloading in patients with acute coronary syndrome and stable angina. J Thromb Thrombolysis 2017;44:303–15. 10.1007/s11239-017-1529-6 28730406

[36] Hughes S, Szeki I, Nash MJ et al. Anticoagulation in chronic kidney disease patients—the practical aspects. Clin Kidney J 2014;7:442–9. 10.1093/ckj/sfu080 25878775 PMC4379338

[37] Blann AD, Landray MJ, Lip GYH. An overview of antithrombotic therapy. BMJ 2002;325:762–5. 10.1136/bmj.325.7367.762 12364307 PMC1124276

[38] Genovesi S, Rossi E, Gallieni M et al. Warfarin use, mortality, bleeding and stroke in haemodialysis patients with atrial fibrillation. Nephrol Dial Transplant 2015;30:491–8. 10.1093/ndt/gfu334 25352571

[39] Wizemann V, Tong L, Satayathum S et al. Atrial fibrillation in hemodialysis patients: clinical features and associations with anticoagulant therapy. Kidney Int 2010;77:1098–106. 10.1038/ki.2009.477 20054291

[40] El-Abbadi M, Giachelli CM. Mechanisms of vascular calcification. Adv Chronic Kidney Dis 2007;14:54–66. 10.1053/j.ackd.2006.10.007 17200044

[41] Caraballo PJ, Heit JA, Atkinson EJ et al. Long-term use of oral anticoagulants and the risk of fracture. Arch Intern Med 1999;159:1750. 10.1001/archinte.159.15.1750 10448778

[42] Ravera M, Bussalino E, Fusaro M et al. Systematic DOACs oral anticoagulation in patients with atrial fibrillation and chronic kidney disease: the nephrologist’s perspective. J Nephrol 2020;33:483–95. 10.1007/s40620-020-00720-5 32200488

[43] Levy JH, Shaw JR, Castellucci LA et al. Reversal of direct oral anticoagulants: guidance from the SSC of the ISTH. J Thromb Haemost 2024;22:2889–99. 10.1016/j.jtha.2024.07.009 39029742

[44] Glund S, Stangier J, Van Ryn J et al. Effect of age and renal function on idarucizumab pharmacokinetics and idarucizumab-mediated reversal of dabigatran anticoagulant activity in a randomized, double-blind, crossover phase Ib study. Clin Pharmacokinet 2017;56:41–54. 10.1007/s40262-016-0417-0 27317414 PMC5222901

[45] Eikelboom JW, Van Ryn J, Reilly P et al. Dabigatran reversal with idarucizumab in patients with renal impairment. J Am Coll Cardiol 2019;74:1760–8. 10.1016/j.jacc.2019.07.070 31582135

[46] Mavromanoli AC, Barco S, Konstantinides SV. Antithrombotics and new interventions for venous thromboembolism: exploring possibilities beyond factor IIa and factor Xa inhibition. Res Prac Thromb Haemost 2021;5:e12509. 10.1002/rth2.12509 PMC8130658 34027284

[47] Steiner D, Kraemmer D, Nopp S et al. A systematic review of safety and efficacy of factor XI/XIa inhibitors in patients with ESKD on hemodialysis. Kidney Int Rep 2025;10:145–56. 10.1016/j.ekir.2024.10.007 39810768 PMC11725973

[48] Developed with the special contribution of the European Heart Rhythm Association (EHRA), Endorsed by the European Association for Cardio-Thoracic Surgery (EACTS), Authors/Task Force Members, Camm A J, Kirchhof P et al. Guidelines for the Management of Atrial Fibrillation: the Task Force for the Management of Atrial Fibrillation of the European Society of Cardiology (ESC). Eur Heart J 2010;31:2369–429. 10.1093/eurheartj/ehq278 20802247

[49] Hau H, Eckert M, Laudi S et al. Predictive Value of HAS-BLED score regarding bleeding events and graft survival following renal transplantation. J Clin Med 2022;11:4025. 10.3390/jcm11144025 35887788 PMC9319563

[50] Ocak G, Ramspek C, Rookmaaker MB et al. Performance of bleeding risk scores in dialysis patients. Nephrol Dial Transplant 2019;34:1223–31. 10.1093/ndt/gfy387 30608543

[51] Van Staa TP, Setakis E, Di Tanna GL et al. A comparison of risk stratification schemes for stroke in 79 884 atrial fibrillation patients in general practice. J Thromb Haemost 2011;9:39–48. 10.1111/j.1538-7836.2010.04085.x 21029359

[52] Sab M, Chelala D, Aoun M et al. Stroke in hemodialysis patients and its association with CHA2DS2-VASC and HAS-BLED scores: a retrospective study. Clin Kidney J 2023;16:596–602. 10.1093/ckj/sfac260 36865009 PMC9972838

[53] Robertson AJ, Nargund V, Gray DWR et al. Low dose aspirin as prophylaxis against renal-vein thrombosis in renal-transplant recipients. Nephrol Dial Transplant 2000;15:1865–8. 10.1093/ndt/15.11.1865 11071979

[54] Rodriguez-Pastore I, Diaz-Corte C, Fernandez-Gonzalez L et al. The platelet transfusion is ineffective to prevent risk of bleeding during renal transplantation surgery in patients on chronic treatment with acetylsalicylic acid. Am J Transplant 2012;12:291–2.

[55] Kapeles S, Cramm S, Srinivasan D et al. Clopidogrel is associated with decreased survival in renal transplant recipients. Am J Transplant 2014;14:77.

[56] Bansal A, Garg P, Kandhari P et al. 242.5: Comparative analysis of perioperative complications in kidney transplant patients with coronary artery disease on dual antiplatelet therapy (DAPT). Transplantation 2022;106:S135. 10.1097/01.tp.0000885992.19440.20

[57] Lee T, D’Souza K, Hameed A et al. Comparison of the effect of single vs dual antiplatelet agents on post-operative haemorrhage after renal transplantation: a systematic review and meta-analysis. Transplant Rev (Orlando) 2021;35:100594. 10.1016/j.trre.2020.100594 33482617

[58] Benkö T, Gottmann M, Radunz S et al. One-year allograft and patient survival in renal transplant recipients receiving antiplatelet therapy at the time of transplantation. Int J Organ Transplant Med 2018;9:10–19. 29531642 PMC5839625

[59] Musetti C, Quaglia M, Cena T et al. Impact of pre-transplant antiaggregant and anticoagulant therapies on early hemorrhagic and cardiovascular events after kidney transplantation. J Nephrol 2015;28:757–64. 10.1007/s40620-015-0185-1 25743391

[60] Bailey PD, Ali H, Lubetzky M et al. Outcomes of kidney transplant recipients on dual antiplatelet therapy. Austin J Nephrol Hypertens 2015;2:1040.

[61] Weng F, Athavale A, Hussain K et al. Complications of dual antiplatelet therapy among recipients of kidney transplants. Am J Transplant 2016;16(Suppl 3):5–798.

[62] Hernández D, Rufino M, Armas S et al. Retrospective analysis of surgical complications following cadaveric kidney transplantation in the modern transplant era. Nephrol Dial Transplant 2006;21:2908–15. 10.1093/ndt/gfl338 16820375

[63] Benahmed A, Kianda M, Ghisdal L et al. Ticlopidine and clopidogrel, sometimes combined with aspirin, only minimally increase the surgical risk in renal transplantation: a case-control study. Nephrol Dial Transplant 2014;29:463–6. 10.1093/ndt/gft385 24275542

[64] Kikić Z, Lorenz M, Sunder-Plassmann G et al. Effect of hemodialysis before transplant surgery on renal allograft function—a pair of randomized controlled trials. Transplantation 2009;88:1377–85. 10.1097/TP.0b013e3181bc03ab 20029334

[65] Connaughton DM, Phelan PJ, Scheult J et al. The impact of peritransplant warfarin use on renal transplant outcome. J Nephrol 2010;23:587–92. 20540039

[66] Wong J, Campbell S, Hawley C et al. Outcomes of kidney transplant recipients on warfarin at time of transplant. Am J Transplant 2006;11:A25.

[67] Littlejohn J, Curtis K, Kutcher M et al. Renal transplantation can be performed safely without pre-operative apixaban reversal. Am J Transplant 2023;23:s579–80.

[68] Lange NW, Lee JH, Liu EC et al. Deceased donor renal transplantation in patients on apixaban at time of transplant surgery: a case series. Clin Transplant 2021;35:e14148. 10.1111/ctr.14148 33222253

[69] Marzouk K, Lawen J, Kiberd BA. Blood transfusion in deceased donor kidney transplantation. Transplant Res 2013;2:4. 10.1186/2047-1440-2-4 23561315 PMC3626660

[70] Eng M, Brock G, Li X et al. Perioperative anticoagulation and antiplatelet therapy in renal transplant: is there an increase in bleeding complication? Clin Transplant 2011;25:292–6. 10.1111/j.1399-0012.2010.01293.x 20529097

[71] Koch M, Kantas A, Ramcke K et al. Surgical complications after kidney transplantation: different impacts of immunosuppression, graft function, patient variables, and surgical performance. Clin Transplant 2015;29:252–60. 10.1111/ctr.12513 25598053

[72] Diamond A, Antonacci C, Lau K et al. Evaluation of clinical outcomes in patients receiving antithrombotic therapy prior to kidney transplantation. Am J Transplant 2020;20:998–9.

[73] Alonso-Escalante JC, Machado L, Tabar KR et al. Is continuing anticoagulation or antiplatelet therapy safe prior to kidney transplantation? Ann Transplant 2021;26:e931648. 10.12659/AOT.931648 PMC8485698 34580271

[74] Buggs J, Shaw R, Montz F et al. Operative versus nonoperative management of hemorrhage in the postoperative kidney transplant patient. Am Surg 2020;86:685–9. 10.1177/0003134820923313 32683955

[75] Halvorsen S, Mehilli J, Cassese S et al. 2022 ESC guidelines on cardiovascular assessment and management of patients undergoing non-cardiac surgery. Eur Heart J 2022;43:3826–924. 10.1093/eurheartj/ehac270 36017553

[76] Douketis JD, Spyropoulos AC, Murad MH et al. Perioperative management of antithrombotic therapy. Chest 2022;162:e207–43. 10.1016/j.chest.2022.07.025 35964704

[77] Ubhi CS, Lam FT, Mavor AID et al. Subcutaneous heparin therapy for cyclosporine-immunosuppressed renal allograft recipients. Transplantation 1989;48:886. 10.1097/00007890-198911000-00038 2815265

[78] Alkhunaizi AM, Olyaei AJ, Barry JM et al. Efficacy and safety of low molecular weight heparin in renal transplantation. Transplantation 1998;66:533–4. 10.1097/00007890-199808270-00020 9734500

[79] Osman Y, Kamal M, Soliman S et al. Necessity of routine postoperative heparinization in non-risky live-donor renal transplantation: results of a prospective randomized trial. Urology 2007;69:647–51. 10.1016/j.urology.2006.12.017 17445644

[80] Lundin C, Bersztel A, Wahlberg J et al. Low molecular weight heparin prophylaxis increases the incidence of lymphocele after kidney transplantation. Ups J Med Sci 2002;107:9–15. 10.3109/2000-1967-137 12296451

[81] Ng JCY, Leung M, Landsberg D. Evaluation of Heparin Anticoagulation Protocols in Post–Renal Transplant Recipients (EHAP-PoRT Study). Can J Hosp Pharm 2016;69:114–21. 10.4212/cjhp.v69i2.1538 PMC4853178 27168632

[82] Qu W, Minkovich M, Clotea I et al. The epidemiology of early deep vein thrombosis in kidney transplant recipients. Can J Surg 2023;66:E162–9. 10.1503/cjs.021821 37001976 PMC10069415

[83] Murphy GJ, Taha R, Windmill DC et al. Influence of aspirin on early allograft thrombosis and chronic allograft nephropathy following renal transplantation. J Br Surg 2001;88:261–6. 10.1046/j.1365-2168.2001.01671.x 11167878

[84] Pegler AH, Hegerty K, Gately RP et al. Incidence of thromboembolic complications following kidney transplantation with short and extended aspirin prophylaxis: a retrospective single-center study. Ann Transplant 2023;28. 10.12659/AOT.939143 PMC10276531 37309097

[85] Faba OR, Boissier R, Budde K et al. European Association of Urology Guidelines on Renal Transplantation: update 2024. Eur Urol Focus 2025;11:365–73. 10.1016/j.euf.2024.10.010. 39489684

[86] Baker RJ, Mark PB, Patel RK et al. Renal association clinical practice guideline in post-operative care in the kidney transplant recipient. BMC Nephrol 2017;18:174. 10.1186/s12882-017-0553-2 28571571 PMC5455080

[87] Eckardt KU, Kasiske BL, Zeier MG. Special Issue: KDIGO Clinical Practice Guideline for the Care of Kidney Transplant Recipients. Am J Transplant 2009;9:S1–155. 10.1111/j.1600-6143.2009.02834.x 19845597

[88] Joglar JA, Chung MK, Armbruster AL et al. 2023 ACC/AHA/ACCP/HRS Guideline for the Diagnosis and Management of Atrial Fibrillation. J Am Coll Cardiol 2024;83:109–279. 10.1016/j.jacc.2023.08.017 38043043 PMC11104284

[89] Bixby AL, Shaikh SA, Naik AS et al. Safety and efficacy of direct-acting oral anticoagulants versus warfarin in kidney transplant recipients: a retrospective single-center cohort study. Transpl Int 2020;33:740–51. 10.1111/tri.13599 32107804

[90] Santoro F, Casanova A, Simone S et al. Immunosuppressive therapy and oral anticoagulation in kidney transplant recipients: direct oral anticoagulants versus vitamin-k antagonists. Eur J Intern Med 2024;119:71–7. 10.1016/j.ejim.2023.08.003 37573220

[91] Basic-Jukic N, Furic-Cunko V, Juric I. Novel oral anticoagulants in renal transplant recipients: a retrospective cohort study. Pril (Makedon Akad Nauk Umet Odd Med Nauki) 2020;41:49–55. 10.2478/prilozi-2020-0032 33011699

[92] Bukhari MA, Al-Theaby A, Tawhari M et al. Efficacy and safety of non-vitamin K antagonist oral anticoagulants post-kidney transplantation. World J Transplant 2019;9:134–44. 10.5500/wjt.v9.i6.134 31750090 PMC6851500

[93] Green H, Rahamimov R, Spectre G et al. Apixaban level and its influence on immunosuppression and graft outcome in kidney transplant recipients with atrial fibrillation. Ther Drug Monit 2021;43:637–44. 10.1097/FTD.0000000000000858 33337589

[94] Parker K, Chu J, Morton M et al. Can direct oral anticoagulants be used in kidney transplant recipients? Clin Transplant 2021;35:e14474. 10.1111/ctr.14474 34498777

[95] Firth C, Shamoun F, Apolinario M et al. Safety and mortality outcomes for direct oral anticoagulants in renal transplant recipients. PLoS One 2023;18:e0285412. 10.1371/journal.pone.0285412 37192210 PMC10187891

[96] Zaitoun MF, Sheikh ME, Faifi ASA et al. The use of non–vitamin K antagonist oral anticoagulants in post-kidney transplantation, single-center experience. Transplant Proc 2021;53:2918–22. 10.1016/j.transproceed.2021.09.042 34772494

[97] Pfrepper C, Herber A, Weimann A et al. Safety and efficacy of direct oral anticoagulants under long-term immunosuppressive therapy after liver, kidney and pancreas transplantation. Transpl Int 2021;34:423–35. 10.1111/tri.13804 33336411

[98] Leon J, Sabbah L, Aubert O et al. Efficacy and safety of direct oral anticoagulants in kidney transplantation: a single-center pilot experience. Transplantation 2020;104:2625–31. 10.1097/TP.0000000000003168 32044892

[99] Camporese G, Bernardi D, Bernardi E et al. Absence of interaction between rivaroxaban, tacrolimus and everolimus in renal transplant recipients with deep vein thrombosis or atrial fibrillation. Vasc Pharmacol 2020;130:106682. 10.1016/j.vph.2020.106682 32438078

[100] Do V, Haakinson D, Belfield K. When to hold: apixaban in renal transplant recipients. Am J Transplant 2019;19:591. 10.1111/ajt.1540630346652

[101] Mansell H, Shoker A, Alcorn J et al. Pharmacokinetics of apixaban and tacrolimus or cyclosporine in kidney and lung transplant recipients. Clin Transl Sci 2022;15:1687–97. 10.1111/cts.13284 35439353 PMC9283751

[102] Vanhove T, Spriet I, Annaert P et al. Effect of the direct oral anticoagulants rivaroxaban and apixaban on the disposition of calcineurin inhibitors in transplant recipients. Ther Drug Monit 2017;39:77–82. 10.1097/FTD.0000000000000356 27861314

[103] Morgan TA, Chandran S, Burger IM et al. Complications of ultrasound-guided renal transplant biopsies. Am J Transplant 2016;16:1298–305. 10.1111/ajt.13622 26601796

[104] Baffour FI, Hickson LJ, Stegall MD et al. Effects of aspirin therapy on ultrasound–guided renal allograft biopsy bleeding complications. J Vasc Interv Radiol 2017;28:188–94. 10.1016/j.jvir.2016.10.021 27993506 PMC5258683

[105] Li CH, Traube LE, Lu DS et al. Implementation and results of a percutaneous renal allograft biopsy protocol to reduce complication rate. J Am Coll Radiol 2016;13:549–53. 10.1016/j.jacr.2015.12.003 26970700

[106] Kuiper GJAJM, Christiaans MHL, Mullens MHJM et al. Routine haemostasis testing before transplanted kidney biopsy: a cohort study. Transpl Int 2018;31:302–12. 10.1111/tri.13090 29108097

[107] Mattiazzi AD, Cortesi CA, Patil RJ et al. Percutaneous ultrasound-guided kidney transplant biopsy outcomes: from the nephrologist to the radiologist standpoint. Kidney360 2022;3:1746–53. 10.34067/KID.0000332022 36514719 PMC9717654

[108] Maxey-Jones C, Seelhammer TG, Arabia FA et al. TEG® 6s–guided algorithm for optimizing patient blood management in cardiovascular surgery: systematic literature review and expert opinion. J Cardiothorac Vasc Anesth 2025;39:1162–72. 10.1053/j.jvca.2025.02.011 40016048

[109] Chan N, Sobieraj-Teague M, Eikelboom JW. Direct oral anticoagulants: evidence and unresolved issues. Lancet 2020;396:1767–76. 10.1016/S0140-6736(20)32439-9 33248499

[110] Ades M, Simard C, Vanassche T et al. Factor XI inhibitors: potential role in end-stage kidney disease. Semin Nephrol 2023;43:151484. 10.1016/j.semnephrol.2023.151484 38272779

[111] Weitz JI, Tankó LB, Floege J et al. Anticoagulation with osocimab in patients with kidney failure undergoing hemodialysis: a randomized phase 2 trial. Nat Med 2024;30:435–42. 10.1038/s41591-023-02794-7 38365952 PMC10878964

