# Supplementary material for: Management of antithrombotic therapies in kidney transplantation candidates and recipients: an ERBP clinical practice document by the DESCaRTES Working Group of ERA and the EKITA Committee of ESOT
Source: Nephrol Dial Transplant. 2026 Feb 9;41(9):1736–54. doi: 10.1093/ndt/gfag024 (PMC13518092; doi:10.1093/ndt/gfag024)
Supplement: Supplementary file 1 [file gfag024_supplemental_file.docx]

**MANAGEMENT OF ANTITHROMBOTIC THERAPIES IN KIDNEY TRANSPLANTATION CANDIDATES AND RECIPIENTS: AN ERBP (EUROPEAN RENAL BEST PRACTICE) CLINICAL PRACTICE DOCUMENT BY THE DESCARTES WORKING GROUP OF THE EUROPEAN RENAL ASSOCIATION (ERA) AND THE EKITA COMMITTEE OF THE EUROPEAN SOCIETY OF ORGAN TRANSPLANTATION (ESOT).**

# **1. DEVELOPING CLINICAL QUESTIONS AND FRAMING RELEVANT PICO**

Four specific clinical questions were developed, and a systematic review was conducted for each one of them. These questions are listed in **Supplementary Table 1**.

**Supplementary Table 1:** Clinical questions for the development of guidelines.

| 1 | What is the peri-operative bleeding risk in kidney transplant candidates who are on antiplatelet or anticoagulant therapies? |
| --- | --- |
| 2 | Should kidney transplant recipients receive prophylaxis for venous thrombosis after surgery? |
| 3 | Can we use direct-acting oral anticoagulants (DOACs) in kidney transplant recipients in terms of safety (dosing, drug- drug interactions, adverse events)? |
| 4 | Is there a need for interruption of antiplatelet therapy before graft biopsy? |

A methodologist assisted in framing the clinical questions into searchable formats, by applying the PICO procedure (or its variations, e.g., PECO). This required careful specification of the patient group (P), intervention (I) or exposure (E) where applicable, comparator (C) and outcomes (O). Below PICO frameworks are displayed in detail for all four clinical questions.

**Supplementary Table 2:** Question 1 PICO - What is the peri-operative bleeding risk in kidney transplant candidates who are on antiplatelet or anticoagulant therapies?

| **P**opulation | End stage kidney disease or eGFR <15 mL/min/1.73 m^2^ |
| --- | --- |
|  | Kidney transplant candidates |
|  | Kidney transplant recipients |
| **E**xposure / **I**ntervention | Antiplatelet therapy |
|  | Anticoagulant therapy |
| **C**omparator | Any antithrombotic treatment |
|  | No antithrombotic treatment |
| **O**utcome | Bleeding events |
|  | Thrombotic events |
|  | Other related events (cardiovascular events, graft survival, patient survival, delayed graft function, etc.) |
| **T**iming | Peri-operative |

**Supplementary Table 3:** Question 2 PICO - Should kidney transplant recipients receive prophylaxis for venous thrombosis after surgery?

| **P**opulation | Kidney transplant recipients |
| --- | --- |
| **E**xposure / **I**ntervention | Anticoagulant therapy |
| **C**omparator | Any anticoagulant treatment |
|  | No anticoagulant treatment |
| **O**utcome | Bleeding events |
|  | Thrombotic events |
|  | Other related events (cardiovascular events, graft survival, patient survival, delayed graft function, etc.) |
| **T**iming | Post-operative |

**Supplementary Table 4:** Question 3 PICO - Can we use direct-acting oral anticoagulants (DOACs) in kidney transplant recipients in terms of safety (dosing, drug- drug interactions, adverse events)?

| **P**opulation | Kidney transplant recipients |
| --- | --- |
| **E**xposure / **I**ntervention | Direct-acting oral anticoagulants (DOACs) |
| **C**omparator | Any antithrombotic treatment |
|  | No antithrombotic treatment |
| **O**utcome | Bleeding events |
|  | Thrombotic events |
|  | Other related events (cardiovascular events, graft survival, patient survival, etc.) |
|  | Drug-drug interaction |

**Supplementary Table 5:** Question 4 PICO - Is there a need for interruption of antiplatelet therapy before graft biopsy?

| **P**opulation | Kidney transplant recipients |
| --- | --- |
| **E**xposure / **I**ntervention | Antiplatelet treatment |
| **C**omparator | No antiplatelet treatment |
| **O**utcome | Bleeding events |
|  | Thrombotic events |
|  | Other related events (cardiovascular events, graft survival, patient survival, etc.) |
| **T**iming | Prior to kidney graft biopsy |

# **For all four questions, after experts’ consensus, paediatric and high-risk patients were excluded.**

# **2. SEARCHING FOR EVIDENCE – SEARCH STRATEGY**

The methodologist searched 3 major databases (Pubmed, Cochrane Library, Web of Science), from inception to March 31, 2024, for all 4 questions. The search strategies used all relevant search terms, i.e., free-text terms (including synonyms, alternative names, acronyms, plurals) and controlled vocabulary terms (e.g., MeSH terms for PubMED database) that were related to the patient population and intervention/exposure; then the search terms were combined using appropriate Boolean operators. No search filter for language or study type was applied. The detailed search strategies for each research question in all 3 databases are provided below.

**Supplementary Table 6:** Search strategy for Question 1 - What is the peri-operative bleeding risk in kidney transplant candidates who are on antiplatelet or anticoagulant therapies?

**SEARCH STRATEGY FOR MEDLINE via PubMed**

A. **SEARCH SYNTAX**

1. kidney transplantation [MeSH Terms]
2. kidney transplantation [All Fields]
3. renal transplantation [All Fields]
4. kidney transplant recipient* [All Fields]
5. renal transplant recipient* [All Fields]
6. kidney transplant patient* [All Fields]
7. renal transplant patient* [All Fields]
8. kidney transplant candidate* [All Fields]
9. renal transplant candidate* [All Fields]
10. kidney graft [All Fields]
11. renal graft [All Fields]
12. kidney allograft [All Fields]
13. renal allograft [All Fields]
14. kidney grafting [All Fields]
15. renal grafting [All Fields]
16. #1 OR #2 OR #3 OR #4 OR #5 OR #6 OR #7 OR #8 OR #9 OR #10 OR #11 OR #12 OR #13 OR #14 OR #15
17. antithrombotic [All Fields]
18. antiplatelet [All Fields]
19. platelet aggregation inhibitors [MeSH Terms]
20. anticoagulant [All Fields]
21. anticoagulants [MeSH Terms]
22. anticoagulation [All Fields]
23. thromboprophylactic [All Fields]
24. thromboprophylaxis [All Fields]
25. thrombosis prophylaxis [All Fields]
26. thrombosis prevention [All Fields]
27. thromboembolism prophylaxis [All Fields]
28. thromboembolism prevention [All Fields]
29. VTE prophylaxis [All Fields]
30. VTE prevention [All Fields]
31. DVT prophylaxis [All Fields]
32. DVT prevention [All Fields]
33. heparin [All Fields]
34. unfractionated heparin [All Fields]
35. heparin [MeSH Terms]
36. low molecular weight heparin [All Fields]
37. Heparin, Low-Molecular-Weight [MeSH Terms]
38. LMWH [All Fields]
39. bemiparin [All Fields]
40. bemiparin [Supplementary concept]
41. dalteparin [All Fields]
42. dalteparin [MeSH Terms]
43. enoxaparin [All Fields]
44. enoxaparin [MeSH Terms]
45. tinzaparin [All Fields]
46. tinzaparin [MeSH Terms]
47. nadroparin [All Fields]
48. nadroparin [MeSH Terms]
49. fondaparinux [All Fields]
50. fondaparinux [MeSH Terms]
51. parnaparin [All Fields]
52. parnaparin [Supplementary concept]
53. reviparin [All Fields]
54. reviparin [Supplementary concept]
55. certoparin [All Fields]
56. certoparin [Supplementary concept]
57. Antithrombin [All Fields]
58. Antithrombins [MeSH Terms]
59. direct thrombin inhibitor* [All Fields]
60. Xa inhibitor* [All Fields]
61. Factor Xa Inhibitors [MeSH Terms]
62. IIa inhibitor*
63. dabigatran [All Fields]
64. dabigatran [MeSH Terms]
65. rivaroxaban [All Fields]
66. rivaroxaban [MeSH Terms]
67. apixaban [All Fields]
68. apixaban [Supplementary Concept]
69. edoxaban [All Fields]
70. edoxaban [Supplementary Concept]
71. novel oral anticoagulant* [All Fields]
72. direct oral anticoagulant* [All Fields]
73. NOAC* [All Fields]
74. DOAC* [All Fields]
75. hirudin [All Fields]
76. hirudins [MeSH Terms]
77. desirudin [All Fields]
78. desirudin [Supplementary Concept]
79. lepirudin [All Fields]
80. lepirudin [Supplementary Concept]
81. bivalirudin [All Fields]
82. bivalirudin [Supplementary Concept]
83. argatroban [All Fields]
84. argatroban [Supplementary Concept]
85. Vitamin K Antagonist* [All Fields]
86. VKA [All Fields]
87. warfarin [All Fields]
88. warfarin [MeSH Terms]
89. acenocoumarol [All Fields]
90. acenocoumarol [MeSH Terms]
91. phenprocoumon [All Fields]
92. phenprocoumon [MeSH Terms]
93. aspirin [All Fields]
94. acetylsalicylic [All Fields]
95. aspirin [MeSH Terms]
96. dipyridamole [All Fields]
97. dipyridamole [MeSH Terms]
98. clopidogrel [All Fields]
99. clopidogrel [MeSH Terms]
100. prasugrel [All Fields]
101. prasugrel hydrochloride [MeSH Terms]
102. ticagrelor [All Fields]
103. ticagrelor [MeSH Terms]
104. cangrelor [All Fields]
105. cangrelor [Supplementary Concept]
106. ticlopidine [All Fields]
107. ticlopidine [MeSH Terms]
108. vorapaxar [All Fields]
109. vorapaxar [Supplementary Concept]
110. cilostazol [All Fields]
111. cilostazol [MeSH Terms]
112. glycoprotein IIb/IIIa receptor blocker* [All Fields]
113. abciximab [All Fields]
114. abciximab [MeSH Terms]
115. eptifibatide [All Fields]
116. eptifibatide [MeSH Terms]
117. tirofiban [All Fields]
118. tirofiban [MeSH Terms]
119. bridging anticoagulation [All Fields]
120. bridging anticoagulant [All Fields]
121. #17 OR #18 OR #19 OR #20 OR #21 OR #22 OR #23 OR #24 OR #25 OR #26 OR #27 OR #28 OR #29 OR #30 OR #31 OR #32 OR #33 OR #34 OR #35 OR #36 OR #37 OR #38 OR #39 OR #40 OR #41 OR #42 OR #43 OR #44 OR #45 OR #46 OR #47 OR #48 OR #49 OR #50 OR #51 OR #52 OR #53 OR #54 OR #55 OR #56 OR #57 OR #58 OR #59 OR #60 OR #61 OR #62 OR #63 OR #64 OR #65 OR #66 OR #67 OR #68 OR #69 OR #70 OR #71 OR #72 OR #73 OR #74 OR #75 OR #76 OR #77 OR #78 OR #79 OR #80 OR #81 OR #82 OR #83 OR #84 OR #85 OR #86 OR #87 OR #88 OR #89 OR #90 OR #91 OR #92 OR #93 OR #94 OR #95 OR #96 OR #97 OR #98 OR #99 OR #100 OR #101 OR #102 OR #103 OR #104 OR #105 OR #106 OR #107 OR #108 OR #109 OR #110 OR #111 OR #112 OR #113 OR #114 OR #115 OR #116 OR #117 OR #118 OR #119 OR #120
122. #16 AND #121

B. **SEARCH STRING**

**(**"kidney transplantation"[MeSH Terms] OR "kidney transplantation"[All Fields] OR "renal transplantation"[All Fields] OR "kidney transplant recipient*"[All Fields] OR "renal transplant recipient*"[All Fields] OR "kidney transplant patient*"[All Fields] OR "renal transplant patient*"[All Fields] OR "kidney transplant candidate*"[All Fields] OR "renal transplant candidate*"[All Fields] OR "kidney graft"[All Fields] OR "renal graft"[All Fields] OR "kidney allograft"[All Fields] OR "renal allograft"[All Fields] OR "kidney grafting"[All Fields] OR "renal grafting"[All Fields]**)** **AND (**"antithrombotic"[All Fields] OR "antiplatelet"[All Fields] OR "platelet aggregation inhibitors"[MeSH Terms] OR "anticoagulant"[All Fields] OR "anticoagulants"[MeSH Terms] OR "anticoagulation"[All Fields] OR "thromboprophylactic"[All Fields] OR "thromboprophylaxis"[All Fields] OR "thrombosis prophylaxis"[All Fields] OR "thrombosis prevention"[All Fields] OR "thromboembolism prophylaxis"[All Fields] OR "thromboembolism prevention"[All Fields] OR "VTE prophylaxis"[All Fields] OR "VTE prevention"[All Fields] OR "DVT prophylaxis"[All Fields] OR "DVT prevention"[All Fields] OR "heparin"[All Fields] OR "unfractionated heparin"[All Fields] OR "heparin"[MeSH Terms] OR "low molecular weight heparin"[All Fields] OR "Heparin, Low-Molecular-Weight"[MeSH Terms] OR "LMWH"[All Fields] OR "bemiparin"[All Fields] OR "bemiparin"[Supplementary concept] OR "dalteparin"[All Fields] OR "dalteparin"[MeSH Terms] OR "enoxaparin"[All Fields] OR "enoxaparin"[MeSH Terms] OR "tinzaparin"[All Fields] OR "tinzaparin"[MeSH Terms] OR "nadroparin"[All Fields] OR "nadroparin"[MeSH Terms] OR "fondaparinux"[All Fields] OR "fondaparinux"[MeSH Terms] OR "parnaparin"[All Fields] OR "parnaparin"[Supplementary concept] OR "reviparin"[All Fields] OR "reviparin"[Supplementary concept] OR "certoparin"[All Fields] OR "certoparin"[Supplementary concept] OR "Antithrombin"[All Fields] OR "Antithrombins"[MeSH Terms] OR "direct thrombin inhibitor*"[All Fields] OR "Xa inhibitor*"[All Fields] OR "Factor Xa Inhibitors"[MeSH Terms] OR "IIa inhibitor*"[All Fields] OR "dabigatran"[All Fields] OR "dabigatran"[MeSH Terms] OR "rivaroxaban"[All Fields] OR "rivaroxaban"[MeSH Terms] OR "apixaban"[All Fields] OR "apixaban"[Supplementary Concept] OR "edoxaban"[All Fields] OR "edoxaban"[ Supplementary Concept] OR "novel oral anticoagulant*"[All Fields] OR "direct oral anticoagulant*"[All Fields] OR "NOAC*"[All Fields] OR "DOAC*"[All Fields] OR "hirudin"[All Fields] OR "hirudins"[MeSH Terms] OR "desirudin"[All Fields] OR "desirudin"[Supplementary Concept] OR "lepirudin"[All Fields] OR "lepirudin"[Supplementary Concept] OR "bivalirudin"[All Fields] OR "bivalirudin"[Supplementary Concept] OR "argatroban"[All Fields] OR "argatroban"[Supplementary Concept] OR "Vitamin K Antagonist*"[All Fields] OR "VKA"[All Fields] OR "warfarin"[All Fields] OR "warfarin"[MeSH Terms] OR "acenocoumarol"[All Fields] OR "acenocoumarol"[MeSH Terms] OR "phenprocoumon"[All Fields] OR "phenprocoumon"[MeSH Terms] OR "aspirin"[All Fields] OR "acetylsalicylic"[All Fields] OR "aspirin"[MeSH Terms] OR " dipyridamole"[All Fields] OR " dipyridamole"[MeSH Terms] OR "clopidogrel"[All Fields] OR "clopidogrel"[MeSH Terms] OR "prasugrel"[All Fields] OR " prasugrel hydrochloride "[MeSH Terms] OR "ticagrelor"[All Fields] OR "ticagrelor"[MeSH Terms] OR "cangrelor"[All Fields] OR "cangrelor"[Supplementary Concept] OR "ticlopidine"[All Fields] OR "ticlopidine"[MeSH Terms] OR "vorapaxar"[All Fields] OR "vorapaxar"[Supplementary Concept] OR "cilostazol"[All Fields] OR "cilostazol"[MeSH Terms] OR "glycoprotein IIb/IIIa receptor blocker*"[All Fields] OR "abciximab"[All Fields] OR "abciximab"[MeSH Terms] OR "eptifibatide"[All Fields] OR "eptifibatide"[MeSH Terms] OR "tirofiban"[All Fields] OR "tirofiban"[MeSH Terms] OR "bridging anticoagulation"[All Fields] OR "bridging anticoagulant"[All Fields]**)**

**SEARCH STRATEGY FOR COCHRANE LIBRARY**

A. **SEARCH SYNTAX**

1. MeSH descriptor: [Kidney Transplantation] explode all trees
2. kidney transplantation
3. renal transplantation
4. kidney transplant recipient*
5. renal transplant recipient*
6. kidney transplant patient*
7. renal transplant patient*
8. kidney transplant candidate*
9. renal transplant candidate*
10. kidney graft
11. renal graft
12. kidney allograft
13. renal allograft
14. kidney grafting
15. renal grafting
16. #1 OR #2 OR #3 OR #4 OR #5 OR #6 OR #7 OR #8 OR #9 OR #10 OR #11 OR #12 OR #13 OR #14 OR #15
17. antithrombotic*
18. antiplatelet*
19. MeSH descriptor: [platelet aggregation inhibitors] explode all trees
20. anticoagulant*
21. MeSH descriptor: [Anticoagulants] explode all trees
22. anticoagulation
23. thromboprophylactic
24. thromboprophylaxis
25. thrombosis prophylaxis
26. thrombosis prevention
27. thromboembolism prophylaxis
28. thromboembolism prevention
29. VTE prophylaxis
30. VTE prevention
31. DVT prophylaxis
32. DVT prevention
33. heparin
34. unfractionated heparin
35. MeSH descriptor: [Heparin] explode all trees
36. low molecular weight heparin
37. LMWH
38. MeSH descriptor: [Heparin, Low-Molecular-Weight] explode all trees
39. bemiparin
40. dalteparin
41. MeSH descriptor: [Dalteparin] explode all trees
42. enoxaparin
43. MeSH descriptor: [Enoxaparin] explode all trees
44. tinzaparin
45. MeSH descriptor: [Tinzaparin] explode all trees
46. nadroparin
47. MeSH descriptor: [Nadroparin] explode all trees
48. fondaparinux
49. MeSH descriptor: [Fondaparinux] explode all trees
50. parnaparin
51. reviparin
52. certoparin
53. Antithrombin
54. MeSH descriptor: [Antithrombins] explode all trees
55. direct thrombin inhibitor*
56. Xa inhibitor*
57. MeSH descriptor: [Factor Xa Inhibitors] explode all trees
58. IIa inhibitor*
59. Dabigatran
60. MeSH descriptor: [Dabigatran] explode all trees
61. Rivaroxaban
62. MeSH descriptor: [Rivaroxaban] explode all trees
63. apixaban
64. edoxaban
65. novel oral anticoagulant*
66. direct oral anticoagulant*
67. NOAC*
68. DOAC*
69. hirudin
70. MeSH descriptor: [Hirudins] explode all trees
71. desirudin
72. lepirudin
73. bivalirudin
74. argatroban
75. Vitamin K Antagonist*
76. VKA*
77. warfarin
78. MeSH descriptor: [Warfarin] explode all trees
79. acenocoumarol
80. MeSH descriptor: [Acenocoumarol] explode all trees
81. Phenprocoumon
82. MeSH descriptor: [Phenprocoumon] explode all trees
83. aspirin
84. acetylsalicylic
85. MeSH descriptor: [Aspirin] explode all trees
86. dipyridamole
87. MeSH descriptor: [Dipyridamole] explode all trees
88. clopidogrel
89. MeSH descriptor: [Clopidogrel] explode all trees
90. prasugrel
91. MeSH descriptor: [Prasugrel Hydrochloride] explode all trees
92. ticagrelor
93. MeSH descriptor: [Ticagrelor] explode all trees
94. cangrelor
95. ticlopidine
96. MeSH descriptor: [Ticlopidine] explode all trees
97. vorapaxar
98. cilostazol
99. MeSH descriptor: [Cilostazol] explode all trees
100. glycoprotein IIb IIIa receptor blocker*
101. abciximab
102. MeSH descriptor: [Abciximab] explode all trees
103. eptifibatide
104. MeSH descriptor: [Eptifibatide] explode all trees
105. tirofiban
106. MeSH descriptor: [Tirofiban] explode all trees
107. bridging anticoagulation
108. bridging anticoagulant*
109. #17 OR #18 OR #19 OR #20 OR #21 OR #22 OR #23 OR #24 OR #25 OR #26 OR #27 OR #28 OR #29 OR #30 OR #31 OR #32 OR #33 OR #34 OR #35 OR #36 OR #37 OR #38 OR #39 OR #40 OR #41 OR #42 OR #43 OR #44 OR #45 OR #46 OR #47 OR #48 OR #49 OR #50 OR #51 OR #52 OR #53 OR #54 OR #55 OR #56 OR #57 OR #58 OR #59 OR #60 OR #61 OR #62 OR #63 OR #64 OR #65 OR #66 OR #67 OR #68 OR #69 OR #70 OR #71 OR #72 OR #73 OR #74 OR #75 OR #76 OR #77 OR #78 OR #79 OR #80 OR #81 OR #82 OR #83 OR #84 OR #85 OR #86 OR #87 OR #88 OR #89 OR #90 OR #91 OR #92 OR #93 OR #94 OR #95 OR #96 OR #97 OR #98 OR #99 OR #100 OR #101 OR #102 OR #103 OR #104 OR #105 OR #106 OR #107 OR #108
110. #16 AND #109

B. **SEARCH STRING**

**(**(MeSH descriptor: [Kidney Transplantation] explode all trees) OR (kidney transplantation) OR (renal transplantation) OR (kidney transplant recipient*) OR (renal transplant recipient*) OR (kidney transplant patient*) OR (renal transplant patient*) OR (kidney transplant candidate*) OR (renal transplant candidate*) OR (kidney graft) OR (renal graft) OR (kidney allograft) OR (renal allograft) OR (kidney grafting) OR (renal grafting)**) AND (**(antithrombotic*) OR (antiplatelet*) OR (MeSH descriptor: [platelet aggregation inhibitors] explode all trees) OR (anticoagulant*) OR (MeSH descriptor: [Anticoagulants] explode all trees) OR (anticoagulation) OR (thromboprophylactic) OR (thromboprophylaxis) OR (thrombosis prophylaxis) OR (thrombosis prevention) OR (thromboembolism prophylaxis) OR (thromboembolism prevention) OR (VTE prophylaxis) OR (VTE prevention) OR (DVT prophylaxis) OR (DVT prevention) OR (heparin) OR (unfractionated heparin) OR (MeSH descriptor: [Heparin] explode all trees) OR (low molecular weight heparin) OR (LMWH) OR (MeSH descriptor: [Heparin, Low-Molecular-Weight] explode all trees) OR (bemiparin) OR (dalteparin) OR (MeSH descriptor: [Dalteparin] explode all trees) OR (enoxaparin) OR (MeSH descriptor: [Enoxaparin] explode all trees) OR (tinzaparin) OR (MeSH descriptor: [Tinzaparin] explode all trees) OR (nadroparin) OR (MeSH descriptor: [Nadroparin] explode all trees) OR (fondaparinux) OR (MeSH descriptor: [Fondaparinux] explode all trees) OR (parnaparin) OR (reviparin) OR (certoparin) OR (Antithrombin) OR (MeSH descriptor: [Antithrombins] explode all trees) OR (direct thrombin inhibitor*) OR (Xa inhibitor*) OR (MeSH descriptor: [Factor Xa Inhibitors] explode all trees) OR (IIa inhibitor*) OR (Dabigatran) OR (MeSH descriptor: [Dabigatran] explode all trees) OR (Rivaroxaban) OR (MeSH descriptor: [Rivaroxaban] explode all trees) OR (apixaban) OR (edoxaban) OR (novel oral anticoagulant*) OR (direct oral anticoagulant*) OR (NOAC*) OR (DOAC*) OR (hirudin) OR (MeSH descriptor: [Hirudins] explode all trees) OR (desirudin) OR (lepirudin) OR (bivalirudin) OR (argatroban) OR (Vitamin K Antagonist*) OR (VKA*) OR (warfarin) OR (MeSH descriptor: [Warfarin] explode all trees) OR (acenocoumarol) OR (MeSH descriptor: [Acenocoumarol] explode all trees) OR (Phenprocoumon) OR (MeSH descriptor: [Phenprocoumon] explode all trees) OR (aspirin) OR (acetylsalicylic) OR (MeSH descriptor: [Aspirin] explode all trees) OR (dipyridamole) OR (MeSH descriptor: [Dipyridamole] explode all trees) OR (clopidogrel) OR (MeSH descriptor: [Clopidogrel] explode all trees) OR (prasugrel) OR (MeSH descriptor: [Prasugrel Hydrochloride] explode all trees) OR (ticagrelor) OR (MeSH descriptor: [Ticagrelor] explode all trees) OR (cangrelor) OR (ticlopidine) OR (MeSH descriptor: [Ticlopidine] explode all trees) OR (vorapaxar) OR (cilostazol) OR (MeSH descriptor: [Cilostazol] explode all trees) OR (glycoprotein IIb IIIa receptor blocker*) OR (abciximab) OR (MeSH descriptor: [Abciximab] explode all trees) OR (eptifibatide) OR (MeSH descriptor: [Eptifibatide] explode all trees) OR (tirofiban) OR (MeSH descriptor: [Tirofiban] explode all trees) OR (bridging anticoagulation) OR (bridging anticoagulant*)**)**

**SEARCH STRATEGY FOR WEB OF SCIENCE**

A. **SEARCH SYNTAX**

1. ALL=("kidney transplantation")
2. ALL=("renal transplantation")
3. ALL=("kidney transplant recipient*")
4. ALL=("renal transplant recipient*")
5. ALL=("kidney transplant patient*")
6. ALL=("renal transplant patient*")
7. ALL=("kidney transplant candidate*")
8. ALL=("renal transplant candidate*")
9. ALL=("kidney graft")
10. ALL=("renal graft")
11. ALL=("kidney allograft")
12. ALL=("renal allograft")
13. ALL=("kidney grafting")
14. ALL=("renal grafting"
15. #1 OR #2 OR #3 OR #4 OR #5 OR #6 OR #7 OR #8 OR #9 OR #10 OR #11 OR #12 OR #13 OR #14 OR #15
16. ALL=("antithrombotic")
17. ALL=("antiplatelet")
18. ALL=("platelet aggregation inhibitors")
19. ALL=("anticoagulant*")
20. ALL=("anticoagulation")
21. ALL=("thromboprophylactic")
22. ALL=("thromboprophylaxis")
23. ALL=("thrombosis prophylaxis")
24. ALL=("thrombosis prevention")
25. ALL=("thromboembolism prophylaxis")
26. ALL=("thromboembolism prevention")
27. ALL=("VTE prophylaxis")
28. ALL=("VTE prevention")
29. ALL=("DVT prophylaxis")
30. ALL=("DVT prevention")
31. ALL=("heparin")
32. ALL=("unfractionated heparin")
33. ALL=("low molecular weight heparin")
34. ALL=("LMWH")
35. ALL=("bemiparin")
36. ALL=("dalteparin")
37. ALL=("enoxaparin")
38. ALL=("tinzaparin")
39. ALL=("nadroparin")
40. ALL=("fondaparinux")
41. ALL=("parnaparin")
42. ALL=("reviparin")
43. ALL=("certoparin")
44. ALL=("Antithrombin")
45. ALL=("direct thrombin inhibitor*")
46. ALL=("Xa inhibitor*")
47. ALL=("Factor Xa Inhibitors")
48. ALL=("IIa inhibitor*")
49. ALL=("dabigatran")
50. ALL=("rivaroxaban")
51. ALL=("apixaban")
52. ALL=("edoxaban")
53. ALL=("novel oral anticoagulant*")
54. ALL=("direct oral anticoagulant*")
55. ALL=("NOAC*")
56. ALL=("DOAC*")
57. ALL=("hirudin")
58. ALL=("desirudin")
59. ALL=("lepirudin")
60. ALL=("bivalirudin")
61. ALL=("argatroban")
62. ALL=("Vitamin K Antagonist*")
63. ALL=("VKA")
64. ALL=("warfarin")
65. ALL=("acenocoumarol")
66. ALL=("phenprocoumon")
67. ALL=("aspirin")
68. ALL=("acetylsalicylic")
69. ALL=("dipyridamole")
70. ALL=("clopidogrel")
71. ALL=("prasugrel")
72. ALL=("ticagrelor")
73. ALL=("cangrelor")
74. ALL=("ticlopidine")
75. ALL=("vorapaxar")
76. ALL=("cilostazol")
77. ALL=("glycoprotein IIb/IIIa receptor blocker*")
78. ALL=("abciximab")
79. ALL=("eptifibatide")
80. ALL=("tirofiban")
81. ALL=("bridging anticoagulation")
82. ALL=("bridging anticoagulant")
83. #16 OR #17 OR #18 OR #19 OR #20 OR #21 OR #22 OR #23 OR #24 OR #25 OR #26 OR #27 OR #28 OR #29 OR #30 OR #31 OR #32 OR #33 OR #34 OR #35 OR #36 OR #37 OR #38 OR #39 OR #40 OR #41 OR #42 OR #43 OR #44 OR #45 OR #46 OR #47 OR #48 OR #49 OR #50 OR #51 OR #52 OR #53 OR #54 OR #55 OR #56 OR #57 OR #58 OR #59 OR #60 OR #61 OR #62 OR #63 OR #64 OR #65 OR #66 OR #67 OR #68 OR #69 OR #70 OR #71 OR #72 OR #73 OR #74 OR #75 OR #76 OR #77 OR #78 OR #79 OR #80 OR #81 OR #82
84. #15 AND #83

B. **SEARCH STRING**

**(**ALL=("kidney transplantation") OR ALL=("renal transplantation") OR ALL=("kidney transplant recipient*") OR ALL=("renal transplant recipient*") OR ALL=("kidney transplant patient*") OR ALL=("renal transplant patient*") OR ALL=("kidney transplant candidate*") OR ALL=("renal transplant candidate*") OR ALL=("kidney graft") OR ALL=("renal graft") OR ALL=("kidney allograft") OR ALL=("renal allograft") OR ALL=("kidney grafting") OR ALL=("renal grafting")**)** **AND** **(**ALL=("antithrombotic") OR ALL=("antiplatelet") OR ALL=("platelet aggregation inhibitors") OR ALL=("anticoagulant*") OR ALL=("anticoagulation") OR ALL=("thromboprophylactic") OR ALL=("thromboprophylaxis") OR ALL=("thrombosis prophylaxis") OR ALL=("thrombosis prevention") OR ALL=("thromboembolism prophylaxis") OR ALL=("thromboembolism prevention") OR ALL=("VTE prophylaxis") OR ALL=("VTE prevention") OR ALL=("DVT prophylaxis") OR ALL=("DVT prevention") OR ALL=("heparin") OR ALL=("unfractionated heparin") OR ALL=("low molecular weight heparin") OR ALL=("LMWH") OR ALL=("bemiparin") OR ALL=("dalteparin") OR ALL=("enoxaparin") OR ALL=("tinzaparin") OR ALL=("nadroparin") OR ALL=("fondaparinux") OR ALL=("parnaparin") OR ALL=("reviparin") OR ALL=("certoparin") OR ALL=("Antithrombin") OR ALL=("direct thrombin inhibitor*") OR ALL=("Xa inhibitor*") OR ALL=("Factor Xa Inhibitors") OR ALL=("IIa inhibitor*") OR ALL=("dabigatran") OR ALL=("rivaroxaban") OR ALL=("apixaban") OR ALL=("edoxaban") OR ALL=("novel oral anticoagulant*") OR ALL=("direct oral anticoagulant*") OR ALL=("NOAC*") OR ALL=("DOAC*") OR ALL=("hirudin") OR ALL=("desirudin") OR ALL=("lepirudin") OR ALL=("bivalirudin") OR ALL=("argatroban") OR ALL=("Vitamin K Antagonist*") OR ALL=("VKA") OR ALL=("warfarin") OR ALL=("acenocoumarol") OR ALL=("phenprocoumon") OR ALL=("aspirin") OR ALL=("acetylsalicylic") OR ALL=("dipyridamole") OR ALL=("clopidogrel") OR ALL=("prasugrel") OR ALL=("ticagrelor") OR ALL=("cangrelor") OR ALL=("ticlopidine") OR ALL=("vorapaxar") OR ALL=("cilostazol") OR ALL=("glycoprotein IIb/IIIa receptor blocker*") OR ALL=("abciximab") OR ALL=("eptifibatide") OR ALL=("tirofiban") OR ALL=("bridging anticoagulation") OR ALL=("bridging anticoagulant")**)**

**Supplementary Table 7:** Search strategy for Question 2 - Should kidney transplant recipients receive prophylaxis for venous thrombosis after surgery?

**SEARCH STRATEGY FOR MEDLINE via PubMed**

A. **SEARCH SYNTAX**

1. kidney transplantation [MeSH Terms]
2. kidney transplantation [All Fields]
3. renal transplantation [All Fields]
4. kidney transplant recipient* [All Fields]
5. renal transplant recipient* [All Fields]
6. kidney transplant patient* [All Fields]
7. renal transplant patient* [All Fields]
8. kidney transplant candidate* [All Fields]
9. renal transplant candidate* [All Fields]
10. kidney graft [All Fields]
11. renal graft [All Fields]
12. kidney allograft [All Fields]
13. renal allograft [All Fields]
14. kidney grafting [All Fields]
15. renal grafting [All Fields]
16. #1 OR #2 OR #3 OR #4 OR #5 OR #6 OR #7 OR #8 OR #9 OR #10 OR #11 OR #12 OR #13 OR #14 OR #15
17. anticoagulant [All Fields]
18. anticoagulants [MeSH Terms]
19. anticoagulation [All Fields]
20. thromboprophylactic [All Fields]
21. thromboprophylaxis [All Fields]
22. thrombosis prophylaxis [All Fields]
23. thrombosis prevention [All Fields]
24. thromboembolism prophylaxis [All Fields]
25. thromboembolism prevention [All Fields]
26. VTE prophylaxis [All Fields]
27. VTE prevention [All Fields]
28. DVT prophylaxis [All Fields]
29. DVT prevention [All Fields]
30. heparin [All Fields]
31. unfractionated heparin [All Fields]
32. heparin [MeSH Terms]
33. low molecular weight heparin [All Fields]
34. Heparin, Low-Molecular-Weight [MeSH Terms]
35. LMWH [All Fields]
36. bemiparin [All Fields]
37. bemiparin [Supplementary concept]
38. dalteparin [All Fields]
39. dalteparin [MeSH Terms]
40. enoxaparin [All Fields]
41. enoxaparin [MeSH Terms]
42. tinzaparin [All Fields]
43. tinzaparin [MeSH Terms]
44. nadroparin [All Fields]
45. nadroparin [MeSH Terms]
46. fondaparinux [All Fields]
47. fondaparinux [MeSH Terms]
48. parnaparin [All Fields]
49. parnaparin [Supplementary concept]
50. reviparin [All Fields]
51. reviparin [Supplementary concept]
52. certoparin [All Fields]
53. certoparin [Supplementary concept]
54. Antithrombin [All Fields]
55. Antithrombins [MeSH Terms]
56. direct thrombin inhibitor* [All Fields]
57. Xa inhibitor* [All Fields]
58. Factor Xa Inhibitors [MeSH Terms]
59. IIa inhibitor*
60. dabigatran [All Fields]
61. dabigatran [MeSH Terms]
62. rivaroxaban [All Fields]
63. rivaroxaban [MeSH Terms]
64. apixaban [All Fields]
65. apixaban [Supplementary Concept]
66. edoxaban [All Fields]
67. edoxaban [Supplementary Concept]
68. novel oral anticoagulant* [All Fields]
69. direct oral anticoagulant* [All Fields]
70. NOAC* [All Fields]
71. DOAC* [All Fields]
72. bridging anticoagulation [All Fields]
73. bridging anticoagulant [All Fields]
74. #17 OR #18 OR #19 OR #20 OR #21 OR #22 OR #23 OR #24 OR #25 OR #26 OR #27 OR #28 OR #29 OR #30 OR #31 OR #32 OR #33 OR #34 OR #35 OR #36 OR #37 OR #38 OR #39 OR #40 OR #41 OR #42 OR #43 OR #44 OR #45 OR #46 OR #47 OR #48 OR #49 OR #50 OR #51 OR #52 OR #53 OR #54 OR #55 OR #56 OR #57 OR #58 OR #59 OR #60 OR #61 OR #62 OR #63 OR #64 OR #65 OR #66 OR #67 OR #68 OR #69 OR #70 OR #71 OR #72 OR #73
75. #16 AND #74

B. **SEARCH STRING**

**(**"kidney transplantation"[MeSH Terms] OR "kidney transplantation"[All Fields] OR "renal transplantation"[All Fields] OR "kidney transplant recipient*"[All Fields] OR "renal transplant recipient*"[All Fields] OR "kidney transplant patient*"[All Fields] OR "renal transplant patient*"[All Fields] OR "kidney transplant candidate*"[All Fields] OR "renal transplant candidate*"[All Fields] OR "kidney graft"[All Fields] OR "renal graft"[All Fields] OR "kidney allograft"[All Fields] OR "renal allograft"[All Fields] OR "kidney grafting"[All Fields] OR "renal grafting"[All Fields]**)** **AND (**"anticoagulant"[All Fields] OR "anticoagulants"[MeSH Terms] OR "anticoagulation"[All Fields] OR "thromboprophylactic"[All Fields] OR "thromboprophylaxis"[All Fields] OR "thrombosis prophylaxis"[All Fields] OR "thrombosis prevention"[All Fields] OR "thromboembolism prophylaxis"[All Fields] OR "thromboembolism prevention"[All Fields] OR "VTE prophylaxis"[All Fields] OR "VTE prevention"[All Fields] OR "DVT prophylaxis"[All Fields] OR "DVT prevention"[All Fields] OR "heparin"[All Fields] OR "unfractionated heparin"[All Fields] OR "heparin"[MeSH Terms] OR "low molecular weight heparin"[All Fields] OR "Heparin, Low-Molecular-Weight"[MeSH Terms] OR "LMWH"[All Fields] OR "bemiparin"[All Fields] OR "bemiparin"[Supplementary concept] OR "dalteparin"[All Fields] OR "dalteparin"[MeSH Terms] OR "enoxaparin"[All Fields] OR "enoxaparin"[MeSH Terms] OR "tinzaparin"[All Fields] OR "tinzaparin"[MeSH Terms] OR "nadroparin"[All Fields] OR "nadroparin"[MeSH Terms] OR "fondaparinux"[All Fields] OR "fondaparinux"[MeSH Terms] OR "parnaparin"[All Fields] OR "parnaparin"[Supplementary concept] OR "reviparin"[All Fields] OR "reviparin"[Supplementary concept] OR "certoparin"[All Fields] OR "certoparin"[Supplementary concept] OR "Antithrombin"[All Fields] OR "Antithrombins"[MeSH Terms] OR "direct thrombin inhibitor*"[All Fields] OR "Xa inhibitor*"[All Fields] OR "Factor Xa Inhibitors"[MeSH Terms] OR "IIa inhibitor*"[All Fields] OR "dabigatran"[All Fields] OR "dabigatran"[MeSH Terms] OR "rivaroxaban"[All Fields] OR "rivaroxaban"[MeSH Terms] OR "apixaban"[All Fields] OR "apixaban"[Supplementary Concept] OR "edoxaban"[All Fields] OR "edoxaban"[ Supplementary Concept] OR "novel oral anticoagulant*"[All Fields] OR "direct oral anticoagulant*"[All Fields] OR "NOAC*"[All Fields] OR "DOAC*"[All Fields] OR "bridging anticoagulation"[All Fields] OR "bridging anticoagulant"[All Fields]**)**

**SEARCH STRATEGY FOR COCHRANE LIBRARY**

A. **SEARCH SYNTAX**

1. MeSH descriptor: [Kidney Transplantation] explode all trees
2. kidney transplantation
3. renal transplantation
4. kidney transplant recipient*
5. renal transplant recipient*
6. kidney transplant patient*
7. renal transplant patient*
8. kidney transplant candidate*
9. renal transplant candidate*
10. kidney graft
11. renal graft
12. kidney allograft
13. renal allograft
14. kidney grafting
15. renal grafting
16. #1 OR #2 OR #3 OR #4 OR #5 OR #6 OR #7 OR #8 OR #9 OR #10 OR #11 OR #12 OR #13 OR #14 OR #15
17. anticoagulant*
18. MeSH descriptor: [Anticoagulants] explode all trees
19. anticoagulation
20. thromboprophylactic
21. thromboprophylaxis
22. thrombosis prophylaxis
23. thrombosis prevention
24. thromboembolism prophylaxis
25. thromboembolism prevention
26. VTE prophylaxis
27. VTE prevention
28. DVT prophylaxis
29. DVT prevention
30. heparin
31. unfractionated heparin
32. MeSH descriptor: [Heparin] explode all trees
33. low molecular weight heparin
34. LMWH
35. MeSH descriptor: [Heparin, Low-Molecular-Weight] explode all trees
36. bemiparin
37. dalteparin
38. MeSH descriptor: [Dalteparin] explode all trees
39. enoxaparin
40. MeSH descriptor: [Enoxaparin] explode all trees
41. tinzaparin
42. MeSH descriptor: [Tinzaparin] explode all trees
43. nadroparin
44. MeSH descriptor: [Nadroparin] explode all trees
45. fondaparinux
46. MeSH descriptor: [Fondaparinux] explode all trees
47. parnaparin
48. reviparin
49. certoparin
50. Antithrombin
51. MeSH descriptor: [Antithrombins] explode all trees
52. direct thrombin inhibitor*
53. Xa inhibitor*
54. MeSH descriptor: [Factor Xa Inhibitors] explode all trees
55. IIa inhibitor*
56. Dabigatran
57. MeSH descriptor: [Dabigatran] explode all trees
58. Rivaroxaban
59. MeSH descriptor: [Rivaroxaban] explode all trees
60. apixaban
61. edoxaban
62. novel oral anticoagulant*
63. direct oral anticoagulant*
64. NOAC*
65. DOAC*
66. bridging anticoagulation
67. bridging anticoagulant*
68. #17 OR #18 OR #19 OR #20 OR #21 OR #22 OR #23 OR #24 OR #25 OR #26 OR #27 OR #28 OR #29 OR #30 OR #31 OR #32 OR #33 OR #34 OR #35 OR #36 OR #37 OR #38 OR #39 OR #40 OR #41 OR #42 OR #43 OR #44 OR #45 OR #46 OR #47 OR #48 OR #49 OR #50 OR #51 OR #52 OR #53 OR #54 OR #55 OR #56 OR #57 OR #58 OR #59 OR #60 OR #61 OR #62 OR #63 OR #64 OR #65 OR #66 OR #67
69. #16 AND #68

B. **SEARCH STRING**

**(**(MeSH descriptor: [Kidney Transplantation] explode all trees) OR (kidney transplantation) OR (renal transplantation) OR (kidney transplant recipient*) OR (renal transplant recipient*) OR (kidney transplant patient*) OR (renal transplant patient*) OR (kidney transplant candidate*) OR (renal transplant candidate*) OR (kidney graft) OR (renal graft) OR (kidney allograft) OR (renal allograft) OR (kidney grafting) OR (renal grafting)**) AND (**(anticoagulant*) OR (MeSH descriptor: [Anticoagulants] explode all trees) OR (anticoagulation) OR (thromboprophylactic) OR (thromboprophylaxis) OR (thrombosis prophylaxis) OR (thrombosis prevention) OR (thromboembolism prophylaxis) OR (thromboembolism prevention) OR (VTE prophylaxis) OR (VTE prevention) OR (DVT prophylaxis) OR (DVT prevention) OR (heparin) OR (unfractionated heparin) OR (MeSH descriptor: [Heparin] explode all trees) OR (low molecular weight heparin) OR (LMWH) OR (MeSH descriptor: [Heparin, Low-Molecular-Weight] explode all trees) OR (bemiparin) OR (dalteparin) OR (MeSH descriptor: [Dalteparin] explode all trees) OR (enoxaparin) OR (MeSH descriptor: [Enoxaparin] explode all trees) OR (tinzaparin) OR (MeSH descriptor: [Tinzaparin] explode all trees) OR (nadroparin) OR (MeSH descriptor: [Nadroparin] explode all trees) OR (fondaparinux) OR (MeSH descriptor: [Fondaparinux] explode all trees) OR (parnaparin) OR (reviparin) OR (certoparin) OR (Antithrombin) OR (MeSH descriptor: [Antithrombins] explode all trees) OR (direct thrombin inhibitor*) OR (Xa inhibitor*) OR (MeSH descriptor: [Factor Xa Inhibitors] explode all trees) OR (IIa inhibitor*) OR (Dabigatran) OR (MeSH descriptor: [Dabigatran] explode all trees) OR (Rivaroxaban) OR (MeSH descriptor: [Rivaroxaban] explode all trees) OR (apixaban) OR (edoxaban) OR (novel oral anticoagulant*) OR (direct oral anticoagulant*) OR (NOAC*) OR (DOAC*) OR (bridging anticoagulation) OR (bridging anticoagulant*)**)**

**SEARCH STRATEGY FOR WEB OF SCIENCE**

A. **SEARCH SYNTAX**

1. ALL=("kidney transplantation")
2. ALL=("renal transplantation")
3. ALL=("kidney transplant recipient*")
4. ALL=("renal transplant recipient*")
5. ALL=("kidney transplant patient*")
6. ALL=("renal transplant patient*")
7. ALL=("kidney transplant candidate*")
8. ALL=("renal transplant candidate*")
9. ALL=("kidney graft")
10. ALL=("renal graft")
11. ALL=("kidney allograft")
12. ALL=("renal allograft")
13. ALL=("kidney grafting")
14. ALL=("renal grafting"
15. #1 OR #2 OR #3 OR #4 OR #5 OR #6 OR #7 OR #8 OR #9 OR #10 OR #11 OR #12 OR #13 OR #14 OR #15
16. ALL=("anticoagulant*")
17. ALL=("anticoagulation")
18. ALL=("thromboprophylactic")
19. ALL=("thromboprophylaxis")
20. ALL=("thrombosis prophylaxis")
21. ALL=("thrombosis prevention")
22. ALL=("thromboembolism prophylaxis")
23. ALL=("thromboembolism prevention")
24. ALL=("VTE prophylaxis")
25. ALL=("VTE prevention")
26. ALL=("DVT prophylaxis")
27. ALL=("DVT prevention")
28. ALL=("heparin")
29. ALL=("unfractionated heparin")
30. ALL=("low molecular weight heparin")
31. ALL=("LMWH")
32. ALL=("bemiparin")
33. ALL=("dalteparin")
34. ALL=("enoxaparin")
35. ALL=("tinzaparin")
36. ALL=("nadroparin")
37. ALL=("fondaparinux")
38. ALL=("parnaparin")
39. ALL=("reviparin")
40. ALL=("certoparin")
41. ALL=("Antithrombin")
42. ALL=("direct thrombin inhibitor*")
43. ALL=("Xa inhibitor*")
44. ALL=("Factor Xa Inhibitors")
45. ALL=("IIa inhibitor*")
46. ALL=("dabigatran")
47. ALL=("rivaroxaban")
48. ALL=("apixaban")
49. ALL=("edoxaban")
50. ALL=("novel oral anticoagulant*")
51. ALL=("direct oral anticoagulant*")
52. ALL=("NOAC*")
53. ALL=("DOAC*")
54. ALL=("bridging anticoagulation")
55. ALL=("bridging anticoagulant")
56. #16 OR #17 OR #18 OR #19 OR #20 OR #21 OR #22 OR #23 OR #24 OR #25 OR #26 OR #27 OR #28 OR #29 OR #30 OR #31 OR #32 OR #33 OR #34 OR #35 OR #36 OR #37 OR #38 OR #39 OR #40 OR #41 OR #42 OR #43 OR #44 OR #45 OR #46 OR #47 OR #48 OR #49 OR #50 OR #51 OR #52 OR #53 OR #54 OR #55
57. #15 AND #56

B. **SEARCH STRING**

**(**ALL=("kidney transplantation") OR ALL=("renal transplantation") OR ALL=("kidney transplant recipient*") OR ALL=("renal transplant recipient*") OR ALL=("kidney transplant patient*") OR ALL=("renal transplant patient*") OR ALL=("kidney transplant candidate*") OR ALL=("renal transplant candidate*") OR ALL=("kidney graft") OR ALL=("renal graft") OR ALL=("kidney allograft") OR ALL=("renal allograft") OR ALL=("kidney grafting") OR ALL=("renal grafting")**)** **AND** **(**ALL=("anticoagulant*") OR ALL=("anticoagulation") OR ALL=("thromboprophylactic") OR ALL=("thromboprophylaxis") OR ALL=("thrombosis prophylaxis") OR ALL=("thrombosis prevention") OR ALL=("thromboembolism prophylaxis") OR ALL=("thromboembolism prevention") OR ALL=("VTE prophylaxis") OR ALL=("VTE prevention") OR ALL=("DVT prophylaxis") OR ALL=("DVT prevention") OR ALL=("heparin") OR ALL=("unfractionated heparin") OR ALL=("low molecular weight heparin") OR ALL=("LMWH") OR ALL=("bemiparin") OR ALL=("dalteparin") OR ALL=("enoxaparin") OR ALL=("tinzaparin") OR ALL=("nadroparin") OR ALL=("fondaparinux") OR ALL=("parnaparin") OR ALL=("reviparin") OR ALL=("certoparin") OR ALL=("Antithrombin") OR ALL=("direct thrombin inhibitor*") OR ALL=("Xa inhibitor*") OR ALL=("Factor Xa Inhibitors") OR ALL=("IIa inhibitor*") OR ALL=("dabigatran") OR ALL=("rivaroxaban") OR ALL=("apixaban") OR ALL=("edoxaban") OR ALL=("novel oral anticoagulant*") OR ALL=("direct oral anticoagulant*") OR ALL=("NOAC*") OR ALL=("DOAC*") OR ALL=("bridging anticoagulation") OR ALL=("bridging anticoagulant")**)**

**Supplementary Table 8:** Search strategy for Question 3 - Can we use direct-acting oral anticoagulants (DOACs) in kidney transplant recipients in terms of safety (dosing, drug- drug interactions, adverse events)?

**SEARCH STRATEGY FOR MEDLINE via PubMed**

A. **SEARCH SYNTAX**

1. kidney transplantation [MeSH Terms]
2. kidney transplantation [All Fields]
3. renal transplantation [All Fields]
4. kidney transplant recipient* [All Fields]
5. renal transplant recipient* [All Fields]
6. kidney transplant patient* [All Fields]
7. renal transplant patient* [All Fields]
8. kidney transplant candidate* [All Fields]
9. renal transplant candidate* [All Fields]
10. kidney graft [All Fields]
11. renal graft [All Fields]
12. kidney allograft [All Fields]
13. renal allograft [All Fields]
14. kidney grafting [All Fields]
15. renal grafting [All Fields]
16. waiting list [All Fields]
17. #1 OR #2 OR #3 OR #4 OR #5 OR #6 OR #7 OR #8 OR #9 OR #10 OR #11 OR #12 OR #13 OR #14 OR #15 OR #16
18. Antithrombin [All Fields]
19. Antithrombins [MeSH Terms]
20. direct thrombin inhibitor* [All Fields]
21. Xa inhibitor* [All Fields]
22. Factor Xa Inhibitors [MeSH Terms]
23. IIa inhibitor*
24. dabigatran [All Fields]
25. dabigatran [MeSH Terms]
26. rivaroxaban [All Fields]
27. rivaroxaban [MeSH Terms]
28. apixaban [All Fields]
29. apixaban [Supplementary Concept]
30. edoxaban [All Fields]
31. edoxaban [Supplementary Concept]
32. betrixaban [All Fields]
33. betrixaban [Supplementary Concept]
34. novel oral anticoagulant* [All Fields]
35. direct oral anticoagulant* [All Fields]
36. NOAC* [All Fields]
37. DOAC* [All Fields]
38. Non-Vitamin K dependent anticoagulant* [All Fields]
39. DOAC* revers* [All Fields]
40. idarucizumab [All Fields]
41. idarucizumab [Supplementary Concept]
42. Andexanet alfa [All Fields]
43. PRT064445 [Supplementary Concept]
44. #18 OR #19 OR #20 OR #21 OR #22 OR #23 OR #24 OR #25 OR #26 OR #27 OR #28 OR #29 OR #30 OR #31 OR #32 OR #33 OR #34 OR #35 OR #36 OR #37 OR #38 OR #39 OR #40 OR #41 OR #42 OR #43
45. #17 AND #44

B. **SEARCH STRING**

**(**"kidney transplantation"[MeSH Terms] OR "kidney transplantation"[All Fields] OR "renal transplantation"[All Fields] OR "kidney transplant recipient*"[All Fields] OR "renal transplant recipient*"[All Fields] OR "kidney transplant patient*"[All Fields] OR "renal transplant patient*"[All Fields] OR "kidney transplant candidate*"[All Fields] OR "renal transplant candidate*"[All Fields] OR "kidney graft"[All Fields] OR "renal graft"[All Fields] OR "kidney allograft"[All Fields] OR "renal allograft"[All Fields] OR "kidney grafting"[All Fields] OR "renal grafting"[All Fields] OR "waiting list"[All Fields]**)** **AND (**"Antithrombin"[All Fields] OR "Antithrombins"[MeSH Terms] OR "direct thrombin inhibitor*"[All Fields] OR "Xa inhibitor*"[All Fields] OR "Factor Xa Inhibitors"[MeSH Terms] OR "IIa inhibitor*"[All Fields] OR "dabigatran"[All Fields] OR "dabigatran"[MeSH Terms] OR "rivaroxaban"[All Fields] OR "rivaroxaban"[MeSH Terms] OR "apixaban"[All Fields] OR "apixaban"[Supplementary Concept] OR "edoxaban"[All Fields] OR "edoxaban"[ Supplementary Concept] OR "betrixaban"[All Fields] or "betrixaban"[Supplementary Concept] OR "novel oral anticoagulant*"[All Fields] OR "direct oral anticoagulant*"[All Fields] OR "NOAC*"[All Fields] OR "DOAC*"[All Fields] OR "Non-Vitamin K dependent anticoagulant*"[All Fields] OR "DOAC* reversal"[All Fields] OR "idarucizumab"[All Fields] OR "idarucizumab"[Supplementary Concept] OR "andexanet alfa"[All Fields] OR "PRT064445"[Supplementary Concept]**)**

**SEARCH STRATEGY FOR COCHRANE LIBRARY**

A. **SEARCH SYNTAX**

1. MeSH descriptor: [Kidney Transplantation] explode all trees
2. kidney transplantation
3. renal transplantation
4. kidney transplant recipient*
5. renal transplant recipient*
6. kidney transplant patient*
7. renal transplant patient*
8. kidney transplant candidate*
9. renal transplant candidate*
10. kidney graft
11. renal graft
12. kidney allograft
13. renal allograft
14. kidney grafting
15. renal grafting
16. waiting list
17. #1 OR #2 OR #3 OR #4 OR #5 OR #6 OR #7 OR #8 OR #9 OR #10 OR #11 OR #12 OR #13 OR #14 OR #15 OR #16
18. Antithrombin
19. MeSH descriptor: [Antithrombins] explode all trees
20. direct thrombin inhibitor*
21. Xa inhibitor*
22. MeSH descriptor: [Factor Xa Inhibitors] explode all trees
23. IIa inhibitor*
24. dabigatran
25. MeSH descriptor: [Dabigatran] explode all trees
26. Rivaroxaban
27. MeSH descriptor: [Rivaroxaban] explode all trees
28. apixaban
29. edoxaban
30. betrixaban
31. novel oral anticoagulant*
32. direct oral anticoagulant*
33. NOAC*
34. DOAC*
35. Non-Vitamin K dependent anticoagulant*
36. DOAC* revers*
37. Idarucizumab
38. Andexanet alfa
39. #18 OR #19 OR #20 OR #21 OR #22 OR #23 OR #24 OR #25 OR #26 OR #27 OR #28 OR #29 OR #30 OR #31 OR #32 OR #33 OR #34 OR #35 OR #36 OR #37 OR #38
40. #17 AND #39

B. **SEARCH STRING**

**(**(MeSH descriptor: [Kidney Transplantation] explode all trees) OR (kidney transplantation) OR (renal transplantation) OR (kidney transplant recipient*) OR (renal transplant recipient*) OR (kidney transplant patient*) OR (renal transplant patient*) OR (kidney transplant candidate*) OR (renal transplant candidate*) OR (kidney graft) OR (renal graft) OR (kidney allograft) OR (renal allograft) OR (kidney grafting) OR (renal grafting) OR (waiting list)**) AND (**(Antithrombin) OR (MeSH descriptor: [Antithrombins] explode all trees) OR (direct thrombin inhibitor*) OR (Xa inhibitor*) OR (MeSH descriptor: [Factor Xa Inhibitors] explode all trees) OR (IIa inhibitor*) OR (Dabigatran) OR (MeSH descriptor: [Dabigatran] explode all trees) OR (Rivaroxaban) OR (MeSH descriptor: [Rivaroxaban] explode all trees) OR (apixaban) OR (edoxaban) OR (betrixaban) OR (novel oral anticoagulant*) OR (direct oral anticoagulant*) OR (NOAC*) OR (DOAC*) OR (Non-Vitamin K dependent anticoagulant*) OR (DOAC* revers*) OR (idarucizumab) OR (andexanet alfa)**)**

**SEARCH STRATEGY FOR WEB OF SCIENCE**

A. **SEARCH SYNTAX**

1. ALL=("kidney transplantation")
2. ALL=("renal transplantation")
3. ALL=("kidney transplant recipient*")
4. ALL=("renal transplant recipient*")
5. ALL=("kidney transplant patient*")
6. ALL=("renal transplant patient*")
7. ALL=("kidney transplant candidate*")
8. ALL=("renal transplant candidate*")
9. ALL=("kidney graft")
10. ALL=("renal graft")
11. ALL=("kidney allograft")
12. ALL=("renal allograft")
13. ALL=("kidney grafting")
14. ALL=("renal grafting")
15. ALL==("waiting list")
16. #1 OR #2 OR #3 OR #4 OR #5 OR #6 OR #7 OR #8 OR #9 OR #10 OR #11 OR #12 OR #13 OR #14 OR #15 OR
17. ALL=("Antithrombin")
18. ALL=("direct thrombin inhibitor*")
19. ALL=("Xa inhibitor*")
20. ALL=("Factor Xa Inhibitors")
21. ALL=("IIa inhibitor*")
22. ALL=("dabigatran")
23. ALL=("rivaroxaban")
24. ALL=("apixaban")
25. ALL=("edoxaban")
26. ALL=("betrixaban")
27. ALL=("novel oral anticoagulant*")
28. ALL=("direct oral anticoagulant*")
29. ALL=("NOAC*")
30. ALL=("DOAC*")
31. ALL=("Non-Vitamin K dependent anticoagulant*")
32. ALL=("DOAC* revers*")
33. ALL=("idarucizumab")
34. ALL=("andexanet alfa")
35. #17 OR #18 OR #19 OR #20 OR #21 OR #22 OR #23 OR #24 OR #25 OR #26 OR #27 OR #28 OR #29 OR #30 OR #31 OR #32 OR #33 OR #34
36. #16 AND #35

B. **SEARCH STRING**

**(**ALL=("kidney transplantation") OR ALL=("renal transplantation") OR ALL=("kidney transplant recipient*") OR ALL=("renal transplant recipient*") OR ALL=("kidney transplant patient*") OR ALL=("renal transplant patient*") OR ALL=("kidney transplant candidate*") OR ALL=("renal transplant candidate*") OR ALL=("kidney graft") OR ALL=("renal graft") OR ALL=("kidney allograft") OR ALL=("renal allograft") OR ALL=("kidney grafting") OR ALL=("renal grafting") OR ALL=("waiting list")**)** **AND** **(**ALL=("Antithrombin") OR ALL=("direct thrombin inhibitor*") OR ALL=("Xa inhibitor*") OR ALL=("Factor Xa Inhibitors") OR ALL=("IIa inhibitor*") OR ALL=("dabigatran") OR ALL=("rivaroxaban") OR ALL=("apixaban") OR ALL=("edoxaban") OR ALL=("betrixaban") OR ALL=("novel oral anticoagulant*") OR ALL=("direct oral anticoagulant*") OR ALL=("NOAC*") OR ALL=("DOAC*") OR ALL=("Non-Vitamin K dependent anticoagulant*") OR ALL=("DOAC* reversal") OR ALL=("idarucizumab") OR ALL=("andexanet alfa")**)**

**Supplementary Table 9:** Search strategy for Question 4 - Is there a need for interruption of antiplatelet therapy before graft biopsy?

**SEARCH STRATEGY FOR MEDLINE via PubMed**

A. **SEARCH SYNTAX**

1. ALL=("kidney transplantation")
2. ALL=("renal transplantation")
3. ALL=("kidney transplant recipient*")
4. ALL=("renal transplant recipient*")
5. ALL=("kidney transplant patient*")
6. ALL=("renal transplant patient*")
7. ALL=("kidney transplant candidate*")
8. ALL=("renal transplant candidate*")
9. ALL=("kidney graft")
10. ALL=("renal graft")
11. ALL=("kidney allograft")
12. ALL=("renal allograft")
13. ALL=("kidney grafting")
14. ALL=("renal grafting"
15. #1 OR #2 OR #3 OR #4 OR #5 OR #6 OR #7 OR #8 OR #9 OR #10 OR #11 OR #12 OR #13 OR #14 OR #15
16. ALL=("antithrombotic")
17. ALL=("antiplatelet")
18. ALL=("platelet aggregation inhibitors")
19. ALL=("anticoagulant*")
20. ALL=("anticoagulation")
21. ALL=("thromboprophylactic")
22. ALL=("thromboprophylaxis")
23. ALL=("thrombosis prophylaxis")
24. ALL=("thrombosis prevention")
25. ALL=("thromboembolism prophylaxis")
26. ALL=("thromboembolism prevention")
27. ALL=("VTE prophylaxis")
28. ALL=("VTE prevention")
29. ALL=("DVT prophylaxis")
30. ALL=("DVT prevention")
31. ALL=("heparin")
32. ALL=("unfractionated heparin")
33. ALL=("low molecular weight heparin")
34. ALL=("LMWH")
35. ALL=("bemiparin")
36. ALL=("dalteparin")
37. ALL=("enoxaparin")
38. ALL=("tinzaparin")
39. ALL=("nadroparin")
40. ALL=("fondaparinux")
41. ALL=("parnaparin")
42. ALL=("reviparin")
43. ALL=("certoparin")
44. ALL=("Antithrombin")
45. ALL=("direct thrombin inhibitor*")
46. ALL=("Xa inhibitor*")
47. ALL=("Factor Xa Inhibitors")
48. ALL=("IIa inhibitor*")
49. ALL=("dabigatran")
50. ALL=("rivaroxaban")
51. ALL=("apixaban")
52. ALL=("edoxaban")
53. ALL=("novel oral anticoagulant*")
54. ALL=("direct oral anticoagulant*")
55. ALL=("NOAC*")
56. ALL=("DOAC*")
57. ALL=("hirudin")
58. ALL=("desirudin")
59. ALL=("lepirudin")
60. ALL=("bivalirudin")
61. ALL=("argatroban")
62. ALL=("Vitamin K Antagonist*")
63. ALL=("VKA")
64. ALL=("warfarin")
65. ALL=("acenocoumarol")
66. ALL=("phenprocoumon")
67. ALL=("aspirin")
68. ALL=("acetylsalicylic")
69. ALL=("dipyridamole")
70. ALL=("clopidogrel")
71. ALL=("prasugrel")
72. ALL=("ticagrelor")
73. ALL=("cangrelor")
74. ALL=("ticlopidine")
75. ALL=("vorapaxar")
76. ALL=("cilostazol")
77. ALL=("glycoprotein IIb/IIIa receptor blocker*")
78. ALL=("abciximab")
79. ALL=("eptifibatide")
80. ALL=("tirofiban")
81. ALL=("bridging anticoagulation")
82. ALL=("bridging anticoagulant")
83. #16 OR #17 OR #18 OR #19 OR #20 OR #21 OR #22 OR #23 OR #24 OR #25 OR #26 OR #27 OR #28 OR #29 OR #30 OR #31 OR #32 OR #33 OR #34 OR #35 OR #36 OR #37 OR #38 OR #39 OR #40 OR #41 OR #42 OR #43 OR #44 OR #45 OR #46 OR #47 OR #48 OR #49 OR #50 OR #51 OR #52 OR #53 OR #54 OR #55 OR #56 OR #57 OR #58 OR #59 OR #60 OR #61 OR #62 OR #63 OR #64 OR #65 OR #66 OR #67 OR #68 OR #69 OR #70 OR #71 OR #72 OR #73 OR #74 OR #75 OR #76 OR #77 OR #78 OR #79 OR #80 OR #81 OR #82
84. ALL=("biops*")
85. ALL=("graft biops*")
86. ALL=("allograft biopsy")
87. ALL=("true*cut")
88. ALL=("tru-cut")
89. ALL=("trucut")
90. ALL=("needle")
91. ALL=("fine needle")
92. #84 OR #85 OR #86 OR #87 OR #88 OR #89 OR #90 OR #91
93. #15 AND #83 AND #92

B. **SEARCH STRING**

**(**ALL=("kidney transplantation") OR ALL=("renal transplantation") OR ALL=("kidney transplant recipient*") OR ALL=("renal transplant recipient*") OR ALL=("kidney transplant patient*") OR ALL=("renal transplant patient*") OR ALL=("kidney transplant candidate*") OR ALL=("renal transplant candidate*") OR ALL=("kidney graft") OR ALL=("renal graft") OR ALL=("kidney allograft") OR ALL=("renal allograft") OR ALL=("kidney grafting") OR ALL=("renal grafting")**)** **AND** **(**ALL=("antithrombotic") OR ALL=("antiplatelet") OR ALL=("platelet aggregation inhibitors") OR ALL=("anticoagulant*") OR ALL=("anticoagulation") OR ALL=("thromboprophylactic") OR ALL=("thromboprophylaxis") OR ALL=("thrombosis prophylaxis") OR ALL=("thrombosis prevention") OR ALL=("thromboembolism prophylaxis") OR ALL=("thromboembolism prevention") OR ALL=("VTE prophylaxis") OR ALL=("VTE prevention") OR ALL=("DVT prophylaxis") OR ALL=("DVT prevention") OR ALL=("heparin") OR ALL=("unfractionated heparin") OR ALL=("low molecular weight heparin") OR ALL=("LMWH") OR ALL=("bemiparin") OR ALL=("dalteparin") OR ALL=("enoxaparin") OR ALL=("tinzaparin") OR ALL=("nadroparin") OR ALL=("fondaparinux") OR ALL=("parnaparin") OR ALL=("reviparin") OR ALL=("certoparin") OR ALL=("Antithrombin") OR ALL=("direct thrombin inhibitor*") OR ALL=("Xa inhibitor*") OR ALL=("Factor Xa Inhibitors") OR ALL=("IIa inhibitor*") OR ALL=("dabigatran") OR ALL=("rivaroxaban") OR ALL=("apixaban") OR ALL=("edoxaban") OR ALL=("novel oral anticoagulant*") OR ALL=("direct oral anticoagulant*") OR ALL=("NOAC*") OR ALL=("DOAC*") OR ALL=("hirudin") OR ALL=("desirudin") OR ALL=("lepirudin") OR ALL=("bivalirudin") OR ALL=("argatroban") OR ALL=("Vitamin K Antagonist*") OR ALL=("VKA") OR ALL=("warfarin") OR ALL=("acenocoumarol") OR ALL=("phenprocoumon") OR ALL=("aspirin") OR ALL=("acetylsalicylic") OR ALL=("dipyridamole") OR ALL=("clopidogrel") OR ALL=("prasugrel") OR ALL=("ticagrelor") OR ALL=("cangrelor") OR ALL=("ticlopidine") OR ALL=("vorapaxar") OR ALL=("cilostazol") OR ALL=("glycoprotein IIb/IIIa receptor blocker*") OR ALL=("abciximab") OR ALL=("eptifibatide") OR ALL=("tirofiban") OR ALL=("bridging anticoagulation") OR ALL=("bridging anticoagulant")**) AND (**ALL=("biops*") OR ALL=("graft biops*") OR ALL=("allograft biopsy") OR ALL=("true*cut") OR ALL=("tru-cut") OR ALL=("trucut") OR ALL=("needle") OR ALL=("fine needle")**)**

**SEARCH STRATEGY FOR COCHRANE LIBRARY**

A. **SEARCH SYNTAX**

1. MeSH descriptor: [Kidney Transplantation] explode all trees
2. kidney transplantation
3. renal transplantation
4. kidney transplant recipient*
5. renal transplant recipient*
6. kidney transplant patient*
7. renal transplant patient*
8. kidney transplant candidate*
9. renal transplant candidate*
10. kidney graft
11. renal graft
12. kidney allograft
13. renal allograft
14. kidney grafting
15. renal grafting
16. #1 OR #2 OR #3 OR #4 OR #5 OR #6 OR #7 OR #8 OR #9 OR #10 OR #11 OR #12 OR #13 OR #14 OR #15
17. antithrombotic*
18. antiplatelet*
19. MeSH descriptor: [platelet aggregation inhibitors] explode all trees
20. anticoagulant*
21. MeSH descriptor: [Anticoagulants] explode all trees
22. anticoagulation
23. thromboprophylactic
24. thromboprophylaxis
25. thrombosis prophylaxis
26. thrombosis prevention
27. thromboembolism prophylaxis
28. thromboembolism prevention
29. VTE prophylaxis
30. VTE prevention
31. DVT prophylaxis
32. DVT prevention
33. heparin
34. unfractionated heparin
35. MeSH descriptor: [Heparin] explode all trees
36. low molecular weight heparin
37. LMWH
38. MeSH descriptor: [Heparin, Low-Molecular-Weight] explode all trees
39. bemiparin
40. dalteparin
41. MeSH descriptor: [Dalteparin] explode all trees
42. enoxaparin
43. MeSH descriptor: [Enoxaparin] explode all trees
44. tinzaparin
45. MeSH descriptor: [Tinzaparin] explode all trees
46. nadroparin
47. MeSH descriptor: [Nadroparin] explode all trees
48. fondaparinux
49. MeSH descriptor: [Fondaparinux] explode all trees
50. parnaparin
51. reviparin
52. certoparin
53. Antithrombin
54. MeSH descriptor: [Antithrombins] explode all trees
55. direct thrombin inhibitor*
56. Xa inhibitor*
57. MeSH descriptor: [Factor Xa Inhibitors] explode all trees
58. IIa inhibitor*
59. Dabigatran
60. MeSH descriptor: [Dabigatran] explode all trees
61. Rivaroxaban
62. MeSH descriptor: [Rivaroxaban] explode all trees
63. apixaban
64. edoxaban
65. novel oral anticoagulant*
66. direct oral anticoagulant*
67. NOAC*
68. DOAC*
69. hirudin
70. MeSH descriptor: [Hirudins] explode all trees
71. desirudin
72. lepirudin
73. bivalirudin
74. argatroban
75. Vitamin K Antagonist*
76. VKA*
77. warfarin
78. MeSH descriptor: [Warfarin] explode all trees
79. acenocoumarol
80. MeSH descriptor: [Acenocoumarol] explode all trees
81. Phenprocoumon
82. MeSH descriptor: [Phenprocoumon] explode all trees
83. aspirin
84. acetylsalicylic
85. MeSH descriptor: [Aspirin] explode all trees
86. dipyridamole
87. MeSH descriptor: [Dipyridamole] explode all trees
88. clopidogrel
89. MeSH descriptor: [Clopidogrel] explode all trees
90. prasugrel
91. MeSH descriptor: [Prasugrel Hydrochloride] explode all trees
92. ticagrelor
93. MeSH descriptor: [Ticagrelor] explode all trees
94. cangrelor
95. ticlopidine
96. MeSH descriptor: [Ticlopidine] explode all trees
97. vorapaxar
98. cilostazol
99. MeSH descriptor: [Cilostazol] explode all trees
100. glycoprotein IIb IIIa receptor blocker*
101. abciximab
102. MeSH descriptor: [Abciximab] explode all trees
103. eptifibatide
104. MeSH descriptor: [Eptifibatide] explode all trees
105. tirofiban
106. MeSH descriptor: [Tirofiban] explode all trees
107. bridging anticoagulation
108. bridging anticoagulant*
109. #17 OR #18 OR #19 OR #20 OR #21 OR #22 OR #23 OR #24 OR #25 OR #26 OR #27 OR #28 OR #29 OR #30 OR #31 OR #32 OR #33 OR #34 OR #35 OR #36 OR #37 OR #38 OR #39 OR #40 OR #41 OR #42 OR #43 OR #44 OR #45 OR #46 OR #47 OR #48 OR #49 OR #50 OR #51 OR #52 OR #53 OR #54 OR #55 OR #56 OR #57 OR #58 OR #59 OR #60 OR #61 OR #62 OR #63 OR #64 OR #65 OR #66 OR #67 OR #68 OR #69 OR #70 OR #71 OR #72 OR #73 OR #74 OR #75 OR #76 OR #77 OR #78 OR #79 OR #80 OR #81 OR #82 OR #83 OR #84 OR #85 OR #86 OR #87 OR #88 OR #89 OR #90 OR #91 OR #92 OR #93 OR #94 OR #95 OR #96 OR #97 OR #98 OR #99 OR #100 OR #101 OR #102 OR #103 OR #104 OR #105 OR #106 OR #107 OR #108
110. biops*
111. MeSH descriptor: [Biopsy] explode all trees
112. graft biops*
113. allograft biops*
114. true*cut
115. tru-cut
116. trucut
117. needle
118. fine needle
119. #110 OR #111 OR #112 OR #113 OR #114 OR #115 OR #116 OR #117 OR #118
120. #16 AND #109 AND #119

B. **SEARCH STRING**

**(**(MeSH descriptor: [Kidney Transplantation] explode all trees) OR (kidney transplantation) OR (renal transplantation) OR (kidney transplant recipient*) OR (renal transplant recipient*) OR (kidney transplant patient*) OR (renal transplant patient*) OR (kidney transplant candidate*) OR (renal transplant candidate*) OR (kidney graft) OR (renal graft) OR (kidney allograft) OR (renal allograft) OR (kidney grafting) OR (renal grafting)**) AND (**(antithrombotic*) OR (antiplatelet*) OR (MeSH descriptor: [platelet aggregation inhibitors] explode all trees) OR (anticoagulant*) OR (MeSH descriptor: [Anticoagulants] explode all trees) OR (anticoagulation) OR (thromboprophylactic) OR (thromboprophylaxis) OR (thrombosis prophylaxis) OR (thrombosis prevention) OR (thromboembolism prophylaxis) OR (thromboembolism prevention) OR (VTE prophylaxis) OR (VTE prevention) OR (DVT prophylaxis) OR (DVT prevention) OR (heparin) OR (unfractionated heparin) OR (MeSH descriptor: [Heparin] explode all trees) OR (low molecular weight heparin) OR (LMWH) OR (MeSH descriptor: [Heparin, Low-Molecular-Weight] explode all trees) OR (bemiparin) OR (dalteparin) OR (MeSH descriptor: [Dalteparin] explode all trees) OR (enoxaparin) OR (MeSH descriptor: [Enoxaparin] explode all trees) OR (tinzaparin) OR (MeSH descriptor: [Tinzaparin] explode all trees) OR (nadroparin) OR (MeSH descriptor: [Nadroparin] explode all trees) OR (fondaparinux) OR (MeSH descriptor: [Fondaparinux] explode all trees) OR (parnaparin) OR (reviparin) OR (certoparin) OR (Antithrombin) OR (MeSH descriptor: [Antithrombins] explode all trees) OR (direct thrombin inhibitor*) OR (Xa inhibitor*) OR (MeSH descriptor: [Factor Xa Inhibitors] explode all trees) OR (IIa inhibitor*) OR (Dabigatran) OR (MeSH descriptor: [Dabigatran] explode all trees) OR (Rivaroxaban) OR (MeSH descriptor: [Rivaroxaban] explode all trees) OR (apixaban) OR (edoxaban) OR (novel oral anticoagulant*) OR (direct oral anticoagulant*) OR (NOAC*) OR (DOAC*) OR (hirudin) OR (MeSH descriptor: [Hirudins] explode all trees) OR (desirudin) OR (lepirudin) OR (bivalirudin) OR (argatroban) OR (Vitamin K Antagonist*) OR (VKA*) OR (warfarin) OR (MeSH descriptor: [Warfarin] explode all trees) OR (acenocoumarol) OR (MeSH descriptor: [Acenocoumarol] explode all trees) OR (Phenprocoumon) OR (MeSH descriptor: [Phenprocoumon] explode all trees) OR (aspirin) OR (acetylsalicylic) OR (MeSH descriptor: [Aspirin] explode all trees) OR (dipyridamole) OR (MeSH descriptor: [Dipyridamole] explode all trees) OR (clopidogrel) OR (MeSH descriptor: [Clopidogrel] explode all trees) OR (prasugrel) OR (MeSH descriptor: [Prasugrel Hydrochloride] explode all trees) OR (ticagrelor) OR (MeSH descriptor: [Ticagrelor] explode all trees) OR (cangrelor) OR (ticlopidine) OR (MeSH descriptor: [Ticlopidine] explode all trees) OR (vorapaxar) OR (cilostazol) OR (MeSH descriptor: [Cilostazol] explode all trees) OR (glycoprotein IIb IIIa receptor blocker*) OR (abciximab) OR (MeSH descriptor: [Abciximab] explode all trees) OR (eptifibatide) OR (MeSH descriptor: [Eptifibatide] explode all trees) OR (tirofiban) OR (MeSH descriptor: [Tirofiban] explode all trees) OR (bridging anticoagulation) OR (bridging anticoagulant*)**)**

**SEARCH STRATEGY FOR WEB OF SCIENCE**

A. **SEARCH SYNTAX**

1. ALL=("kidney transplantation")
2. ALL=("renal transplantation")
3. ALL=("kidney transplant recipient*")
4. ALL=("renal transplant recipient*")
5. ALL=("kidney transplant patient*")
6. ALL=("renal transplant patient*")
7. ALL=("kidney transplant candidate*")
8. ALL=("renal transplant candidate*")
9. ALL=("kidney graft")
10. ALL=("renal graft")
11. ALL=("kidney allograft")
12. ALL=("renal allograft")
13. ALL=("kidney grafting")
14. ALL=("renal grafting"
15. #1 OR #2 OR #3 OR #4 OR #5 OR #6 OR #7 OR #8 OR #9 OR #10 OR #11 OR #12 OR #13 OR #14 OR #15
16. ALL=("antithrombotic")
17. ALL=("antiplatelet")
18. ALL=("platelet aggregation inhibitors")
19. ALL=("anticoagulant*")
20. ALL=("anticoagulation")
21. ALL=("thromboprophylactic")
22. ALL=("thromboprophylaxis")
23. ALL=("thrombosis prophylaxis")
24. ALL=("thrombosis prevention")
25. ALL=("thromboembolism prophylaxis")
26. ALL=("thromboembolism prevention")
27. ALL=("VTE prophylaxis")
28. ALL=("VTE prevention")
29. ALL=("DVT prophylaxis")
30. ALL=("DVT prevention")
31. ALL=("heparin")
32. ALL=("unfractionated heparin")
33. ALL=("low molecular weight heparin")
34. ALL=("LMWH")
35. ALL=("bemiparin")
36. ALL=("dalteparin")
37. ALL=("enoxaparin")
38. ALL=("tinzaparin")
39. ALL=("nadroparin")
40. ALL=("fondaparinux")
41. ALL=("parnaparin")
42. ALL=("reviparin")
43. ALL=("certoparin")
44. ALL=("Antithrombin")
45. ALL=("direct thrombin inhibitor*")
46. ALL=("Xa inhibitor*")
47. ALL=("Factor Xa Inhibitors")
48. ALL=("IIa inhibitor*")
49. ALL=("dabigatran")
50. ALL=("rivaroxaban")
51. ALL=("apixaban")
52. ALL=("edoxaban")
53. ALL=("novel oral anticoagulant*")
54. ALL=("direct oral anticoagulant*")
55. ALL=("NOAC*")
56. ALL=("DOAC*")
57. ALL=("hirudin")
58. ALL=("desirudin")
59. ALL=("lepirudin")
60. ALL=("bivalirudin")
61. ALL=("argatroban")
62. ALL=("Vitamin K Antagonist*")
63. ALL=("VKA")
64. ALL=("warfarin")
65. ALL=("acenocoumarol")
66. ALL=("phenprocoumon")
67. ALL=("aspirin")
68. ALL=("acetylsalicylic")
69. ALL=("dipyridamole")
70. ALL=("clopidogrel")
71. ALL=("prasugrel")
72. ALL=("ticagrelor")
73. ALL=("cangrelor")
74. ALL=("ticlopidine")
75. ALL=("vorapaxar")
76. ALL=("cilostazol")
77. ALL=("glycoprotein IIb/IIIa receptor blocker*")
78. ALL=("abciximab")
79. ALL=("eptifibatide")
80. ALL=("tirofiban")
81. ALL=("bridging anticoagulation")
82. ALL=("bridging anticoagulant")
83. #16 OR #17 OR #18 OR #19 OR #20 OR #21 OR #22 OR #23 OR #24 OR #25 OR #26 OR #27 OR #28 OR #29 OR #30 OR #31 OR #32 OR #33 OR #34 OR #35 OR #36 OR #37 OR #38 OR #39 OR #40 OR #41 OR #42 OR #43 OR #44 OR #45 OR #46 OR #47 OR #48 OR #49 OR #50 OR #51 OR #52 OR #53 OR #54 OR #55 OR #56 OR #57 OR #58 OR #59 OR #60 OR #61 OR #62 OR #63 OR #64 OR #65 OR #66 OR #67 OR #68 OR #69 OR #70 OR #71 OR #72 OR #73 OR #74 OR #75 OR #76 OR #77 OR #78 OR #79 OR #80 OR #81 OR #82
84. ALL=("biops*")
85. ALL=("graft biops*")
86. ALL=("allograft biopsy")
87. ALL=("true*cut")
88. ALL=("tru-cut")
89. ALL=("trucut")
90. ALL=("needle")
91. ALL=("fine needle")
92. #84 OR #85 OR #86 OR #87 OR #88 OR #89 OR #90 OR #91
93. #15 AND #83 AND #92

B. **SEARCH STRING**

**(**ALL=("kidney transplantation") OR ALL=("renal transplantation") OR ALL=("kidney transplant recipient*") OR ALL=("renal transplant recipient*") OR ALL=("kidney transplant patient*") OR ALL=("renal transplant patient*") OR ALL=("kidney transplant candidate*") OR ALL=("renal transplant candidate*") OR ALL=("kidney graft") OR ALL=("renal graft") OR ALL=("kidney allograft") OR ALL=("renal allograft") OR ALL=("kidney grafting") OR ALL=("renal grafting")**)** **AND** **(**ALL=("antithrombotic") OR ALL=("antiplatelet") OR ALL=("platelet aggregation inhibitors") OR ALL=("anticoagulant*") OR ALL=("anticoagulation") OR ALL=("thromboprophylactic") OR ALL=("thromboprophylaxis") OR ALL=("thrombosis prophylaxis") OR ALL=("thrombosis prevention") OR ALL=("thromboembolism prophylaxis") OR ALL=("thromboembolism prevention") OR ALL=("VTE prophylaxis") OR ALL=("VTE prevention") OR ALL=("DVT prophylaxis") OR ALL=("DVT prevention") OR ALL=("heparin") OR ALL=("unfractionated heparin") OR ALL=("low molecular weight heparin") OR ALL=("LMWH") OR ALL=("bemiparin") OR ALL=("dalteparin") OR ALL=("enoxaparin") OR ALL=("tinzaparin") OR ALL=("nadroparin") OR ALL=("fondaparinux") OR ALL=("parnaparin") OR ALL=("reviparin") OR ALL=("certoparin") OR ALL=("Antithrombin") OR ALL=("direct thrombin inhibitor*") OR ALL=("Xa inhibitor*") OR ALL=("Factor Xa Inhibitors") OR ALL=("IIa inhibitor*") OR ALL=("dabigatran") OR ALL=("rivaroxaban") OR ALL=("apixaban") OR ALL=("edoxaban") OR ALL=("novel oral anticoagulant*") OR ALL=("direct oral anticoagulant*") OR ALL=("NOAC*") OR ALL=("DOAC*") OR ALL=("hirudin") OR ALL=("desirudin") OR ALL=("lepirudin") OR ALL=("bivalirudin") OR ALL=("argatroban") OR ALL=("Vitamin K Antagonist*") OR ALL=("VKA") OR ALL=("warfarin") OR ALL=("acenocoumarol") OR ALL=("phenprocoumon") OR ALL=("aspirin") OR ALL=("acetylsalicylic") OR ALL=("dipyridamole") OR ALL=("clopidogrel") OR ALL=("prasugrel") OR ALL=("ticagrelor") OR ALL=("cangrelor") OR ALL=("ticlopidine") OR ALL=("vorapaxar") OR ALL=("cilostazol") OR ALL=("glycoprotein IIb/IIIa receptor blocker*") OR ALL=("abciximab") OR ALL=("eptifibatide") OR ALL=("tirofiban") OR ALL=("bridging anticoagulation") OR ALL=("bridging anticoagulant")**) AND (**ALL=("biops*") OR ALL=("graft biops*") OR ALL=("allograft biopsy") OR ALL=("true*cut") OR ALL=("tru-cut") OR ALL=("trucut") OR ALL=("needle") OR ALL=("fine needle")**)**

# **3. STUDY SELECTION**

For each research question, all retrieved studies were imported into a reference management software for duplicate removal. After deduplication, two independent groups examined the titles and abstracts of retrieved records thoroughly to identify potentially eligible studies using the inclusion criteria as defined by the research question and PICO tables. Studies were excluded if they failed to cover the appropriate population (P), one of the interventions (I) and comparators (C) as well as comparisons as specified there. Outcomes (O) were not used as inclusion or exclusion criteria. Abstracts that did not meet the inclusion criteria were discarded. When multiple reports of the same study were identified, the one with the most adequate data was included in the systematic review. In a second step, full text and supplementary appendices of potentially relevant studies were examined for eligibility, according to pre-set eligibility criteria as listed above, in order to select the studies that would be finally included. Reference lists from the included publications were also thoroughly screened to identify additional papers. Any discrepancies or disagreements during the study selection process were resolved after discussion and group consensus. The flow diagrams depicting the study selection process for each research question are presented below.

**Supplementary Figure 1:** Study selection flow diagram for Question 1 - What is the peri-operative bleeding risk in kidney transplant candidates who are on antiplatelet or anticoagulant therapies?

**Identification of studies via databases, registers and other sources**

**Records identified** from:

**MEDLINE/PubMed** (n = 1,400)

**Cochrane Library** (n = 179)

**Web of Science** (n = 1,034)

**Records identified from**:

Reference lists (n = 19)

**Records screened after duplicates**

(n = 1,310)

**Records excluded based on title and abstract**

(n = 1,178)

**Full-text articles assessed for eligibility**

(n = 132)

**Records excluded**

No peri-operative intervention or exposure (n = 69)

Wrong population (n= 6)

Review articles (n = 7)

Other reasons (n = 25)

**Studies included** in qualitative and quantitative analysis

(n= 25)

**Duplicates removed**

(n = 1,322)

**Identification**

**Screening**

**Included**

**Eligibility**

**Supplementary Figure 2:** Study selection flow diagram for Question 2 - Should kidney transplant recipients receive prophylaxis for venous thrombosis after surgery?

**Identification of studies via databases, registers and other sources**

**Records identified** from:

**MEDLINE/PubMed** (n = 1,100)

**Cochrane Library** (n = 816)

**Web of Science** (n = 755)

**Records identified from**:

Reference lists (n = 1)

**Records screened after duplicates**

(n = 1,560)

**Records excluded based on title and abstract**

(n = 1,178)

**Full-text articles assessed for eligibility**

(n = 82)

**Records excluded**

No post-operative intervention or exposure (n = 40)

Wrong population (n= 12)

Review articles (n = 9)

Other reasons (n = 11)

**Studies included** in qualitative and quantitative analysis

(n= 10)

**Duplicates removed**

(n = 1,112)

**Identification**

**Screening**

**Included**

**Eligibility**

**Supplementary Figure 3:** Study selection flow diagram for Question 3 - Can we use direct-acting oral anticoagulants (DOACs) in kidney transplant recipients in terms of safety (dosing, drug- drug interactions, adverse events)?

**Identification of studies via databases, registers and other sources**

**Records identified** from:

**MEDLINE/PubMed** (n = 160)

**Cochrane Library** (n = 180)

**Web of Science** (n = 125)

**Records identified from**:

Reference lists (n = 0)

**Records screened after duplicates**

(n = 252)

**Records excluded based on title and abstract**

(n = 207)

**Full-text articles assessed for eligibility**

(n = 45)

**Records excluded**

Wrong population (n= 10)

Review articles (n = 5)

Other reasons (n = 15)

**Studies included** in qualitative and quantitative analysis

(n= 15)

**Duplicates removed**

(n = 213)

**Identification**

**Screening**

**Included**

**Eligibility**

**Supplementary Figure 4:** Study selection flow diagram for Question 4 - Is there a need for interruption of antiplatelet therapy before graft biopsy?

**Identification of studies via databases, registers and other sources**

**Records identified** from:

**MEDLINE/PubMed** (n = 127)

**Cochrane Library** (n = 116)

**Web of Science** (n = 79)

**Records identified from**:

Reference lists (n = 16)

**Records screened after duplicates**

(n = 208)

**Records excluded based on title and abstract**

(n = 171)

**Full-text articles assessed for eligibility**

(n = 37)

**Records excluded**

Wrong population (n= 19)

Wrong antithrombotic agent (n = 5)

Other reasons (n = 6)

**Studies included** in qualitative and quantitative analysis

(n= 7)

**Duplicates removed**

(n = 130)

**Identification**

**Screening**

**Included**

**Eligibility**

# **4. DATA EXTRACTION**

Data collection was performed with standardized spreadsheets designed in Microsoft Excel. The following data were extracted according to the Cochrane checklist of items: first author, year of publication, journal, study design, patients’ characteristics, number of patients, type and dosage of antithrombotic agents and comparator (if applicable), types and definitions of outcomes, follow-up/study period, measures of effect and estimates for all outcomes, risk of bias assessment. For each study, the previous data was extracted independently by two authors. Discrepancies were resolved after discussion and group consensus.

# **5. RISK OF BIAS ASSESSMENT**

The risk of bias was ascertained using well validated methods which included the Version 2 of the Cochrane risk-of-bias tool (RoB 2) for randomized controlled trials and the Newcastle Ottawa scale for cohort and case control studies.

# **6. QUALITY OF EVIDENCE**

The evidence for outcomes was evaluated using the Grading of Recommendations Assessment, Development and Evaluation (GRADE) tool. Studies were categorized into four levels of evidence quality (high, moderate, low or very low). According to study design, RCTs were deemed of high- and observational studies of low-quality rating. The quality of evidence was downgraded by one level if there was a serious risk of bias, indirectness, inconsistency, imprecision or publication bias; similarly, the evidence level was upgraded if there were studies with a large effect.

# **7. FORMULATING AND GRADING CLINICAL PRACTICE POINTS**

After the summary tables had been prepared and the evidence assessed, clinical practice points (rather than recommendations) were formulated. These clinical practice points were based on the available evidence and were unanimously agreed between all expert authors. Regarding the grading of these statements, given the low or very low certainty of evidence, mostly deriving from the observational nature of available studies and relevant data, authors decided to provide ungraded clinical practice points.

# **8. SUMMARY EVIDENCE TABLES**

**Supplementary Table 10:** Summary Evidence Table for Question 1 – What is the peri-operative bleeding risk in kidney transplant candidates who are on antiplatelet or anticoagulant therapies?

| **Author, year, Journal**  *(displayed in chronological order)* | | **Study design/Time frame** | **Patient characteristics** | **No. of patients** | **Antithrombotic drug and dosage** | **Comparator (if applicable)** | **Outcomes** | **Follow-up for the outcomes of interest** | **Bleeding events** | **Thrombotic events** | **Risk of bias** |
| --- | --- | --- | --- | --- | --- | --- | --- | --- | --- | --- | --- |
| **Antiplatelets** | | | | | | | | | | | |
| Robertson et al, 2000, Nephrol Dial Transplant | | Single centre retrospective cohort study (comparing two periods, i.e., 1985-1991 and 1991-1997) | Adult living and deceased KTRs | N=955 | **PERIOD 07/1991-06/1997**  (n= 480)   - low-dose aspirin (75mg od) for 1-month post-transplant (if high risk patients, i.e., with diabetes, history of DVT or PAD, for long-term) - high-dose aspirin (150mg od) for long-term (in patients with previous renal artery or renal vein thrombosis) | **PERIOD 07/1985-06/1991** (n=475)  no antiplatelet | Renal graft vein thrombosis (RVT) | First 30 days posttransplant | - 2.7% in aspirin group - N/A in no antiplatelet group | RVT incidence (cohort 1 vs 2): 5.6% vs 1.2%, p< 0.01 | high |
| Hernández et al, 2006, Nephrol Dial Transplant | | Single centre retrospective cohort study (years 1996-2004) | Adult deceased KTRs | n=870 | Pretransplant antiplatelet drug use (n=307) | No pretransplant antiplatelet drug use (n=563) | Bleeding complication requiring reintervention, aspiration or transfusion | First 3 months posttransplant | - 40% vs 34 %, p=0.005 - Multivariable analysis: OR=1.8 (95%CI, 1.03-3.3) | N/A | high |
| Rodriguez-Pastore et al, 2012, Am J Transplant  (*abstract only*) | | Single centre randomized controlled study ***(Comparison 1***) (years 2007-2009) | KTRs | n=30 | Patients on preoperative ASA randomized to platelet transfusions (one pool before, during and after surgery) | Patients on ASA randomized to no platelet transfusions | - Change in Hgb 4 h post-surgery - Perirenal bleeding on ultrasound 24h posttransplant | First 24h posttransplant | - Perirenal bleeding on ultrasound: 58% vs 42% (no p-value provided) - Similar Hb levels pre-transplant and 4h post-transplant | N/A | high |
|  |  | Single centre observational cohort study (***Comparison 2***) (years 2007-2009) |  | n=100 | Patients on preoperative ASA (either receiving platelet transfusions or not) (n=30) | Patients not on ASA (n=70) |  |  | - Perirenal bleeding on ultrasound: 17,5% vs 58% and 42% in ASA treated patients receiving platelets or no platelets, respectively (no p-value provided) - Similar Hb levels pre-transplant and 4h post-transplant |  |  |
| Benahmed et al, 2014, Nephrol Dial Transplant | | Single centre retrospective cohort study (years 2000-2010) | Adult KTRs | n=58 | Clopidogrel (n=8) or ticlopidine (n=11), some with low-dose aspirin (n=8) [DAPT] varying dose 80-160mg | Controls randomly selected within cohort not on clopidogrel or ticlopidine (n=39) | - Peri and post-operative bleeding - DVT or PE - Cardiovascular complications | First 30 days posttransplant | - One case intra-operative bleeding (implant biopsy) needing platelet transfusion - No reoperation for bleeding in either group - No difference between transfusion rates | - No cardiovascular events or DVT/PE in either group. | high |
| Bailey et al, 2015, Austin J Nephrol Hypertens | | Single centre retrospective cohort study (years 2011-2014) | Adult KTRs | n=367 | - Pretransplant ASA 100 mg (n=150) - Pretransplant ASA 100 mg + clopidogrel 75 mg (n=23) | No antiplatelet (n=209) | - Blood transfusions within 5 days - Reoperation for bleeding | First 5 days posttransplant | - Blood transfusions: 42% vs 52% vs 28% (p<0.01) - No difference in reoperation for bleeding | N/A | high |
| Benkö et al, 2018, Int J Org Transplant Med | | Single-centre retrospective cohort study (years 2007-2012) | Adult KTRs receiving antiplatelet (clopidogrel, aspirin or both) | n=161 | Antiplatelet monotherapy (clopidogrel or aspirin) | DAPT (clopidogrel + aspirin) | - Post-operative bleeding (Hb decrease>3.5g/dL compared to pre-operative baseline within the first 4 post-operative days) | First 4 days posttransplant | - Postoperative bleeding (recipients with monotherapy vs DAPT): 31.5% vs 60%, p=0.027 - Logistic regression analysis for post-operative bleeding: living related kidney transplantation [OR=0.16 (95%CI, 0.04-0.58), p=0.002], CAD [OR=2.29 (95% CI, 1.57-2.77), p=0.043], DAPT [OR=7.04 (1.9-44.5), p=0.002] | N/A | high |
| Kapeles et al, 2018, Am J Transplant  (*abstract only*) | | single centre retrospective cohort study (years 2000-2008) | Adult KTRs | n=1752 | Pre-transplant clopidogrel (n=46) | No clopidogrel (n=1521) | - Blood transfusions | N/A | - Perioperative blood transfusion: 39.1% vs 11.3%, p<0.001 | N/A | high |
| Lee et al, 2020, Transplant Rev | | systematic review and meta-analysis (years N/A) | Adult KTR | n=911 | pretransplant DAPT (n=130) | pretransplant single antiplatelet (SAPT) (n=781) | - Post-operative haemorrhagic events | N/A | - significantly higher risk of haemorrhagic events in the DAPT compared to SAPT group (RR 1.58, 95% CI 1.19-2.09) | N/A | N/A |
| Bansal et al, 2022, Transplantation  (*abstract only*) | | single centre retrospective cohort study (years 2014-2021) | Adult KTRs with CAD taking DAPT: | n=106 | Group A: pretransplant continued DAPT (clopidogrel 75 mg and ASA 150 mg) (n=73) | Group B: DAPT discontinued 5 days prior to procedure) (n=33) | - Pre- and postoperative haemoglobin - Intraoperative blood loss - Drain output - Need for transfusion - Bleeding complications (need for any intervention) - Any cardiovascular events | first 30 days posttransplant | - Blood loss (group A: 290 ml vs group B 280 ml, p=0.21) - Blood transfusion rates (group A: 24/73 vs. group B: 8/33, p=0.29) - No patients required any intervention for bleeding. | No cardiovascular events in either group | high |
| **Anticoagulants** | | | | | | | | | | | |
| Wong et al, 2006, Nephrology  (*abstract only*) | Retrospective case-control study (years 1994-2005) | | Adult KTRs | n=996 | preoperative warfarin use (n=26) | Authors mention having performed a case control analysis with matching for age, gender and diabetic status. Number of controls in the comparison not provided. | - Transfusions - Wound complications | N/A | - Wound complications were not different - Patients post-transplant anti-coagulated (n = 18) had an increased risk of reoperation (22% vs. 0%, p = 0.023) - No patients or grafts were lost from haemorrhage | N/A | high |
| Kikić et al, 2009, Transplantation | Single centre randomized controlled study (years 2003-2008) | | Adult deceased-donor KTRs | n=110 | pretransplant heparin haemodialysis (n=66) | pretransplant citrate haemodialysis (n=44) | Bleeding complications | First week post-transplantation | - Bleeding rates similar between groups (11% vs 16%, p not provided) | N/A | Some concerns |
| Connaughton et al, 2010, J Nephrol | Single centre retrospective cohort study (years 2000-2008) | | Adult deceased-donor KTRs | n=93 | Pretransplant warfarin with target INR 2-3 (n=31) | Sex-matched controls (n=62) | - Haemoglobin levels in first week posttransplant - Need for blood transfusions - Need for FFP administration | N/A | - Similar haemoglobin levels immediately before surgery [12.48 (range 6.8-16.0 g/dL) vs 12.58 (7.2-16.2 g/dL), p=0.827], the day after surgery [10.26 (7.0-13.4 g/dL) vs 10.9 (8.2-14.2 g/dL), p=0.198] and and at 1 week postoperatively [10.39 (8.0-13.3 g/dL) vs 10.38 (7.6-13.3 g/dL), p=0.515]. - Patient requiring blood transfusions: 45% vs 29% (p=0,22), number of blood transfusions (median): 4,5 vs 2 units (p=0,057) - Patients requiring FFP post-operatively (to correct for a bleeding diathesis especially if the patient suffered a haemorrhagic complication postoperatively): 84% vs 11% (p<0,0001), number of FFP units (median): 8 vs 4 units. | N/A | high |
| Lange et al, 2021, Clin Transplant | Single centre case series (years N/A) | | Adult deceased-donor KTRs on apixaban pre-transplant | n=5 | - apixaban 5 mg bid (n=2) - apixaban 5 mg (n=1) - apixaban 2,5 mg bid (n=1) - apixaban with unknown dose (n=1) | None | - Receipt of blood products or haemostatic agents - Changes in relevant laboratory measurements | Early post-operative period | - No patient received haemostatic or reversal agent prior to transplantation. - One patient received a single unit of platelets intraoperatively (thrombocytopenia was noted for this patient). - Blood loss: 100-1600 ml. - One patient experienced major vascular injury, needed 3 units of PRBC during surgery. - Two patients received PRBC post-operatively for anaemia. | N/A | high |
| Littlejohn et al, 2023, Am J Transplant  (*abstract only*) | Single centre case series (years 2016-2022) | | Adult deceased-donor KTRs on apixaban pre-transplant | n=15 | - apixaban 5 mg (n=7) - apixaban 2.5 mg (n= 5) - apixaban and aspirin (n=3)   *Apixaban was held 10 h prior to surgery in 1 patient, between 12-25 h prior to surgery in 12 patients, 2 did not have clear documentation. One patient received PCC pre-op. and 2 units PRBC intra-op.* | None | - Reversal agents use - Transfusion requirements - Operative blood loss - Re-operation for bleeding - Haemoglobin decrease | Early post-operative period | - Mean blood loss 200 ± 155.5 ml - 2 patients required hematoma evacuation an - 1 patient required transfusion post-operatively - Maximal haemoglobin decrease: 25.4 ± 9.9 % | N/A | high |
| **Antiplatelets + Anticoagulants** | | | | | | | | | | | |
| Eng et al, 2011, Clin Trans | | Single centre, retrospective cohort study (years 2005-2009) | Adult KTRs | n=327 | - Pre-operative anticoagulation (warfarin) or antiplatelet (ASA or clopidogrel) (n=105) - Post-operative anticoagulant (heparins or warfarin) or antiplatelet (ASA) (n=28) | No anticoagulant or antiplatelet (n=213) | - Reoperation for bleeding - Transfusion - Decrease in haemoglobin | N/A | - Pre-operative therapy and no post-operative compared to none: no increase in reoperation, transfusion, Hb drop. - Post-op therapy (with or without pre-operative) compared to none: heparin infusions associated with increased bleeding, other therapy not statistically significant | N/A | High |
| Marzouk et al, 2013, Transplant Res | | Single centre retrospective cohort study (chart review) (years 2006-2012) | Adult deceased donor KTRs | n=306 | Patients on warfarin (n=14) or antiplatelet therapy (n=136) pretransplant | No anticoagulants (n=156) | - Transfusion | First hospitalization or 30 days posttransplant (whichever came first) | - Transfusion rates higher in patients on warfarin (8/14, 57%, 5.1 units/transplant) and antiplatelets (46/136, 33.8%, 1.14 units/transplant) vs. those not on anticoagulants (26/156, 16.7%, 0.47 units/transplant) - patients with large transfusion requirements ((≥5 units): 9 on antiplatelets, 4 on warfarin and 3 not on anticoagulant - multivariable logistic regression analysis: warfarin (OR=8.2, 95% CI 2.5–27, P=0.001) and antiplatelet use (OR=2.9, 95% CI 1.6–5.3, P=0.001) independent predictors of blood transfusion | N/A | high |
| Koch et al, 2015, Clin Transplant | | Single centre retrospective cohort study (years 2007-2012) | Adult KTRs | n=429 | Preoperative anticoagulant (heparin, phenprocoumon) or antiplatelet (ASA clopidogrel) (n=119) | No anticoagulant or antiplatelet (n=310) | - Surgical complication - Severe complications (Clavien ≥ III) - Bleeding - Wound healing disorders - Lymphoceles | N/A | - OR aspirin vs controls 0.9 [0.5; 1.8] p=0.8 - OR other anticoagulation vs control 1.8 [0.8; 4.3] p=0.17 | N/A | high |
| Musetti et al, 2015, J Nephrol | | Single centre retrospective cohort study (years 1998-2013) | Adult KTR | n=911 | - Pretransplant single or dual antiaggregant therapy (AAT, n=336), of which dual AAT n=12 - Pretransplant vitamin k antagonist (VKA, n=37) | No AAT or VKA (n=538) | - Early haemorrhagic events - Cardiovascular events for temporary drug withdrawal | 90 days post-transplant | - Haemorrhagic events: AAT 2.7% vs VKA 10.8% vs controls 1.5% (VKA vs controls p<0.05, AAT vs VKA p<0.05) | - Post-transplant cardiovascular events 4.5% in VKA group vs 13.5% in AAT group vs 2.2% in controls (VKA vs controls p<0.05, AAT vs VKA p<0.05) | high |
| Weng et al, 2016, Am J Transplant  (*abstract only*) | | Single centre, retrospective cohort study (years 2008-2014) | Adult KTRs with CAD and DAPT | n=149 | - aspirin and clopidogrel (DAPT) (n=66) | Single antiplatelet therapy (aspirin or clopidogrel) or warfarin (n=83) | - Transfusions - Haematoma requiring drainage by 90 days - Readmission for cardiovascular reasons | N/A | - Higher transfusions frequency during transplant hospitalization in DAPT group (30.3% vs 15.7%, p=0.03) - Frequency of haematomas requiring drainage similar between groups (DAPT vs non-DAPT, 4.6% vs 8.4%, p= 0.35) | - Readmission for cardiovascular reasons similar between groups (DAPT vs non-DAPT, 9.1% vs 7.2%, p=0.68) | high |
| Hachem et al, 2017, Transplant Int | | Single-centre case–control study (years 2000-2012) | adult KTRs  **Matching ratio** 1:5 | n=354 | - KTRs with postoperative surgical-site hemorrhage (drop in hemoglobin ≥20 g/l occurred over a 24-hour period within 3 days after transplantation + ultrasound within 1 day prior to 2 days following the hemoglobin drop with a hematoma or large collection or with a volume ≥33.2 cm3 / diameter of 4 cm in all dimensions) (n=59) | KTRs without postoperative surgical-site hemorrhage matched to cases for year of transplant (n= 295), 1:5 match | **Risk factors for Postoperative surgical-site hemorrhage** | First 3 days posttransplant | - time-frame of bleeding cases: 47.45% within 24 hours post-transplant, 42.37% between 24-48 hours post-transplant, 0% after 48 hours days post-transplant - chronic anticoagulant or antiplatelet therapy non-significantly associated with risk of postoperative surgical-site haemorrhage [OR 1.75 (95%CI: 0.52, 5.88), P = 0.36 and OR 1.26 (95% CI: 0.70, 2.27), P = 0.43, respectively] | N/A | high |
| van den Berg et al, 2019, Transplant Int | | Two-center retrospective cohort study (years 2011-2016) | Adult KTRs | n=2000 | 1) intraoperative unfractionated heparin (**UFH group**)  2) continued use of VKA (defined as preoperative not corrected INR > 1.5 and last administration at day of transplantation) + VKA bridged with heparin **(VKA group**)  3) continued use of single or dual antiplatelet therapy (**APT group**)4) combined use of APT and UFH (**APT+UFH group**) | none (control group, CG) | - post-operative bleeding ≤7 days post-transplantation (clinical symptoms + sudden drop of haemoglobin, noticed by treating physician, or compression on the graft as confirmed on imaging + require for further intervention, by blood transfusion, re-exploration or both) - post-operative thromboembolic complications (DVT extending to the popliteal vein or more proximally, PE, thrombosis of the femoral artery, renal artery or renal vein thrombosis confirmed ≤7 days post-transplantation) | First 7 days posttransplant | - post-operative bleeding incidence significantly different between groups [46/88 (52%) CG group vs five (6%) UFH group vs 15/88 (17%) VKA group vs 20/88 (23%) APT group 2/88 (2%) APT + UFH group; p=0.006] - median time for development of bleeding, 2nd postoperative day (1–4) (n = 45, 51%), with a peak on the first day (n = 36, 41%) - risk factors for post-operative bleeding in univariable analysis: continued use of VKA [OR 10.13 (95% CI: 5.33–19.52), P < 0.001] - risk factors for post-operative bleeding in multivariable analysis: continued use of VKA [OR 6.60 (95% CI: 2.95–14.77), P < 0.001] / antiplatelet therapy not associated with increased risk (OR 1.23 (95% CI:0.74–2.04), P = 0.44) | - Post-operative thromboembolic complications incidence not different between groups [14/21(67%) CG group vs 2/21(11%) UFH group vs 1/21 (5%) VKA group vs 3/21(14%) APT group vs 1/21 (5%) APT+UFH group; p=0.69] - median time for development of thromboembolic events : 1 day (1–3) (n = 13, 62%) | high |
| Buggs et al, 2020, Am Surg | | Single center retrospective cohort study (years 2012-2019) | Adult KTRs with post-transplant haemorrhage (defined as hematoma on ultrasound or CT) | n=135 | **Group 1**: haemorrhage with operative management (n=66) | **Group 2**: haemorrhage with non-operative management (n=69) | - Admissions due to cardiac and pulmonary complications | N/A | - Pre-operative anticoagulant use did not differ between two groups (P =0.39) | - Admissions due to cardiac (Group 1 vs Group 2, 21.2% vs 14.5%, p=0.37) and pulmonary complications (6.1% vs 7.2%, p=1.00) similar between groups | high |
| Diamond et al, 2020, Am J Transplant  *(abstract only)* | | Retrospective study (years 2008-2013) | Adult KTRs | n=1400 | - Group 1: anticoagulant (n=562) - Group 2: antiplatelet (n=138) | - Group 3: no anticoagulant (n=562) - Group 4: no antiplatelet (n=138) | - major bleeding [intracranial (ICH), gastrointestinal (GIH), subarachnoid (SAH), intracerebral (INCH), subdural (SDH), and extradural (EDH) haemorrhage] - thromboembolic complications [incidence of renal vein thrombosis (RVT), deep vein thrombosis (DVT), pulmonary embolism (PE)] | N/A | - GIH, group 1 vs group 3, 0.8% vs 0.4%, p=0.255 and group 2 vs 4, 0% vs 0.7%, p=0.99 - SDH, group 1 vs group 3, 0.1% vs 0%, p=0.317 and group 2 vs group 4, 0% vs 0%, p=N/A - ICH, SAH, INCH, EDH, no events | - RVT, group 1 vs group 3, 0.3% vs 0.5%, p=0.654 and group 2 vs group 4, 0% vs 0.7%, p=0.99 - DVT, group 1 vs group 3, 1.6% vs 0.5%, p=0.144 and group 2 vs group 4, 0% vs 1.4%, p=0.498 - PE, group 1 vs group 3, 0.3% vs 0%, p=0.5 and group 2 vs group 4, 0% vs 0%, p=N/A | high |
| Alonso-Escalante et al, 2021, Ann Transplant | | Single centre retrospective cohort study (years 2017-2019) | Adult live-donor and deceased-donor KTRs | n=252 | pre-transplant warfarin only (n=32) or antiplatelet (ASA, clopidogrel) (n=32) (doses not reported) | no anticoagulant or antiplatelet (n=197) | - Graft exploration for bleeding at 3-months and 6 months after transplantation - Perioperative transfusion requirements | 6 months posttransplant | - 3-month graft exploration rates similar between groups (warfarin 0%, ASA/CLOP 3%, and control 3%, p=0.69) - 6-month graft exploration rates similar between groups (warfarin 4%, ASA/CLOP 0%, control 1%, p=0.12). - Perioperative blood transfusion rates similar (warfarin 4%, ASA/CLOP 13%, control 4%, p=0.13) | N/A | high |
| Hau et al, 2022, J Clin Med | | Single centre retrospective cohort study (years 2011-2018) | Adult KTRs | n=204 | **Group B** (n=96) on antithrombotic medications at the day of transplantation and post-operative continued use of sub-therapeutically antithrombotic medications   - pre-transplant therapeutic dose heparin (n=22) - pre-transplant vitamin K antagonists (VKA) or DOACs (n=8) - pretransplant antiplatelet and prophylactic dose heparin (n=53) - pre-transplant antiplatelet and therapeutic dose heparin (n=13) | **Group A** (n=108) without pre-transplant anticoagulant or antiplatelet therapies and receiving postoperative prophylactic heparin | - Post-operative bleeding [defined as decrease in haemoglobin levels more than 2 g/dl over 24 h with the need for blood transfusion and or intervention (surgery or drainage) and/or ultrasonographic identification of hematoma or large fluid collection] - Thromboembolic events (Not specified | N/A | - A total of 61 pts developed post-operative bleeding complication. - Percentage of the patients with prior anticoagulant use significantly higher in bleeding to no-bleeding group (43% vs.33%, p=<0.05) - No significant difference in use of prior antiplatelets between bleeding and no-bleeding groups (43% vs. 33%, p=0.183) - Multivariate analysis: pre-operative VKA therapy increases the risk of bleeding (OR=5.89, 95CI% 1.10-31.28; p=<0.05), use of prior antiplatelets does not increase risk of bleeding (OR=1.63, 95CI% 0.85-3.15; p=0.144). | - Thromboembolic complications not significantly different between groups (group A: 3% vs. group B: 8%; p=0.08) | high |

**Supplementary Table 11:** Summary Evidence Table for Question 2 – Should kidney transplant recipients receive prophylaxis for venous thrombosis after surgery?

| **Author, year, Journal**  *(displayed in chronological order)* | **Study design/Time frame** | **Patient characteristics** | **No. of patients** | **Antithrombotic drug and dosage** | **Comparator (if applicable)** | **Outcomes** | **Follow-up for the outcomes of interest** | **Bleeding events** | **Thrombotic events** | **Risk of bias** |
| --- | --- | --- | --- | --- | --- | --- | --- | --- | --- | --- |
| **Anticoagulants** | | | | | | | | | | |
| Ubhi et al, 1989, Transplantation | Single-centre, prospective randomized study  (years 2005 – 2016) | Adult cadaveric KTRs | n=69 | subcutaneous calcium heparin 5000 units bid for 7 days or until the patient was fully mobile about the ward (n=32) | no prophylaxis (n=37) | - Venous thromboembolism - Post-operative wound drainage - Wound/peri-transplant collection | 30 days post transplantation | - Postoperative wound drainage: heparin group, 248 (34-612) vs no-heparin group, 226 (76-1404) ml, p>0.05 - Wound or peri-transplant collections incidence: heparin group, 6 vs no-heparin group, 3, p>0.05 | - Incidence of thromboembolic events: 6 in no-heparin vs 0 in heparin group (0.05<p<0.1 with Yates correction) | high |
| Alkhunaizi et al, 1998, Transplantation | Single-centre prospective cohort study (years 1996-1997) | Adult living-donor and cadaveric KTRs | n=120 | - **Group 1 (**with normal coagulation panel): a single dose of 2500 U of dalteparin subcutaneously immediately post-surgery and then daily during hospitalization - **Group 2** (with abnormal coagulation screen or with past history of hypercoagulable state): a single daily dose of 5000 U of dalteparin immediately post-surgery and for 1 month after transplantation and then converted to oral warfarin | N/A | - Significant bleeding (defined as the necessity for surgical intervention or transfusion of more than 2 U of blood) - Allograft thrombotic events (arterial or venous) - DVT | 1 year post-transplant | - No significant bleeding events | - No allograft arterial or venous thrombosis - 1 event of DVT (right subclavian vein line) | high |
| Lundin et al, 2002, Ups J Med Sci | Single-centre retrospective cohort study (years N/A) | adult KTRs | n=120 | - **Group A** (n=56) prophylaxis with: a) enoxaparin 20mg (n=50) or 40mg (n=3) od b) dalteparin 2500 U od (n=1) or c) heparin 5000 U od (n=2) | - **Group B** (n=64): no prophylaxis | - bleeding complications leading to reoperation | N/A | - Incidence of bleeding complications needing reoperation: similar between groups (4 vs 3, p=ns) | N/A | high |
| Osman et al, 2007, Urology | Single-centre prospective randomized controlled study (years 2003-2005) | adult KTRs | n=75 | - **Group 2**: prophylactic dose of LMWH for 1 week postoperatively (subcutaneous tinzaparin sodium od 3500 anti-Xa IU in 0.35 mL) (n=25) - **Group 3**: subcutaneous conventional unfractionated heparin for 1 week postoperatively at a dose of 5000 IU bid (n=25) | - **Group 1**: no heparin (n=25) | - Thromboembolic events - Bleeding events | Early post-transplant period | - 1 patient in group 2 developed massive haemorrhage necessitating re-exploration. - Incidence of significant perirenal hematoma / massive haemorrhage similar between groups (Group 1 vs Group 2 vs Group 3, 0/25 vs 1/25 vs 0/25, p=0.37) - Incidence of blood transfusions (8/25 vs 11/25 vs 12/25, p=0.56) and mean number of transfused units (0.5±0.8 vs 0.6±0.8 vs 0.6±0.7, p=0.69) similar between groups - Rate of haemoglobin decrease in non-transfused patients gradually increased from Group 1 to Group 3 (0.7±0.9 vs 1.1±0.5 1.6±0.8, p=0.01) | - No patient with vascular thrombosis of the graft, DVT, or PE - Incidence of closure of AV fistula similar between groups (1/25 vs 1/25 vs 2/25, p=0.79) | low |
| Ng et al, 2016, Can J Hosp Pharm | Single-centre retrospective cohort study (years 2008-2013) | Adult KTRs | n=547 | - **Group 1**: prophylactic heparin in the early postoperative period (n=266) - **Group 2**: therapeutic heparin in the early postoperative period (n=13) | - **Group 3**: no heparin in the early postoperative period (n=268) | - Major bleeding (hematoma requiring surgical evacuation, retroperitoneal bleeding, intracranial bleeding, documented bleeding requiring 2 or more units of packed red blood cells, or documented bleeding with a drop in haemoglobin level of 20 g/L or more) - Thromboembolic events (arterial, venous) | 7 days post-transplant | - Major bleeding incidence: higher in therapeutic heparin group and similar between prophylactic and no heparin groups [therapeutic heparin vs prophylactic heparin vs control, 46% (6/13) vs 3% (8/266) vs 3.4% (9/268); p> 0.99 for comparison between prophylactic and no heparin groups] | - Thrombotic events incidence: similar between groups [therapeutic heparin vs prophylactic heparin vs control, 0% (0/13) vs 0.4% (1/266) vs 3.4% (9/268); ); p> 0.99 for comparison between prophylactic and no heparin groups] | high |
| van den Berg et al, 2019, Transpl Int | Two-centre, retrospective cohort study (years 2011-2016) | adult KTRs | n=2000 | Unfractionated heparin 12,000 IU/d i.v. for 5 days or LMWH 2850 IU s.c. plus one of the following:   - Intra-operative heparin 5000 U (pre-emptive Tx in one centre) (n=195) - VKAs with INR>1.5 at day of surgery (n=53) - Antiplatelet therapy (APT) continued until surgery (n=390) - Intra-operative heparin plus APT (n=29) | Unfractionated heparin 12,000 IU/d i.v. for 5 days or LMWH 2850 IU s.c. (n=1333) | - Bleeding events (≤7 days post‐transplantation with drop in haemoglobin or compression on the graft) - Thromboembolic events (DVT, extending to the popliteal vein or more proximally, all cases of PE and thrombosis of the femoral artery, confirmed by radiological imaging ≤7 days after surgery OR renal artery or renal vein thrombosis ≤7 days post‐transplantation) | N/A | - Incidence of bleeding events different between groups and highest in VKA group: 46 (3.45%) in controls, 20 (5.13%) in patents with APT, 5 (2.56%) in patients with UFH, 15 (28.3%) in patients with VKA, 2 (6.9%) in patients with APT+UFH - Median time for development of bleeding: 2nd postoperative day (1–4) (*n* = 45, 51%), with a peak on the first day (*n* = 36, 41%). - Multivariable analysis for risk factors for bleeding: post-operative heparin (OR=1.69, 95%CI, 1.00–2.85, p=0.049) | - Incidence of thromboembolic events similar between groups [14 (1,05%) in controls, 3 (0.77%) in patents with APT, 2 (1.03%) in patients with UFH, 1 in patients with VKA (1.89%), 1 (3.49%) in patients with APT+UFH; p=0.69] - Median time for the development of thromboembolic event: 1st postoperative day (1–3) (*n* = 13, 62%) | high |
| Qu et al, 2023, Can J Surg | Single-centre, retrospective, study (years 2005-2016) | Adult KTRs | n=1667 | at least 1 dose of subcutaneous unfractionated heparin 5000 units administered between 24 hours before transplant and 48 hours after transplant (n=849) | no prophylaxis (n=818) | - DVT, excluding graft thromboses | 3 months posttransplant | N/A | - Use of prophylaxis not significantly associated to DVT both in univariable (HR=0.98, 95%CI, 0.62, 1.56; p=0.94) and multivariable analyses (HR=0.76 95%CI, 0.40, 1.45; p=0.41) | high |
| **Antiplatelets** | | | | | | | | | | |
| Robertson et al, 2000, Nephrol Dial Transplant | Single-centre retrospective cohort study (years 1985-1997) | Adult KTRs (cadaveric and living-related) | n=955 | **PERIOD 07/1991-06/1997 (n=480)**   - low-dose aspirin (75mg od) for 1-month post-transplant (if high risk patients, i.e., with diabetes, history of DVT or PAD, for long-term) - high-dose aspirin (150mg od) for long-term (in patients with previous renal artery or renal vein thrombosis) | **PERIOD 07/1985-06/1991 (n=475)** no antiplatelet | - renal vein thrombosis | two 6-year periods (07/1985-06/1991, 07/1991-06/1997) | **(data only since 1991)**   - post-biopsy bleeding events (n=8, 1.6% of biopsies, compared to previous reports of 0.3-12.6%) - early post-transplant bleeding events (n=3, 1%) - re-exploration for bleeding (n=3) - graft loss directly attributable to aspirin (n=0) | - RVT occurrence statistically higher during pre-aspirin vs aspirin period [5.6% (27/475) vs 1.6% (6/480), p<0.01] | high |
| Murphy et al, 2001, Br J Surg | Single-centre consecutive case series (years 1990-1995) | adult KTRs (cadaveric) | n=226 | **PERIOD 1993-1995** aspirin 150mg od for 3 months post-transplant (SC 5000 U heparin pre- and 5000 U bid 5 days post-transplant) (n=105) | **PERIOD 1990-1992** no antiplatelet (SC 5000 U heparin pre- and 5000 U bid 5 days post-transplant) (n=121) | - graft thrombosis - aspirin-related complications | 2 year posttransplant | - incidence of macroscopic haematuria following graft biopsy not different between groups (9% vs 7% for aspirin vs control group, p=ns) | - Rate of primary allograft thrombosis lower with aspirin [0/105 (0%) vs 6/121 (6%) for aspirin vs control group, p=0.03] | high |
| Pegler, 2023, Ann Transplant | Single-centre, retrospective (years 2014 – 2021) | adult KTRs | n=1208 | aspirin for >6 weeks (n=637) | aspirin for 5 days (n=571) | - VTE within 6 weeks, (defined as DVT or PE confirmed on imaging via ultrasound, CT, or ventilation-perfusion scan, excluding localized events associated with intravenous lines - Renal vein/artery thrombosis (causing graft loss - Blood transfusion from date of transplant | 6 weeks posttransplant | - blood transfusion: 53 (>6 weeks) and 50 (5 days), p=0.8 | - VTE: 8 vs 8 - Renal vein thrombosis: 1 vs 0 (p=ns) - Renal artery thrombosis: 2 vs 0 (p=ns) | high |

**Supplementary Table 12:** Summary Evidence Table for Question 3 – Can we use direct-acting oral anticoagulants (DOACs) in kidney transplant recipients in terms of safety (dosing, drug- drug interactions, adverse events)?

| **Author, year, Journal**  *(displayed in chronological order)* | **Study design/ Time frame** | **Patient characteristics** | **No. of patients** | **Antithrombotic drug and dosage** | **Comparator (if applicable)** | **Outcomes** | **Follow-up for the outcomes of interest** | **Bleeding events** | **Thrombotic events** | **Drug-drug interactions** | **Risk of bias** |
| --- | --- | --- | --- | --- | --- | --- | --- | --- | --- | --- | --- |
| Vanhove et al, 2017, Ther Drug Monitor | Single-centre retrospective study (years 2012-2016) | Adult transplant recipients treated with CNI and DOAC  Transplant type:   - 18 kidney - 13 lung - 2 kidney + lung - 5 heart - 1 allogenic stem cell | n = 39 | - Rivaroxaban (n=29) - Apixaban (N=10)   (drug dose not reported) | N/A | - Dose-corrected CNI trough concentrations (C_0_/D) before and during DOAC therapy | 200 days | - N/A | - N/A | - Group average C0/D not significantly different before and during DOAC therapy for any CNI-DOAC combination (P = 0.089-0.761), although C0/D changed >20% in 19/39 patients (13 increases, 6 decreases) - subgroup analysis per DOAC: limited but significant effect of rivaroxaban on tacrolimus C0/D (9.2% increase, P = 0.042). | low |
| Bukhari, 2019, World J Transplant | Single-centre retrospective cohort study (years 2015-2016) | Adult KTRs | n=42 | - apixaban (n=29, 12 on 5mg bid and 17 on 2,5 mg bid) - rivaroxaban (n=10, 5 on 20 mg od and 5 on low dose) - dabigatran (n=3, 2 on 150 bid and 1 on low dose) | N/A | - thromboembolic events - bleeding events - interactions with CNIs | 1 year | - Bleeding events: 3 (7.1%) - 1 patient treated with rivaroxaban 15 mg daily developed intraocular bleeding - 2 patients treated with apixaban 2.5 mg bid developed rectal bleeding | - No thromboembolic events | - No significant changes in serum tacrolimus level 3 days after the initiation of DOACs (pre- vs. post-DOACs, 7.2516 vs. 7.8867 ng/mL, P = 0.55) | high |
| Do et al, 2019, Am J Transplant  *(abstract only)* | Single-centre retrospective study (chart review) (years 2015-2017) | Adult KTRs | n = 52 | - apixaban, (drug dose not reported) | N/A | - bleeding events - thromboembolic events   (with sub-analysis of patients undergoing renal transplant biopsy) | 12 months | - 2 (3.8%) major and 4 minor bleeding events - 18 patients underwent biopsies and apixaban was stopped 2 days prior biopsy with 1 bleeding event post-biopsy | - 2 (3.8%) thrombotic events (none in patients with biopsy) | N/A | high |
| Herber et al, 2019,  J Hepatol  *(abstract only)* | Single-centre prospective study  (years 2014-2018) | adult transplant recipients  Transplant type:   - Liver (n=21) - Kidney (n=16) - Combined (n=5)   Indications for DOAC: non-valvular AF,  DVT, pulmonary embolism, portal vein thrombosis | n=42 | - Rivaroxaban (n=23) - Apixaban (n=19) | N/A | - bleeding events   a) minor bleeding (spontaneous hematoma, prolonged bleeding after trivial trauma, gingival bleeding)  b) major bleeding (requiring hospitalization or transfusion)   - thromboembolic events |  | - No major bleeding events - Minor bleedings 18/30 (60%): 12/18 treated with rivaroxaban and 6/18 with apixaban - Epistaxis 3/30 (10%) | - 1 myocardial infarction in a patient with underlying malignant disease | - Dose of immunosuppressive agents after DOAC initiation adjusted in 15 (35.7%) cases. - In patients without immunosuppressive dose change (n=27), mean plasma drug levels increased in 19 (70.3%) patients and decreased in 8 (29.7%) - In all patients, mean plasma drug levels within target range. - In patients with available data (n=22), trough levels of DOAC with therapeutic range | high |
| Basic-Jukic et al, 2020, Pril (Makedon Akad Nauk Umet Odd Med Nauki) | Single-centre retrospective cohort study (years 2007-2019) | Adult KTRs   indications for DOACs: AF 14/23, DVT 5/23, PE 2/23, AF+DVT 1/23, DVT+PE 1/23 | n=23 | - Rivaroxaban (n=14) - Apixaban (n=8) - Dabigatran (n=1) | NA | - bleeding events - thromboembolic events - cyclosporine and tacrolimus trough levels before, at one month and 12 months after the introduction of NOACs | 24 months | - 2/23 patients (1/23 treated with rivaroxaban and 1/23 with dabigatran), with the latter requiring treatment cessation and switch to warfarin | - no thromboembolic events | - stable cyclosporine levels under rivaroxaban [before vs 1 vs 12 months post- initiation, 95.5 (75.8–105.8) vs 100 (75-111.8) vs 101.5 (61.5-104.30, p=0.74] and a trend for lower cyclosporine levels under apixaban [before vs 1 vs 12 months post- initiation, 94.5 (57.8-99.5) vs 84 (54.8-86.8) vs 81 (54-83), p=0.14] - significant decrease in tacrolimus levels under rivaroxaban [before vs 1 vs 12 months post- initiation, 7.5 (4.8-6.5) vs 7.6 (5.3-6.0) vs 5.3 (3.8-4.1), p=0.22] and a trend for lower tacrolimus levels under apixaban [before vs 1 vs 12 months post- initiation, 6.7 (5.3-8.2) vs 5.2 (3.9-7) vs 4.4 (4.03-5.8), p=0.02] | high |
| Bixby et al, 2020, Transplant Int | Single-centre retrospective cohort study (years 2011-2018) | Adult KTRs | n = 197 | - apixaban (n=60) - dabigatran (n=6) - rivaroxaban (n=33) | warfarin group (n=98) | - Major bleeding (decrease of Hb more than 2 g/dl, transfusion, bleeding in organs or critical area - Clinically relevant non-major bleeding (CRNMB) (sign or symptom of hemorrhage that does not fit the criteria for major bleeding but requires medical intervention, leads to hospitalization or increased level of care, or prompts a face-to-face evaluation) - Composite bleeding (major bleeding and CRNMB), - New-onset or recurrent VTE - New-onset stroke - Drug-drug interaction | Median of 12,3 (IQR 5.2–27.5) months | - No difference in major bleeding events between patients on DOACs vs. warfarin in the time-to-event analysis (p = 0.15) - Rates of major bleeding: 7.2% per year with DOACs vs. 11.4% per year with warfarin - No difference in classification of major bleeding (p=0.13), but numerically more warfarin patients with major bleeding event had bleeding at a critical site or organ (38% vs. 11%) - Lower incidence of major bleeding (6.7% vs. 21.4%, P = 0.014) and a trend toward lower composite bleeding (21.7% vs. 34.7%, p = 0.08) in patients with apixaban vs warfarin - Lower incidence of major bleeding (6.7% vs. 19.0%, P = 0.027) but no difference in composite bleeding (21.7% vs. 32.8%, P = 0.11) in patients with apixaban vs all other agents. - Multivariable Cox regression: DOAC use not associated with increased risk of bleeding vs. warfarin (HR=0.73, 95%CI, 0.27–1.95; p=0.529) - Sensitivity analysis including patients without prior bleeding events, DOAC use not associated with major bleeding vs. warfarin (HR=0.83, 95%CI, 0.26–2.67; P=0.759) | - New VTE not different between groups: 3.1% in warfarin group vs. 2.0% in DOACs group (p = 0.50) - New stroke not different between groups: 5.1% in warfarin group vs. 1.0% in DOACs group (p = 0.13) - No differences between different DOAC agents regarding stroke and VTE events | - Present drug interactions not different between groups (p=0.29) | Some concerns |
| Camporese et al, 2020, Vascul Pharmacol | Two-centre prospective study (years 2016-2018) | Adult KTRs | n = 8 | rivaroxaban | N/A | - Drug-drug interactions - Major bleeding events - Thromboembolic events | 2 weeks | - no major bleeding events | - no thromboembolic events | - Variability (percentage of coefficient of variation) before and after rivaroxaban not different (30% vs 30% for tacrolimus, 27% vs 29% for everolimus) | high |
| Leon et al, 2020, Transplantation | Single-centre retrospective case-cohort study (years 2013-2018) | Adult KTRs indications for DOACs: majorly non-valvular AF, pulmonary embolism or VTE | n=102 | DOACs   - apixaban (n=36) - dabigatran (n=1) - rivaroxaban (n=15)   (overall, 48% reduced DOAC dose) | VKA   - warfarin (n=20) - fluindione (n=30) | - bleeding events - major bleeding (clinically overt bleeding accompanied by 1 or more of the following: a drop in the haemoglobin level of 2 g/dL or more, transfusion of at least 2 units of blood, bleeding from a critical area, or fatal bleeding) - thromboembolic events (arterial thromboembolism event, including stroke, transient ischemic attack, and systemic embolism, and any VTE) - haemoglobin levels - drug-drug interactions | - DOAC: 14.1±13 months - VKA: 22.0±20 months | - No major or fatal bleeding events in DOAC group - Minor bleeding events in DOAC group: 13% (7/52), mainly subcutaneous and urogenital - Bleeding events significantly lower in DOACs groups [13% (7/52) in DOACs vs 42% (21/50) in VKAs, p=0.002] - Bleeding rate adjusted to exposure period significantly lower in DOACs group (11.5 versus vs 22.9 per 100 patient-y at risk, p=0.037) - Kaplan Meier curve: DOAC therapy associated with less bleeding risk vs. VKA (HR=0.39, 95%CI, 0.19-0.85, p=0.041) | - No incident stroke or transient ischemic attack in both groups - Incidence of pulmonary embolism or DVT: 0% DOAC vs 8% (4/50) VKA, p=0.054 | - 3/4 patients with a concomitant medication at risk of drug-drug interaction (strong inhibitor/inductor of CYP 3A4 or/and P-gp) had a bleeding event | high |
| Green et al, 2021, Ther Drug Mont | Single-centre prospective cohort study (years N/A) | adult KTRs  indications for DOACs: AF in all study patients | n=19 | Apixaban –group (n=19, 12 on 5 mg bid and 7 on 2,5 mg bid) | Apixaban - non-transplant controls (n=20) | - tacrolimus peak and trough levels - C0/D ratio - adverse events | N/A | - no severe bleeding events in KTRs | - no thromboembolic events in KTRs | - Mean tacrolimus trough level not different before and after apixaban initiation (8.5 ± 3.25 vs. 7.6 ± 2.83 ng/mL, p=0.3) - C0/D ratio before and after apixaban initiation not different (2.47 ± 1.5 vs. 2.33 ± 1.13 ng/mg, p=0.795) - 8/19 (42%) tacrolimus dose adjustment after apixaban initiation (7 reduction, 1 increase in tacrolimus dose) | low |
| Mansell et al, 2021, Clin Transl Sci | open-label drug–drug interaction study (years 2019-2020) | Adult transplant recipients | N=26 | single oral dose of 10 mg apixaban   - transplant recipients (n=14, 6 kidney and 8 lung) - healthy subjects (n=12) | N/A | outcomes for testing interaction between apixaban and tacrolimus of cyclosporine:   - Plasma apixaban concentration–time profiles - Pharmacokinetic parameters of apixaban (Cmax, AUC0-tlast, AUC0-inf, Tmax, t1/2, CL/F, Vz/F) | 72h | N/A | N/A | - Apixaban exposure was increased in transplant recipients receiving tacrolimus or cyclosporine, with AUC_₀–inf_ of 4312 and 5388, respectively, and GMRs for C_max_ and AUC of approximately 2–3-fold compared with healthy subjects | low |
| Parker et al, 2021, Clin Transplant | single centre retrospective study (years 2018-2020) | adult KTRs indications for DOACs: 18/31 VTE (4 DVT and 11 PE) and 15/31 non-valvular AF | n=31 | - apixaban (n=25, 5 on 2.5mg bid and 20 on 5mg bid - rivaroxaban (n= 5, 2 on 20mg od and 3 on 15mg od) - edoxaban (n=2, 1 on 30mg/d and 1 on 60mg/d-then switched to apixaban) | NA | - trough and peak DOAC levels within the expected ranges - thromboembolic events (new onset or recurrent VTE and new onset stroke) - bleeding events: a) major bleeding  b) clinically relevant non-major bleeding (CRNMB) | median 1 year (range 3 months - 6.2 years) | - 2 episodes of major bleeding (haematuria and haemoptysis both requiring admission to hospital with a haemoglobin drop >2g/dL) - 2 episodes of CNRMB (haematuria and rectal bleeding with a Hgb drop<2g/dL both requiring medical attention) - Overall bleeding rates: 12.9% (4/31) with 6.5% patients experiencing a major bleed, 6.9 per 100 patient-years at risk | - no thromboembolic events | - 94% (62/66) of DOAC levels were within the recommended ranges - 1 patient on apixaban 5mg bd with high trough and peak levels switched to warfarin - 5 deaths non-related to NOACs during follow-up | high |
| Pfrepper C et al, 2021, Transpl Int | single-centre two-part observational study (years 2015-2018) | Adult solid organ transplant recipients  Transplant type:   - 23 liver - 19 kidney - 5 combined   Indication for DOAC: AF 25 (53.2%), DVT or pulmonary embolism 16 (34.0%), portal vein thrombosis 5 (10.6%), thrombosis of cardiac bypass 1 (2.1%) | n = 47 | - rivaroxaban (n=26) - apixaban (n=20) - edoxaban (n=1) | RETROSPECTIVE PART   - Matched non-transplant controls on DOACs (n=47)   PROSPECTIVE PART   - Matched non-transplant controls on DOACs (n=47)   Transplant patients on VKAs (n=54) | - bleeding events (major, CNRMB) - thromboembolic events - DOAC levels | N/A | RETROSPECTIVE PART:   - No major bleeding   PROSPECTIVE PART   - Minor bleedings 17 patients (56.6%), more frequent under rivaroxaban (12/17, 70.6%) vs. apixaban (5/17, 29.4%, P = 0.04) - CRNMB 3/30 (10%) patients (all had epistaxis and patient concomitant haematuria due to cystic renal degeneration) - Minor bleeding in rivaroxaban vs VKAs: 12 (80%) vs 17 (89.5%), p=0.634. - No CNLMB in VKA group | RETROSPECTIVE PART:   - No thromboembolic events   PROSPECTIVE PART:   - No thromboembolic events | - Mean trough level change after DOAC initiation +18.8 ± 29.6% in transplant recipients vs +3.0 ± 16.5% in matched controls (p=0.004); no differences between immunosuppressive agents. - Levels of the immunosuppressive drugs within therapeutic range in all DOAC patients | high |
| Zaitoun et al, 2021, Transplant Proceed | Single-centre retrospective matched cohort study (years 2015-2019) | Adult KTRs indication for DOACs: AF (n=5), DVT (n=3) | n=16 | - Rivaroxaban (n=6, 1 on 30mg od, 3 on 20mg od and 2 on 15mg od) - Apixaban 10mg od (n=1) - Dabigatran 220mg od (n=1) | warfarin (n=8) | - thromboembolic events - bleeding events - C0/D change in patients on CNIs | N/A | - Patients with bleeding events: DOAC group 0/8 (0%) vs warfarin group 3/8 (37.5%), 1 subdural hemorrhage, 1 minor rectal bleeding, 1 hemorrhoid bleeding | - none | - NOAC group: no change in C0/D during the study period (p=0.34) | high |
| Firth et al, 2023, PLoS One | Single center retrospective cohort study (years 2011-2021) | adult KTRs | n=528 | - apixaban (n=189) - rivaroxaban (n=19) | warfarin (n=320) | - bleeding events: major bleeding (defined as ICH, GI bleeding involving haemorrhage, other forms of haemorrhage, and bleeding into critical sites such as ocular, pericardium, joint, etc.) - thromboembolic events: incident VTE, ischemic stroke | median follow-up 796 days (IQR 371, 1261) | - overall major bleeding (47 events) following initiation of anticoagulation non-significantly higher in warfarin vs DOAC group (10.6% vs 6.3%, p = 0.71) - incident major bleeding (8.4 vs. 5.3%, p = 0.89), GI bleeding (4.4% vs. 1.9%, p = 0.98) and ICH (1.9% vs. 1.4%, p = 0.85) non significantly higher in warfarin vs DOAC group - cumulative bleeding incidence at one year, two years, and three years post-transplant not different between DOAC and warfarin groups ( 3.6%, 5.2%, and 8.1% for DOACs vs 5.1%, 7.8% and 9.7% for warfarin) - in multivariable analysis, type of anticoagulation (DOAC vs warfarin) not a predictor of bleeding | - VTE [DOAC vs warfarin, 74 (35.6% vs 125 (39.1%), p=0.65] and ischemic stroke [11 (5.3%) vs 15 (4.7%), p=0.27] not different between DOAC and warfarin group | N/A | high |
| Santoro et al, 2024, Eur J Inter Med | prospective multicentric study (years 2016-2020) | Adult KTRs  indications for DOACs: non-valvular AF, pulmonary embolism or VTE, others | n = 116 | - apixaban (n=32) - edoxaban (n=17) - rivaroxaban (n=14) - dabigatran (n=3) | VKAs group (n=50) | - major bleeding (clinically overt bleeding accompanied by 1 or more of the following: a drop in the haemoglobin level of 2 g/dL or more, transfusion of at least 2 units of blood, bleeding from a critical area, or fatal bleeding) - thromboembolic events (stroke, transient ischemic attack, systemic embolism, and any venous embolic event) - drug-drug interactions - related adverse events | 18 months | - no differences in incidence of major bleeding (6% DOACs vs. 4% VKAs, p=0.69) | - no thromboembolic events in both groups | - After 14 days of DOACs therapy initiation, slight but not statistically significant increase of serum levels of tacrolimus (+0.19±0.67 p=0.80) and cyclosporine (+0.12±0.25 p=0.94) - No changes in C/D ratio of tacrolimus and cyclosporine (p=0.96, p=0.86, respectively). - No changes in serum levels (4.8±1.1 vs. 4.2±0.8, p=0.33) and C/D ratio of everolimus (3.6±1.6 vs. 3.15±1.4, p=0.64) - During 18-month long follow-up, stable serum levels and S.D ratio of tacrolimus and cyclosporin | Some concerns |

**Supplementary Table 13:** Summary Evidence Table for Question 4 – Is there a need for interruption of antiplatelet therapy before graft biopsy?

| **Author, year, Journal** | **Study design / Time frame** | **Patient characteristics** | **No. of patients** | **Antithrombotic drug and dosage** | **Comparator (if applicable)** | **Biopsy-related outcomes** | **Follow-up of the outcomes of interest** | **Results - Effect measure & estimates** | **Risk of bias** |
| --- | --- | --- | --- | --- | --- | --- | --- | --- | --- |
| Robertson et al, 2000, Nephrol Dial Transplant | single centre retrospective cohort study (years 1985-1997) | Adults KTRs (living and deceased-donor) | **n=955** | **PERIOD 07/1991-06/1997**  (n= 480)   - low-dose aspirin (75mg od) for 1-month post-transplant (if high risk patients, i.e., with diabetes, history of DVT or PAD, for long-term) - high-dose aspirin (150mg od) for long-term (in patients with previous renal artery or renal vein thrombosis) | **PERIOD 07/1985-06/1991** (n=475)  no antiplatelet | - Bleeding events | first 30 days posttransplant | - post-biopsy bleeding events (n=8, 1.6% of biopsies, compared to previous reports of 0.3-12.6%) | high |
| Murphy et al, 2001, Br J Surg | Single-centre consecutive case series (years N/A) | adult KTRs (cadaveric) | n=226 | aspirin 150mg od (n=105) | no aspirin (n=121) | - aspirin-related complications | 3 years | - incidence of macroscopic haematuria following graft biopsy not different between groups (9% vs 7% for aspirin vs control group, p=ns) | high |
| Li et al, 2016, J Am Coll Radiol | Single-centre prospective cohort study (years N/A) | adult KTRs | N= 880 | N/A | N/A | - Major complications - Minor complications | N/A | - Before (n=502) and after (n=378) implementation of the new biopsy protocols compared - 29 (5.8%) complications in pre-implementation group vs 11 (2.9%) in post-implementation group (p=0.04) | high |
| Morgan et al, 2016, Am J Transplant | Single-centre retrospective case-control study (years 2007-2012) | adult and paediatric KTRs | n=1654  (with 2514 biopsies) | *antiplatelet, anticoagulants stopped 7 days before biopsy unless deemed necessary* | N/A | - complications (transfusion, admission, interventional radiology procedure, surgical exploration, transplant loss) | 30 days after biopsy | - 47 patients had complications but no transplant loss - Univariate analysis: use of clopidogrel, heparin or warfarin associated with complications (p=0.05), but not use of aspirin (p-0.3) | high |
| Baffour et al, 2017, J Vasc Interv Radiol | Single centre retrospective cohort study (years 2005-2014) | adult KTRs who underwent allograft biopsy | n= 2362 (with 6700 biopsies) | Aspirin 81 mg or 325 mg within 10 days of the biopsy:   - 0–3 days (n=476) - 4–7 days (n=858) - 8–10 days (n=664) | No aspirin or aspirin use > 10 days before biopsy (n=4702) | - Major bleeding (perinephric bleeding requiring angiography, haematuria requiring continuous bladder irrigation) - Minor bleeding (perinephric bleeding/haematoma, and haematuria) | 3 months after biopsy | - Multivariate analysis: aspirin exposure not significantly associated to bleeding (p-0.192) [0–3 vs > 10/never, OR=1.67, 95%CI, 0.663-4.2; 4–7 vs > 10/never, OR=0.378, 95%CI, 0.0973-1.47; 8–10 vs > 10/never, OR=0.465, 95%CI, 0.107-2.02] - Multivariate GEE Model: 325 mg ASA ≤ 3 d significantly associated with any complications [OR=3.87, 95%CI, 1.12-13.4; p=0.032] and major complications [OR=6.30, 95%CI, 1.27, 31.3; p=0.024]. | high |
| Kuiper et al, 2018, Transplant Int | Single center Prospective cohort study (years N/A) | Adult KTRs | n=154 | ASA (n=26)  [stopped adequately 5 days prebiopsy in 25 of the subjects, 1 protocol violation] | Not ASA | - Haemoglobin drop of 1 mmol/l or more (equal to 1.61 g/dl) - perinephric and/or subcapsular haematoma in U/S - New-onset haematuria (>200 RBC/HPF) lasting longer than 24 h - Non-major (minor) bleeding complications: without need for intervention - Major bleeding complications: needing surgical intervention (e.g. embolization, coiling, explantation, transfusion, bladder catheterization etc.) | N/A | - 17 patients (11%) with a clinically relevant bleeding endpoint - ASA use was reported in 6 patients with bleeding event - ASA use linked to higher risk of bleeding events (OR=3.19, 95% CI, 1.06 to 9.61) | high |
| Mattiazzi et al, 2022, Kidney360 | Single-centre retrospective cohort study (years 2013-2016) | adult KTRs who underwent ultrasound-guided kidney transplant biopsy | n=573   - **Group 1**: biopsy performed by an interventional radiologist (IR) (n=447) - **Group 2**: biopsy performed by a transplant nephrologist (TN) (n=231) | N/A | N/A | - Non-severe post-biopsy complications [perinephric hematoma, hydronephrosis, AVF, haemoglobin (Hb) drop >2 g/dl, need for transfusion] - severe post-biopsy complications (page kidney, hydronephrosis, AVF with worsening renal function, perinephric hematoma associated with worsening renal function) | 30 days post procedure | - Patients with pre-biopsy medications: 41% on antiplatelet therapy and 6% on anticoagulation therapy (held 1 week before the biopsy) - 55 (8%) complications related to biopsies in the entire cohort - prior use of anticoagulation: significant predictor of complicated biopsies (adjusted OR=2.77; 95% CI, 1.06 to 6.37, p=0.02) and transfusion of blood products (adjusted OR=4.29; 95% CI, 1.17 to 12.56, p=0.01) | high |
